# Genome Evaluation Pipeline (GEP): a fully automated quality control tool for parallel evaluation of genome assemblies

**DOI:** 10.1093/bioadv/vbaf147

**Published:** 2025-06-26

**Authors:** James Sullivan, Diego De Panis, Valentina Galeone, Camila J Mazzoni

**Affiliations:** Berlin Center for Genomics in Biodiversity Research, Berlin, 14195, Germany; Department of Evolutionary Genetics, Leibniz Institute for Zoo and Wildlife Research, Berlin, 10315, Germany; Berlin Center for Genomics in Biodiversity Research, Berlin, 14195, Germany; Department of Evolutionary Genetics, Leibniz Institute for Zoo and Wildlife Research, Berlin, 10315, Germany; Berlin Center for Genomics in Biodiversity Research, Berlin, 14195, Germany; Department of Evolutionary Genetics, Leibniz Institute for Zoo and Wildlife Research, Berlin, 10315, Germany; Berlin Center for Genomics in Biodiversity Research, Berlin, 14195, Germany; Department of Evolutionary Genetics, Leibniz Institute for Zoo and Wildlife Research, Berlin, 10315, Germany

## Abstract

**Summary:**

The ability to generate high-quality genome assemblies is paramount for understanding global biodiversity. To streamline the quality control of the vast amount of assemblies currently being generated, we developed the Genome Evaluation Pipeline (GEP). Our Snakemake-based command-line tool is composed of two modes and was designed taking into consideration the recommendations of different international projects to standardize genome evaluation across the Tree of Life. With the Build Mode, GEP generates k-mer databases from high-accuracy sequencing reads, incorporating optional quality control and pre-processing steps. The Evaluate Mode leverages these databases to assess genome assembly quality using standard, gene content, and k-mer based metrics. Key features include the assessment of genome characteristics such as size, heterozygosity, and ploidy without reference sequences, and the comparison of k-mer profiles to evaluate assembly completeness and correctness. GEP also supports flexible input options for k-mer databases and the integration of Hi-C data for visual inspection of the assembly structure. Finally, our tool produces comprehensive reports summarizing contiguity, completeness, and correctness metrics of genome assemblies, facilitating their comparison and selection for downstream studies.

**Availability and implementation:**

GEP is publicly available as a Git repository at https://git.imp.fu-berlin.de/begendiv/gep.

## 1 Introduction

High-quality genome assemblies are essential for understanding global biodiversity in detail (Formenti *et al.* 2022). Fragmented or misassembled genomes typically fail to resolve structurally complex regions such as tandem repeats at telomeres and centromeres, segmental duplications, and other repetitive elements, as well as large structural variants (inversions, translocations, etc.) whose breakpoints lie in repeat-rich DNA. These deficits can truncate gene models, miscall copy-number variants, undermine gene, regulatory, and repeat annotation, and impede mapping of sequencing data, thereby weakening downstream phylogenomic and conservation analyses (Formenti *et al.* 2022). Advancements in whole-genome sequencing technologies have significantly reduced data production costs while markedly improving assembly algorithms and quality control (QC) procedures. Notably, the emergence of new generations of long-read sequencing technologies allowed not only the establishment and expansion of large-scale projects (e.g. Vertebrate Genomes Project, Darwin Tree of Life, European Reference Genome Atlas) but also individual research groups to scale their scope (Marx 2023).

Strikingly, most available genome assemblies are in a permanent draft status (Mukherjee *et al.* 2023). These assemblies have been generated with diverse combinations of data types and sequencing technologies, as well as different types of processing algorithms, over the course of decades. The comparative quality of many of these assemblies is uncertain, and the efficacy of downstream analyses on them is likely to vary (Chain *et al.* 2009). Because of this, selecting suitable reference genomes for a research project may constitute a critical task. Additionally, the iterative nature of a *de novo* assembly project produces several draft genomes across the steps of the chosen pipeline. Each of these steps, usually intermediate in the overall process, presents particularities that are reflected in their metrics. Identifying the best candidates for subsequent downstream stages constitutes an integral part of the workflow (Rhie *et al.* 2021).

One of the tasks of the Earth Biogenome Project (EBP), the overarching initiative that coordinates the generation of high-quality reference genomes for all eukaryotic life on Earth, is to establish quality metrics for achieving reference-quality genomes using state-of-the-art methods (Lewin *et al.* 2022). To ensure these metrics are met, up-to-date, standardized genome evaluation protocols must be consistently applied across the Tree of Life.

Several genome evaluation tools have been developed (Gurevich *et al.* 2013, Manchanda *et al.* 2020, Zhang *et al.* 2023), each addressing many different aspects of assembly quality assessment. However, these existing solutions have limitations regarding dependency management, versatility, scalability, or adherence to current quality standards. Consequently, a need remains for scalable, reproducible workflows designed for modern genome assembly evaluations.

Herein, we present GEP: the Genome Evaluation Pipeline, an all-in-one scalable and reproducible Snakemake workflow that incorporates the EBP-recommended analyses for high-quality reference genome QC. To showcase our tool, we illustrate two typical use cases: (i) quality benchmarking of existing publicly available genome assemblies in order to select the best candidates to be used as references for a downstream study and (ii) benchmarking a *de novo* genome assembly process.

## 2 Methods and implementation

GEP is a command-line tool developed using the workflow manager Snakemake to facilitate deployability. This Python-based engine provides a domain-specific language (DSL) for declaring rules, inputs, and outputs, automatically building and executing an optimized dependency graph across diverse computing environments. Workflows can run on a single computer or a high-performance computing (HPC) system using Slurm, adapting to the available resources and parallelization options. All software dependencies are automatically managed using Conda environments with defined versioning to ensure reproducibility (see Table 1, available as supplementary data at *Bioinformatics Advances* online for programs versions and authorship).

**Figure 1.**
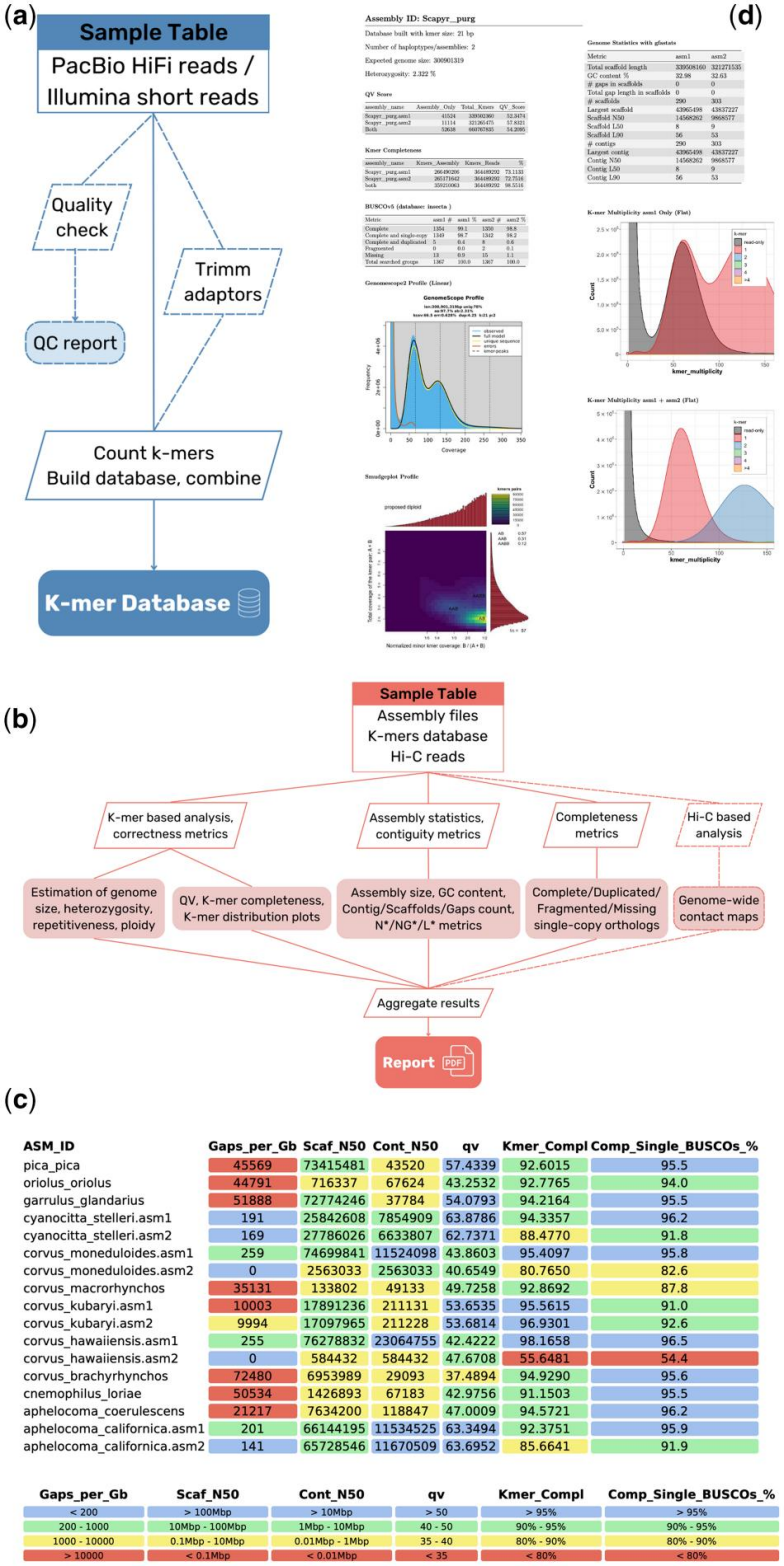
GEP general workflow and example results. Scheme of the workflow of Build Mode (a) and Evaluate Mode (b). Dotted lines correspond to optional steps. (c) Assemblies benchmarking case study. Final table of the report comparing all the species. (d) Full assembly pipeline evaluation case study. A page from the report illustrating one of the assembly stages as an example. See Supplementary document with the complete GEP pdf reports of the use cases.

The tool was designed to run in two distinctive modes (Fig. 1a and b. See further details in the Git repository README). The *Build Mode* generates k-mer databases from highly accurate reads (PacBio HiFi or Illumina Paired-End), including optional reads-QC and pre-processing steps. The *Evaluate Mode* assesses the quality of corresponding genome assemblies using standard contiguity, base, and gene content metrics, together with k-mer-based metrics, by using the databases produced during the Build Mode (or externally provided by the user). Execution of both modes is easily scalable to include multiple datasets (independent or related) within a single run, facilitated by the underlying Snakemake workflow manager.

### 2.1 Build mode

Although optional, reads pre-processing during Build Mode ensures that only biologically relevant k-mers are used by removing sequencing adapters. This way, Cutadapt can remove PacBio’s SMRTbell™ adapter present in the HiFi reads, and trimmomatic plus trimGalore can remove barcodes from 10x Genomics’ linked reads and other common Illumina short reads adapters. Similarly, FastQC, longQC, Nanoplot and MultiQC can also be optionally selected to assess the overall quality of the sequencing data.

K-mer distribution analysis is necessary for a robust assessment of assembly quality (Mapleson *et al.* 2017). In our pipeline, we use Meryl to generate a k-mer database for each independent set of reads to be used in downstream k-mer-based analyses. The database contains the k-mer coverage (the number of times a k-mer is found within the reads) for a user-defined k-length as well as the frequency of each coverage value.

### 2.2 Evaluate mode

This core section of the pipeline includes the execution of multiple tools for evaluating one or many genome assemblies, in parallel. The individual outputs of each tool are organized in a folder structure per evaluated assembly for streamlined accessibility. Finally, a user-friendly report is produced that condenses all the analysis for easy viewing and sharing.

A given genome assembly may comprise a single file (pseudo or collapsed haplotype) or two (phased, partially phased, or Primary and Alternate assemblies). For the latter scenario, the evaluation tools will run separately on each assembly, with the results being collected independently and displayed for a straightforward comparison.

Although some of the analyses in this mode require a k-mer database like the one produced in the Build Mode, users have the flexibility to use another one computed independently by providing its location filepath and corresponding k-length value.

#### 2.2.1 Genome profiling

Key genome characteristics such as size, heterozygosity, repetitiveness and ploidy are inferred using a reference-free approach based on the information stored in the k-mer database. Genomescope2 and Smudgeplot conduct a statistical analysis of k-mers in the reads, revealing information about genomic complexity and the overall quality of the sequencing data (e.g. error levels, potential contamination, sequencing biases and coverage).

#### 2.2.2 Assembly evaluation

We use several tools to generate comprehensive genome assembly metrics. Classic contiguity metrics and other assembly statistics, such as counts of Contigs, Scaffolds and Gaps, N*, NG* and L* values (e.g. NG50, N50 and L50), and GC Content, are obtained using gfastats. Completeness metrics are based on single-copy ortholog analysis for a clade related to the assembly’s species using BUSCO. Missing gene fractions can signal regions that were not assembled, while duplicated genes may indicate the need for purging. Users can define BUSCO’s lineage databases per assembly, providing flexibility to analyse assemblies from distant species in one run. Kmer-based analyses are performed using Merqury, which compares the assembly’s k-mer profile to the provided k-mer database. In addition to the Quality Value score (QV)—a metric of assembly correctness used to assess the accuracy of the nucleotide content—Merqury generates multiple k-mer distribution plots, offering insights into both retained haplotypes and missing sequences. In addition, it provides a quantitative metric termed k-mer completeness, which assesses the presence of k-mers within the assembly relative to the read set, thus shedding light on assembly completeness. Notably, in cases where two haplotypes are available, Merqury dynamically adjusts its analysis to leverage the complete dataset, thereby enhancing the accuracy of the metrics reported. Additionally, users can opt to provide HiFi reads to trigger the execution of Inspector, enabling the detection of structural errors in the assembly. Finally, they can include Hi-C reads to run an additional section of the pipeline to generate contact maps for manual assembly inspection and editing using Pretext.

#### 2.2.3 Final report

The last step constructs a PDF report that includes (i) a dedicated page for each assembly, displaying key results, (ii) optional Hi-C contact maps, and (iii) aggregated tables presenting results for all assemblies evaluated in a given run. The key results for each assembly contain contiguity, completeness and accuracy metrics, together with plots of genome profiling and k-mer distributions. Two aggregated tables are included at the end, applying colours to help assess the scores of each metric for efficient comparison of results for all assemblies in the run. The first table comprehensively summarizes all essential metrics for each assembly. The second table follows the assembly quality guidelines outlined by the Earth Genome Project (Howe *et al.* 2021).

## 3 Case studies

To showcase GEP, we analysed two different datasets illustrating possible use cases. The first dataset comprised assemblies and high-accuracy sequencing reads of twelve bird species belonging to the Corvidae family encompassing seven genera (see Table 2, available as supplementary data at *Bioinformatics Advances* online), with three tagged as ‘chromosome’ level and the rest as ‘scaffold’ level. Four assemblies also presented Hi-C data. We chose this non-model avian family of ecological and evolutionary interest because its genome assemblies span a realistic range of contiguity and data types and, with birds’ compact, low-repeat genomes and stable karyotypes, present both typical and challenging features for evaluation. The second dataset consisted of read data and assemblies at different stages of the hoverfly *Scaeva pyrastri* (BioProject accession: PRJEB42078). In this case, the sequencing data is composed of long (PacBio HiFi), short linked (10x) and Hi-C Illumina reads.

### 3.1 Case 1: assemblies benchmarking

Screening the quality of a dataset comprising different species to detect possible biases that could affect downstream analysis is a critical task. GEP’s results for the Corvidae datas *et al* lowed us to draw several conclusions (Fig. 1c. See Supplementary document with the complete GEP pdf report). Selecting genomes based solely on what NCBI displays can be problematic. The assembly-level categories (i.e. ‘Contig’, ‘Scaffold’, or ‘Chromosome’) are too general and sometimes do not correctly reflect the actual data. Moreover, no information is available on QV or Hi-C contact maps or with which to evaluate possible retained haplotypes. The same goes for BUSCO assembly scores (only annotation scores are available for annotated genomes, in this case, 4 out of 13 species). Based on our results, the best assemblies of this corvids dataset are *Aphelocoma californica*, *Corvus hawaiiensi*s and *C. moneduloides*. Interestingly, *A. californica* is tagged as ‘scaffold’ level assembly on NCBI, but its quality is instead in line with what would be expected of ‘chromosome’ level. Moreover, it is the only one of the sets that is haplotype-phased. Similarly, *Cyanocitta stelleri* would also correspond to ‘chromosome’ level according to our results, although the lack of manual curation based on the Hi-C contact map is evident.

As expected, we observed differences regarding contiguity across assemblies with long- and short-read data. Among the first group, it is worth highlighting the contrast between PacBio HiFi and CLR technologies, where the first yields consistently better results since it involves high-accuracy data. Noteworthy, lower QV scores in the assembly related to CLR were compensated in the case of *C. moneduloides* by polishing with short reads. Within the species that were assembled using only short reads, *Corvus kubaryi* used linked-reads technology (10x Genomics). The a priori expectation of producing better assemblies compared to non-linked-reads was verified by good contiguity metrics (e.g. total contigs or scaffolds and N50 values). Still, the assembly showed clear signs of haplotypic duplications and lower BUSCO-single scores in comparison to others. When comparing assemblies that incorporated only short-reads, *Pica pica* and *Aphelocoma coerulescens* exhibited the highest quality.

Based on our results, to prioritize assembly quality for downstream analysis involving as many species as possible, we would exclude *C. macrorhynchos* (low BUSCO), *C. kubaryi* (haplotypic duplications) and *C. brachyrhynchos* (low QV, also kmer multiplicity pointing to retained haplotypes).

### 3.2 Case 2a: full assembly pipeline evaluation

To demonstrate GEP’s utility during a *de novo* genome assembly production, we analysed all the intermediate stages of the complete assembly of *S. pyrastri* (Fig. 1d. See Supplementary document with the complete GEP pdf report). Evaluating the process through its different steps is crucial for quantifying incremental improvements and assessing the quality of the selected strategy.

The first step of the assembly pipeline produced two contig-level assemblies corresponding to each haplotype, where a clear difference was observed in size with ‘asm1’ showing twice the estimated with the profiling analysis. This, along with a very high BUSCO duplication score, points to a serious issue of haplotypic duplications. The next step of the protocol works to solve this problem. By purging the false duplicates and retained haplotypes, each assembly showed a much more balanced size and good BUSCO scores. Posteriorly, a HiC-scaffolding stage was carried out only on the purged ‘asm1’, where an increase in contiguity was observed, although without particularly improving other metrics. The evident effect of this step can be visualized in the Hi-C contact map, which improves substantially for ‘asm1’ and remains the same for ‘asm2’. The final step involved a polishing phase using short reads (10x Genomics) on both assemblies, with an expected increase in the QV score. A quick visual inspection of the Hi-C contact maps indicates the necessity of this assembly to undergo manual curation.

### 3.3 Case 2b: outcomes of contigging parameters tweaking

The hoverfly genome exemplifies how species with substantial heterozygosity, as indicated by our genome profiling results, may involve assembly challenges. Based on this, we used GEP to showcase its versatility to rapidly compare the effects of different parameters within a given stage of an assembly process. We evaluated different purging parameters (-l0 None, -l1 Light, -l2 Aggressive, -l3 Aggressive-high-het) and phasing modes (Primary/Alternate contigs or HiC-phased haplotypes 1/2) in Hifiasm (Cheng *et al.* 2022) during the contigging stage. When running without HiC-phasing mode, better results were obtained with more aggressive purging parameters. However only the Primary assembly showed good overall values for an assembly at this stage, and still a purging step involving the Alternate too would be required to fully address the purging issue. On the other hand, when using HiC-phasing mode, both light and aggressive purging parameters correctly solved the previous issue, showing balanced haplotypes 1 and 2 assembly sizes with good metrics in general. Among them, the assembly with light purging showed slightly better metrics overall.

## 4 Discussion

We developed a pipeline to streamline genome assembly quality evaluation. GEP uses widely adopted tools to produce standardized metrics used for quality control, with simplified installation and configuration, and robust execution, making it capable of processing a large number of assemblies without complexity and in a reproducible fashion.

Several tools have been developed to address genome assembly evaluation, each with their strengths and limitations compared to our approach. **QUAST** (Gurevich *et al.* 2013) is a widely recognized tool with a long trajectory in the field. It offers easy installation through a single Conda package (though some dependencies like BUSCO are not included) and produces contiguity metrics and plots (e.g. Nx) in user-friendly HTML reports. However, QUAST was developed with another approach, predates the establishment and consolidation of modern metrics, and has some limitations. It relies heavily on reference genomes for certain metrics (e.g. k-mer based completeness and correctness), which is problematic when evaluating newly sequenced organisms without available reference sequences or poorly studied taxa. Additionally, QUAST’s BUSCO lineage databases are restricted (eukaryota, fungi, bacteria), limiting its versatility and excluding simultaneous analyses across diverse taxa. **GenomeQC** (Manchanda *et al.* 2020) emerged as a user-friendly alternative integrating similar analyses as QUAST, yet suffers from limited maintenance, evidenced by outdated tools (e.g. BUSCO version 3 compared to the current version 5), and appears to no longer be actively developed. **GAEP** (Zhang *et al.* 2023) is a more recent alternative that incorporates several important evaluation features. However, it critically requires users to install and specify paths for all software dependencies, creating potential challenges for less experienced users and limiting its deployability.

In contrast, GEP offers several key advantages. Our pipeline efficiently handles large numbers of assemblies simultaneously, substantially reducing complexity and manual effort compared to executing individual tools separately. Powered by Snakemake, GEP ensures reproducibility, efficient resource management, and seamless software installation with version control via Conda. The pipeline also provides streamlined execution with robust error handling and troubleshooting capabilities. Notably, the catalogue of analyses available in GEP is strongly oriented towards EBP recommendations. A prime example is the incorporation of Hi-C contact maps for assembly visualization and manual inspection, a crucial feature for modern high-quality genome assemblies absent from other available tools.

A similar solution could theoretically be recreated using **Galaxy**, an open bioinformatics platform that enhances accessibility and reproducibility by providing free resources, consistent tool versions, and workflow-sharing capabilities, as exemplified by the Vertebrate Genomes Project pipelines for genome assembly (Larivière *et al.* 2024). Galaxy provides an intuitive graphical user interface, making bioinformatics more accessible to a broader user base. However, it relies on integrated tools and predefined parameters, which can be both a strength and a limitation. While its free online resources are convenient, they are restrictive for large, resource-intensive tasks. Although Galaxy can be installed locally to mitigate some limitations, the setup process is complex and non-trivial, particularly in computer clusters.

Certain functionalities in GEP, such as the comprehensive aggregation of results into PDF reports based on EBP-recommended metric thresholds, are not natively available in Galaxy and would require porting or custom development. To enhance portability, we plan to integrate containerization (e.g. Docker or Apptainer, both natively supported by Snakemake) to simplify deployment and improve reproducibility across diverse computational environments. Additionally, ongoing development will focus on regular updates, expanding the toolset, refining analytical outputs, and maintaining alignment with evolving standards in genome assembly evaluation.

## 5 Conclusion

GEP simplifies the assessment of genome assembly quality, facilitating informed decisions in reference selection and benchmarking. With its scalable design, EBP-recommended analyses, and comprehensive reports, our tool adds critical standardization to the reference data generation process and their utilization, crucial for understanding global biodiversity through genomics.

## Supplementary Material

vbaf147_Supplementary_Data

## Data Availability

The data underlying this article are available in *begendiv/gep* at https://git.imp.fu-berlin.de/begendiv/gep.
