# Supplementary material for: Genome Evaluation Pipeline (GEP): a fully automated quality control tool for parallel evaluation of genome assemblies
Source: Bioinform Adv. 2025 Jun 26;5(1):vbaf147. doi: 10.1093/bioadv/vbaf147 (PMC12296351; doi:10.1093/bioadv/vbaf147)
Supplement: vbaf147_Supplementary_Data [file vbaf147_supplementary_data.pdf]

Genome Evaluation Pipeline (GEP): A fully-automated quality control tool for parallel evaluation of genome assemblies

SUPPLEMENTARY DOCUMENT

This document contains Supplementary Tables 1 and 2, and the complete GEP pdf reports of the use cases.

Supplementary Table 1. Main software employed for assembly analysis in GEP.

Tool	Version	Reference
Snakemake	7.30.2	Mölder et al. 2021 (doi:10.12688/f1000research.29032.2)
Cutadapt	4.8	Martin 2011 (doi:10.14806/ej.17.1.200)
Trimmomatic	0.39	Bolger et al. 2014 (doi:10.1093/bioinformatics/btu170)
TrimGalore	0.6.7	Martin 2011 (doi:10.14806/ej.17.1.200)
FastQC	0.12.1	Andrews 2010 (url:bioinformatics.babraham.ac.uk/projects/fastqc)
LongQC	1.2.1	Fukasawa et al. 2020 (doi: 10.1534/g3.119.400864)
Nanoplot	1.44.1	De Coster et al. 2023 (doi: 10.1093/bioinformatics/btad311)
MultiQC	1.13	Ewels et al. 2016 (doi:10.1093/bioinformatics/btw354)
Meryl	1.3	Rhie et al. 2020 (doi:10.1186/s13059-020-02134-9)
Genomescope2	2.0	Ranallo-Benavidez et al. 2020 (doi:10.1038/s41467-020-14998-3)
Smudgeplot	0.2.5	Ranallo-Benavidez et al 2020 (doi:10.1038/s41467-020-14998-3)
Merqury	1.3	Rhie et al. 2020 (doi:10.1186/s13059-020-02134-9)
gfastats	1.3.6	Formenti et al. 2022 (doi:10.1093/bioinformatics/btac460)
BUSCO	5.4.7	Simão et al. 2015 (doi:10.1093/bioinformatics/btv351)
Inspector	1.3	Chen at al. 2021 (doi: 10.1186/s13059-021-02527-4)
bwa-mem2	2.2.1	Vasimuddin et al. 2019 (doi:10.1109/IPDPS.2019.00041)
Pretext	0.1.9	Harry (n.d.) (url:github.com/sanger-tol/PretextMap)

Supplementary Table 2. Corvidae dataset. Sequencing data highlighted in bold was used for k-mer profiling.

Species name	Assembly Type	GenBank Accession	NCBI's Assembly Level	Main Sequencing Data
Aphelocoma californica	Partially-phased	GCA_028536675.1 GCA_028536645.1	Scaffold	**PacBio HiFi** + Hi-C
Aphelocoma coerulescens	Collapsed	GCA_013398375.1	Scaffold	**Illumina PE** + Illumina MP
Cnemophilus loriae	Collapsed	GCA_013397755.1	Scaffold	**Illumina PE** + Illumina MP
Corvus brachyrhynchos	Collapsed	GCF_000691975.1	Scaffold	**Illumina PE**
Corvus hawaiiensis	Primary / Alternate	GCF_020740725.1 GCA_020740675.1	Chromosome	**PacBio HiFi** + Hi-C
Corvus kubaryi	Primary / Alternate	GCF_017639235.1 GCA_017639225.1	Scaffold	**Illumina PE (10x Genomics)**
Corvus macrorhynchos	Collapsed	GCA_014363025.1	Scaffold	**Illumina PE**
Corvus moneduloides	Primary / Alternate	GCF_009650955.1 GCA_009651025.1	Chromosome	PacBio CLR + **Illumina PE (10x Genomics)** + Hi-C
Cyanocitta stelleri	Primary / Alternate	GCA_026167965.1 GCA_026168045.1	Scaffold	**PacBio HiFi** + Hi-C
Garrulus glandarius	Collapsed	GCA_028565035.1	Scaffold	**Illumina PE**
Oriolus oriolus	Collapsed	GCA_013400235.1	Scaffold	**Illumina PE**
Pica pica	Collapsed	GCA_025802055.1	Scaffold	**Illumina PE**

Case 1: Assemblies benchmarking

Assembly ID: pica_pica

Database built with kmer size: 21 bp

Number of haploptypes/assemblies: 1

Expected genome size: 1109074616

Heterozygosity: 0.461 %

QV Score

assembly_name	Assembly_Only	Total_Kmers	QV_Score
pica_pica.asm1	39732	1047908288	57.4339
ALT_missing	0	0	nan
Both	39732	1047908288	57.4339

Kmer Completeness

assembly_name	Kmers_Assembly	Kmers_Reads	%
pica_pica.asm1	998503746	1078279972	92.6015
ALT_missing	0	1078279972	0
both	998503746	1078279972	92.6015

BUSCOv5 (database: passeriformes)

Metric	asm1 #	asm1 %
Complete	10370	95.6
Complete and single-copy	10355	95.5
Complete and duplicated	15	0.1
Fragmented	89	0.8
Missing	385	3.6
Total searched groups	10844	100.0

Genomescope2 Profile (Linear)

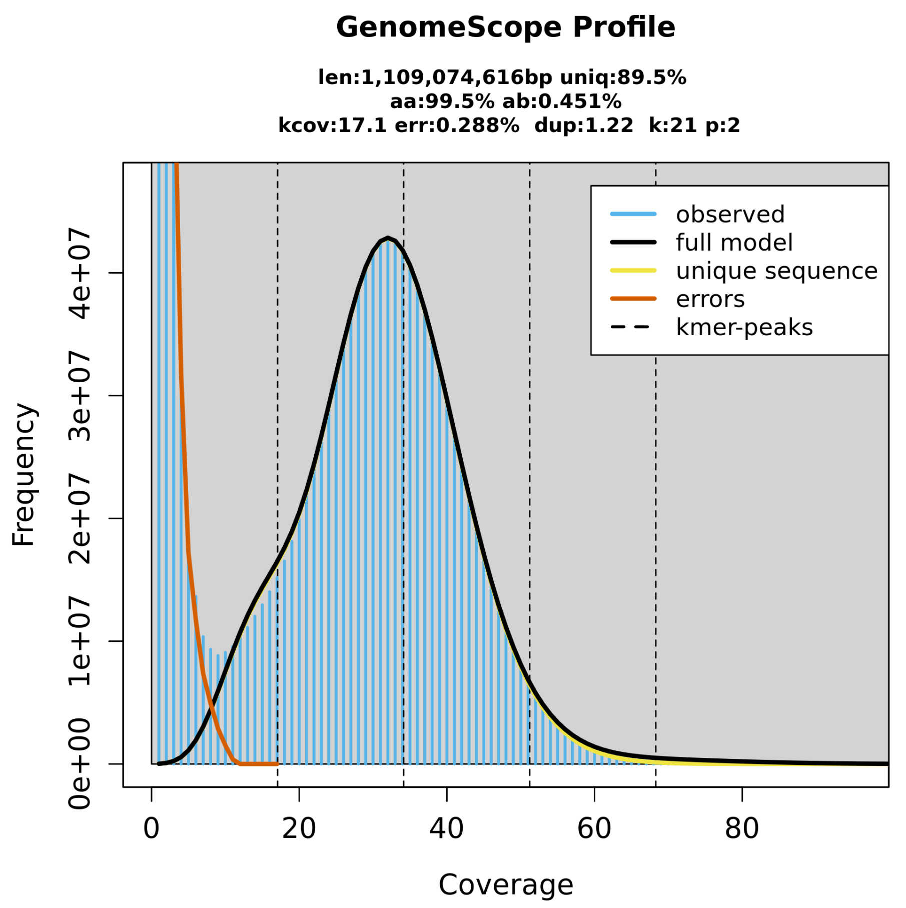

Genomescope2 Profile (Log)

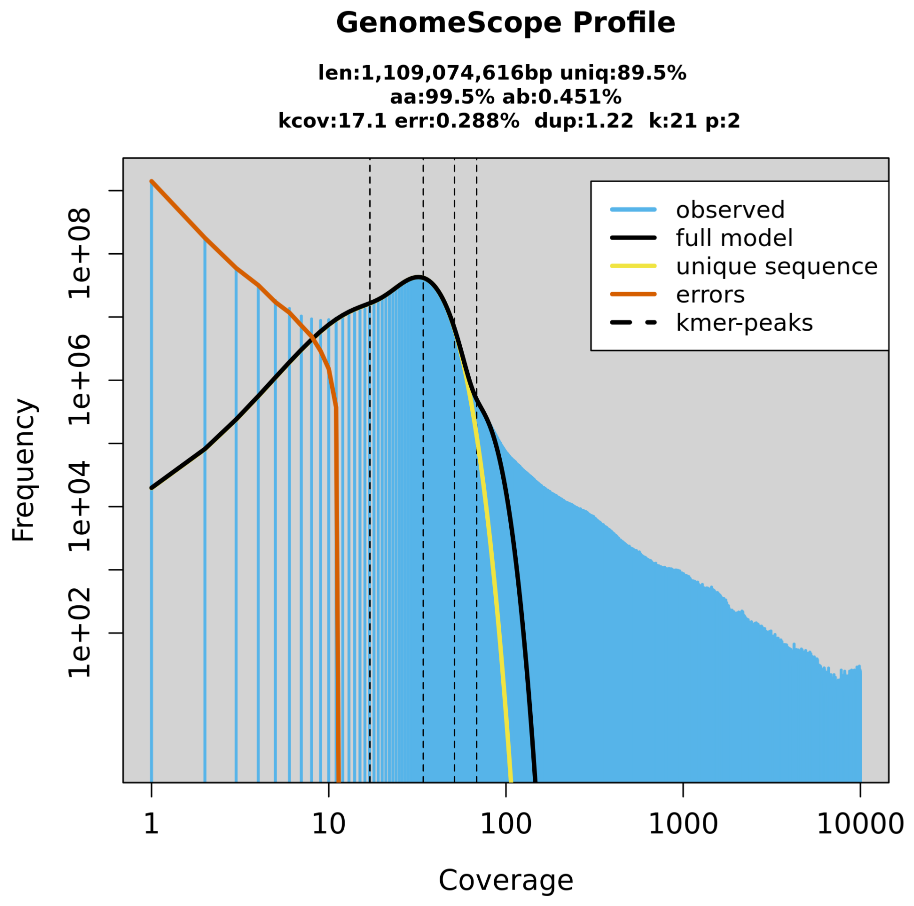

Genome Statistics with gfastats

Metric	asm1
Total scaffold length	1064369002
GC content %	42.10
# gaps in scaffolds	48503
Total gap length in scaffolds	14997363
# scaffolds	24674
Largest scaffold	153442850
Scaffold N50	73415481
Scaffold L50	5
Scaffold L90	21
# contigs	73177
Largest contig	567729
Contig N50	43520
Contig L50	6859
Contig L90	26601

K-mer Multiplicity asm1 Only (Stacked)

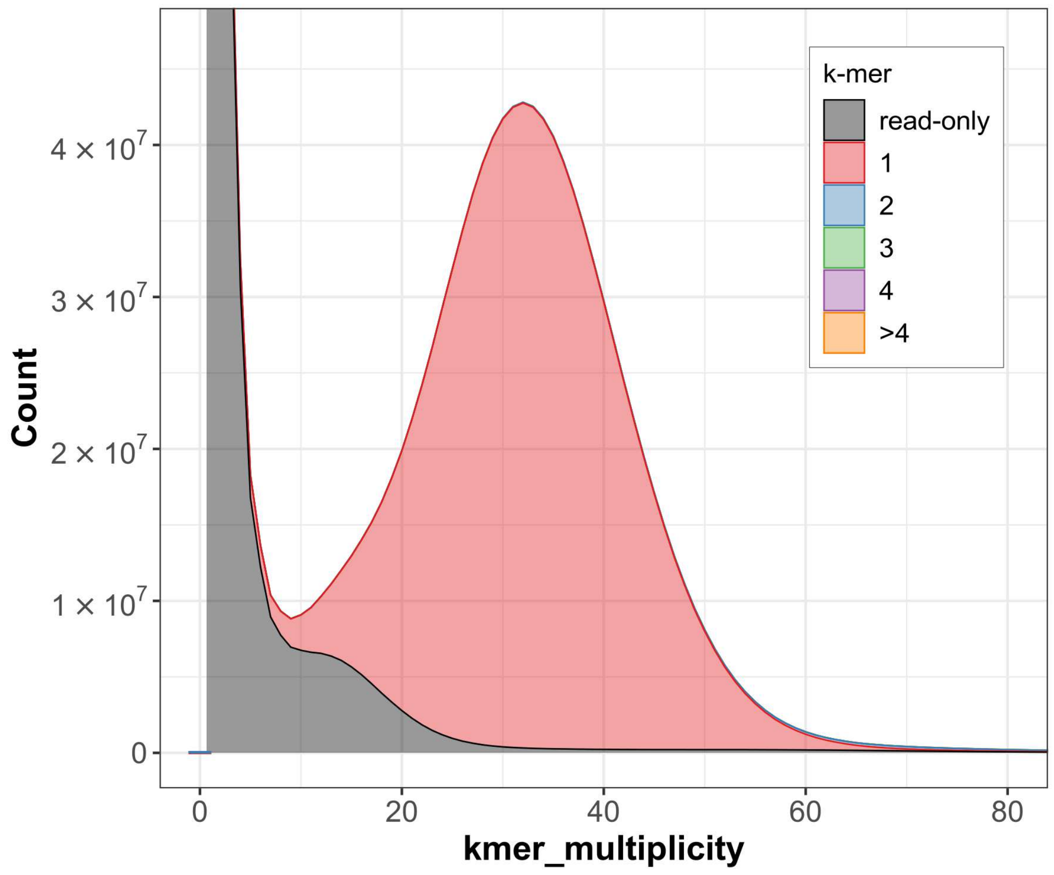

Assembly ID: oriolus_oriolus

Database built with kmer size: 21 bp

Number of haploptypes/assemblies: 1

Expected genome size: 1065128996

Heterozygosity: 0.707 %

QV Score

assembly_name	Assembly_Only	Total_Kmers	QV_Score
oriolus_oriolus.asm1	1022652	1030469684	43.2532
ALT_missing	0	0	nan
Both	1022652	1030469684	43.2532

Kmer Completeness

assembly_name	Kmers_Assembly	Kmers_Reads	%
oriolus_oriolus.asm1	995027160	1072499282	92.7765
ALT_missing	0	1072499282	0
both	995027160	1072499282	92.7765

BUSCOv5 (database: passeriformes)

Metric	asm1 #	asm1 %
Complete	10215	94.2
Complete and single-copy	10189	94.0
Complete and duplicated	26	0.2
Fragmented	144	1.3
Missing	485	4.5
Total searched groups	10844	100.0

Genomescope2 Profile (Linear)

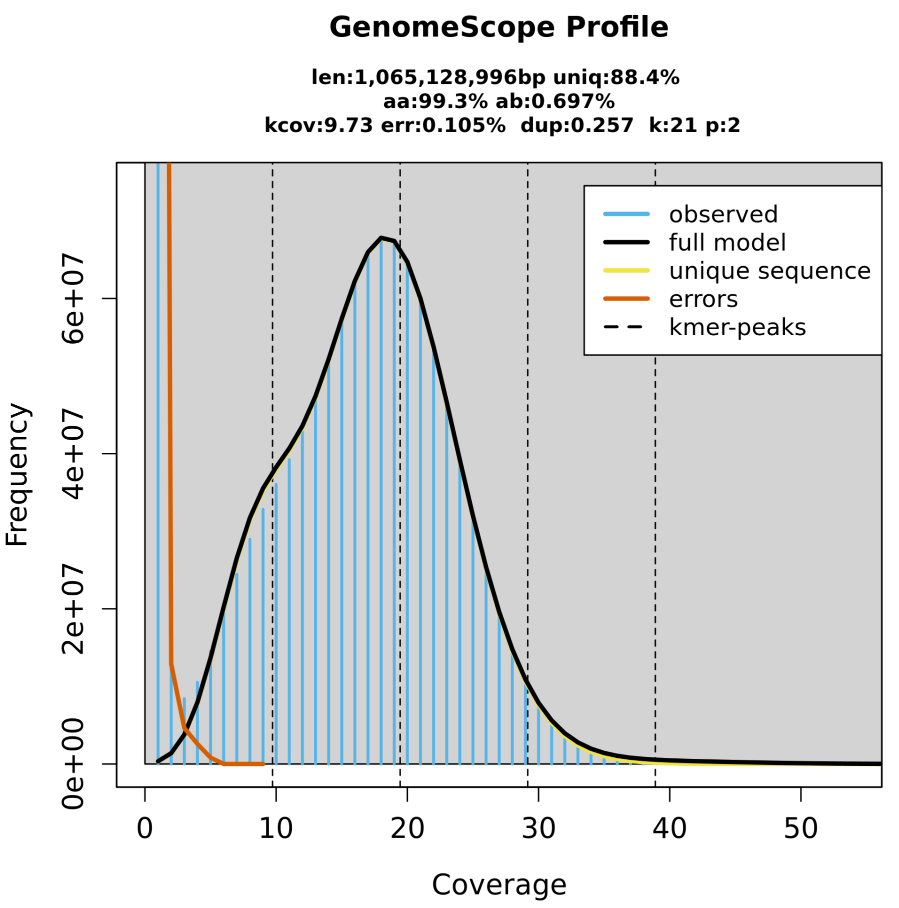

Genomescope2 Profile (Log)

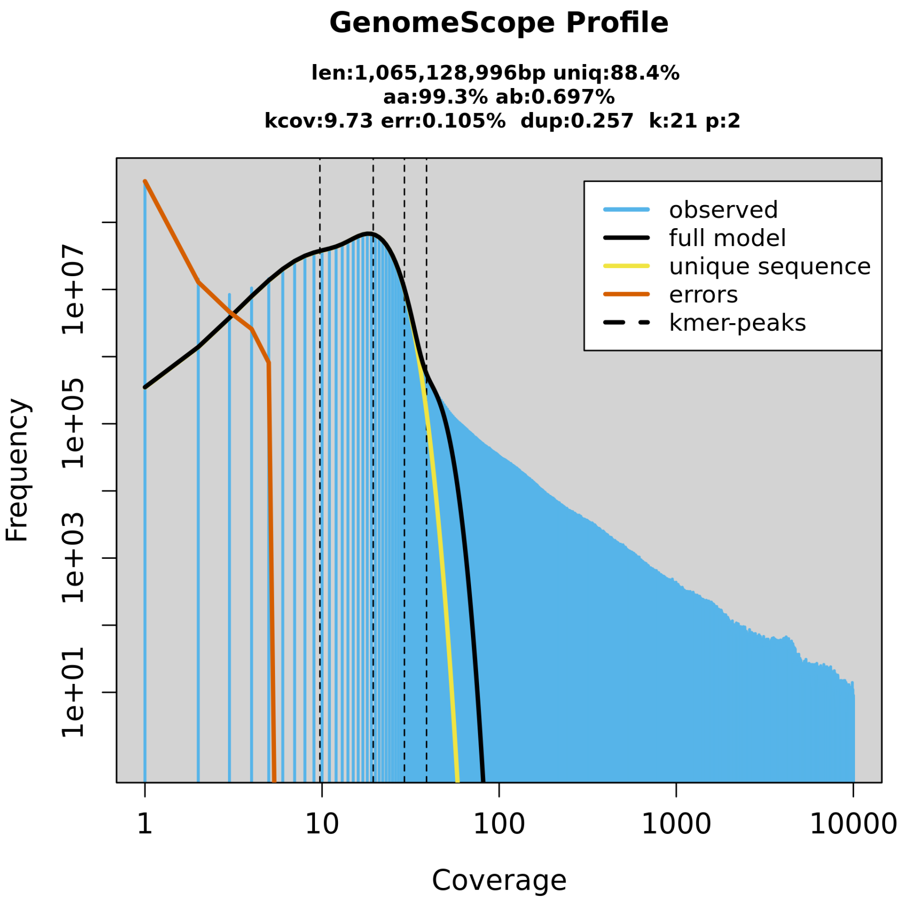

Genome Statistics with gfastats

Metric	asm1
Total scaffold length	1038866468
GC content %	42.44
# gaps in scaffolds	46532
Total gap length in scaffolds	7024624
# scaffolds	22076
Largest scaffold	4327510
Scaffold N50	716337
Scaffold L50	377
Scaffold L90	1982
# contigs	68608
Largest contig	513590
Contig N50	67624
Contig L50	4217
Contig L90	17169

K-mer Multiplicity asm1 Only (Stacked)

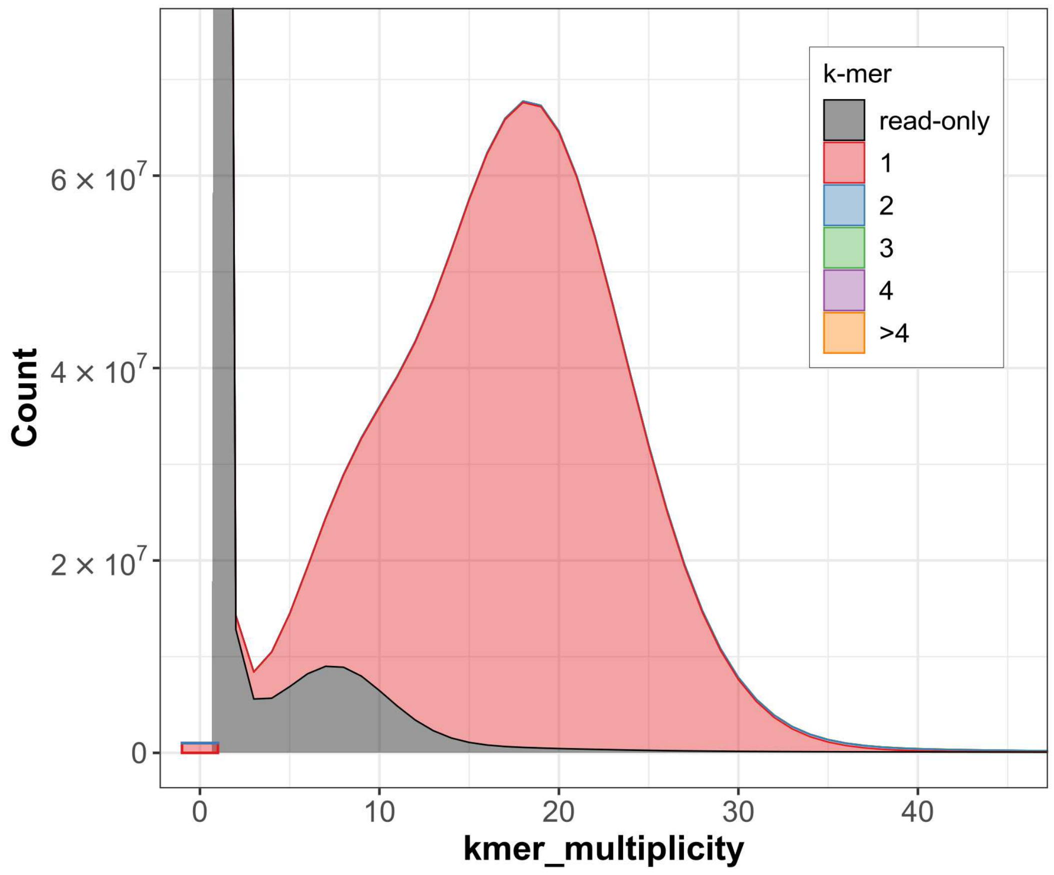

Assembly ID: garrulus_glandarius

Database built with kmer size: 21 bp

Number of haploptypes/assemblies: 1

Expected genome size: 1142507207

Heterozygosity: 0.406 %

QV Score

assembly_name	Assembly_Only	Total_Kmers	QV_Score
garrulus_glandarius.asm1	88983	1084009265	54.0793
ALT_missing	0	0	nan
Both	88983	1084009265	54.0793

Kmer Completeness

assembly_name	Kmers_Assembly	Kmers_Reads	%
garrulus_glandarius.asm1	1001343470	1062812301	94.2164
ALT_missing	0	1062812301	0
both	1001343470	1062812301	94.2164

BUSCOv5 (database: passeriformes)

Metric	asm1 #	asm1 %
Complete	10388	95.8
Complete and single-copy	10356	95.5
Complete and duplicated	32	0.3
Fragmented	91	0.8
Missing	365	3.4
Total searched groups	10844	100.0

Genomescope2 Profile (Linear)

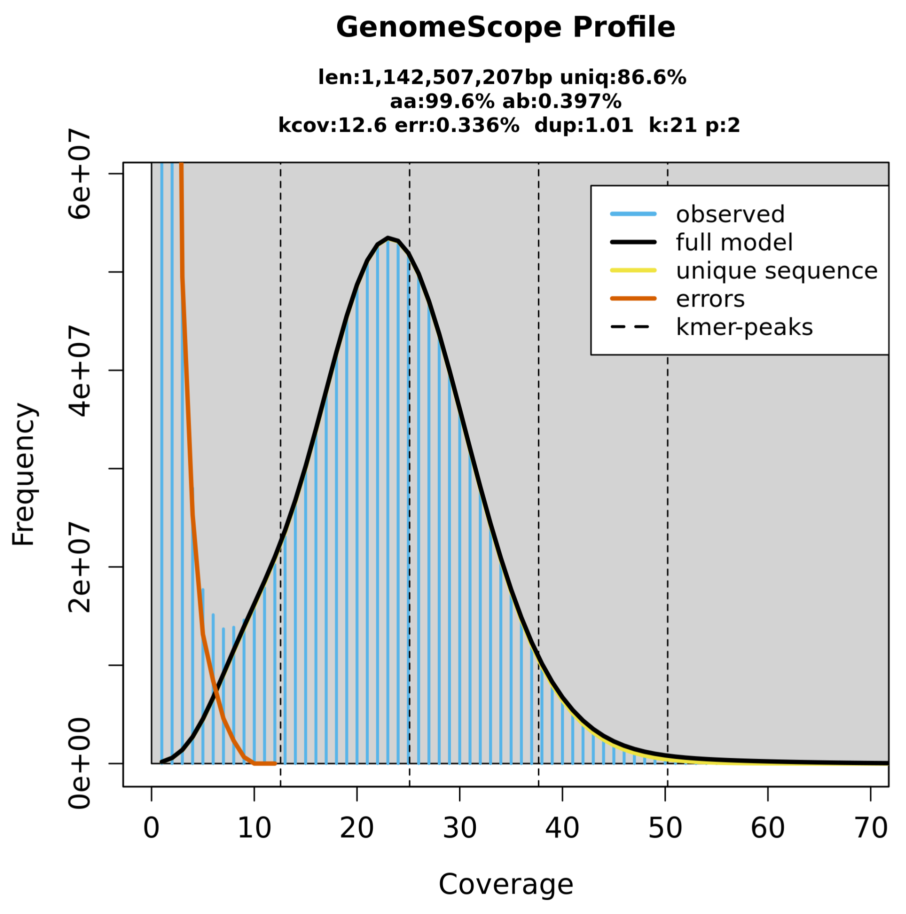

Genomescope2 Profile (Log)

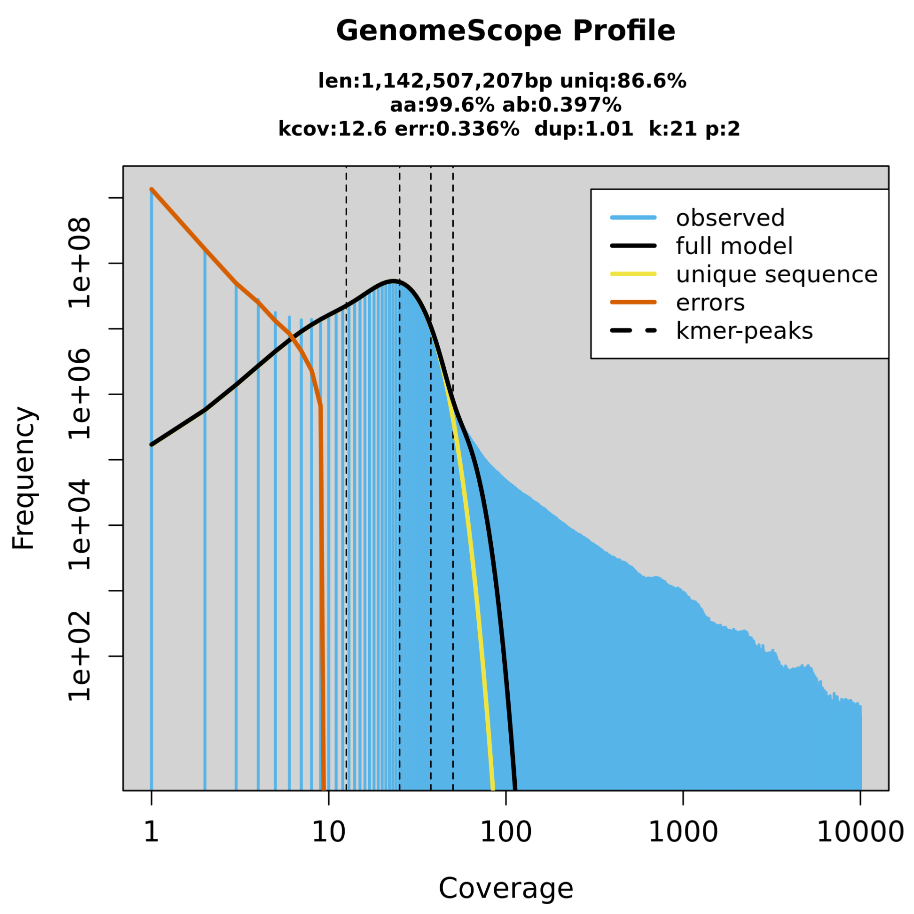

Genome Statistics with gfastats

Metric	asm1
Total scaffold length	1106508028
GC content %	41.49
# gaps in scaffolds	57415
Total gap length in scaffolds	20377340
# scaffolds	48662
Largest scaffold	153777791
Scaffold N50	72774246
Scaffold L50	6
Scaffold L90	23
# contigs	106077
Largest contig	328506
Contig N50	37784
Contig L50	8070
Contig L90	33891

K-mer Multiplicity asm1 Only (Stacked)

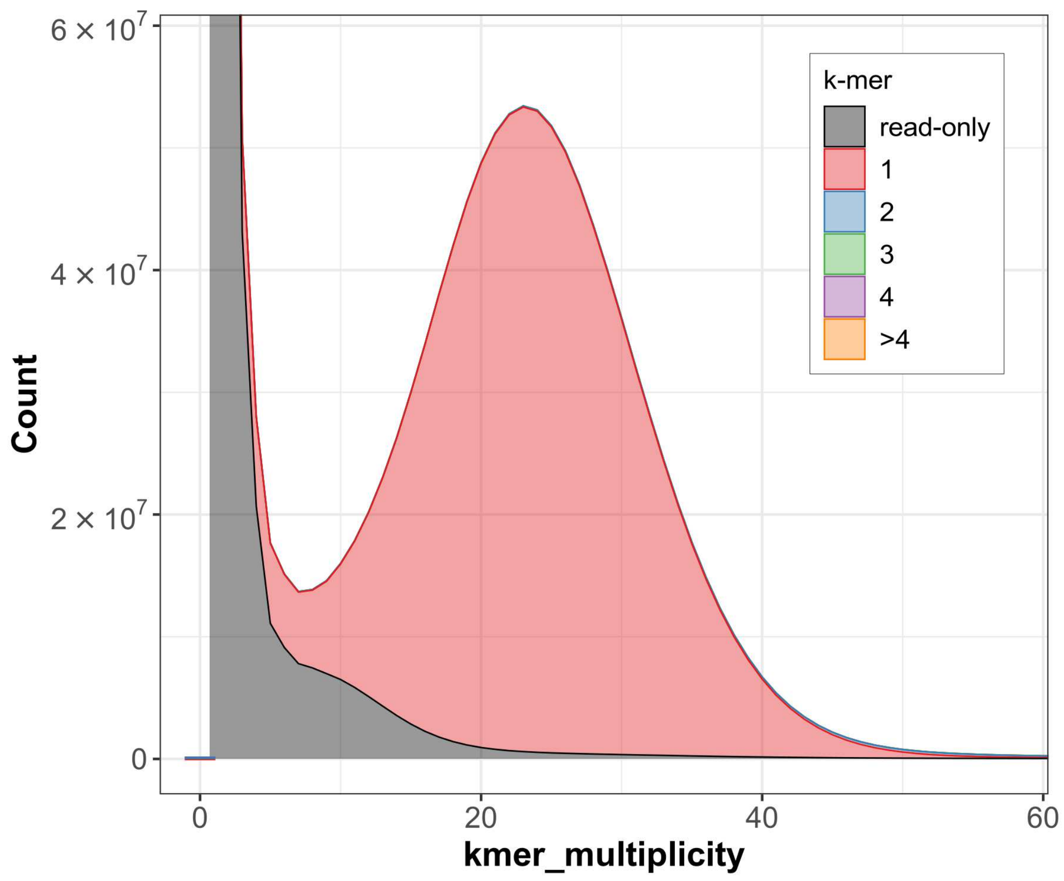

Assembly ID: cyanocitta_stelleri

Database built with kmer size: 31 bp

Number of haploptypes/assemblies: 2

Expected genome size: 1023233301

Heterozygosity: 0.647 %

QV Score

assembly_name	Assembly_Only	Total_Kmers	QV_Score
cyanocitta_stelleri.asm1	9998	1162931153	63.8786
cyanocitta_stelleri.asm2	13166	1177451771	62.7371
Both	23164	2340382924	63.2669

Kmer Completeness

assembly_name	Kmers_Assembly	Kmers_Reads	%
cyanocitta_stelleri.asm1	1009183490	1069778492	94.3357
cyanocitta_stelleri.asm2	946508296	1069778492	88.477
both	1065511257	1069778492	99.6011

BUSCOv5 (database: passeriformes)

Metric	asm1 #	asm1 %	asm2 #	asm2 %
Complete	10461	96.4	9985	92.1
Complete and single-copy	10436	96.2	9951	91.8
Complete and duplicated	25	0.2	34	0.3
Fragmented	57	0.5	55	0.5
Missing	326	3.1	804	7.4
Total searched groups	10844	100.0	10844	100.0

Genomescope2 Profile (Linear)

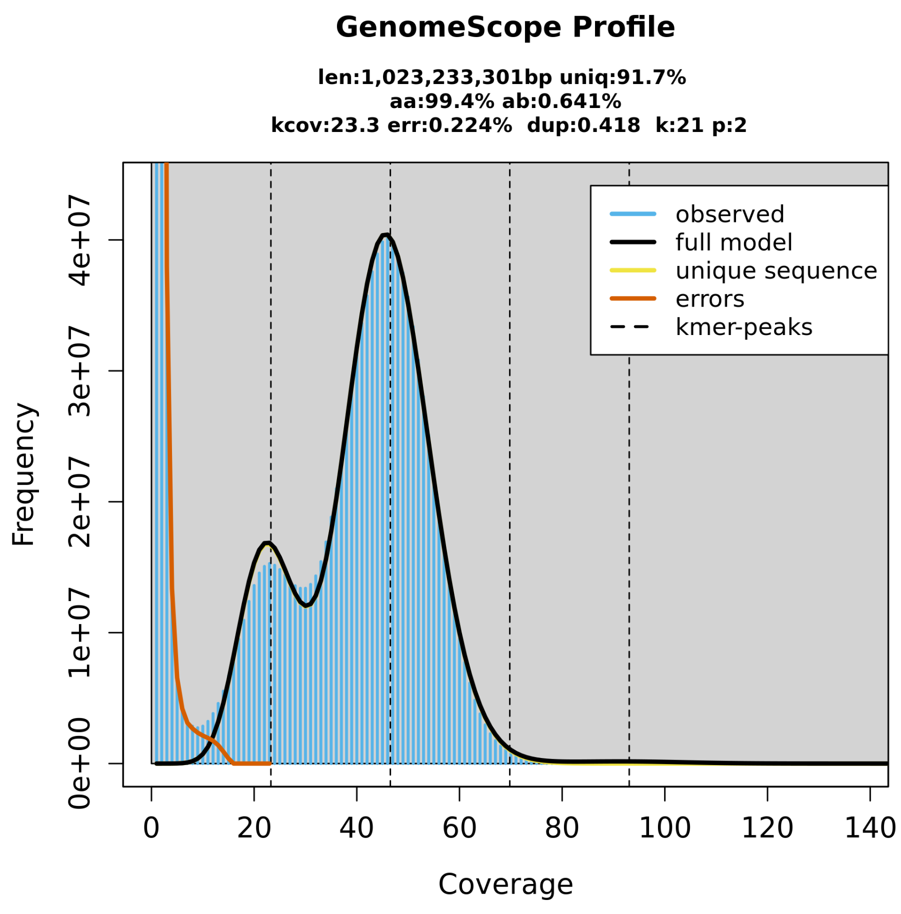

Genomescope2 Profile (Log)

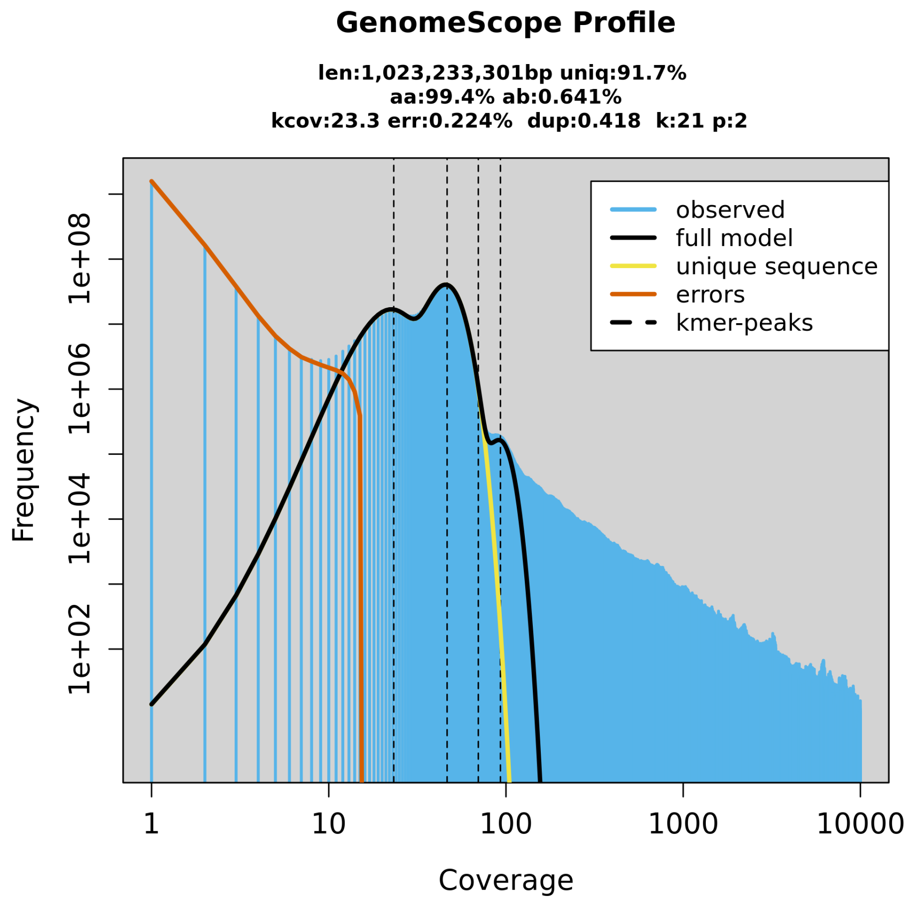

Genome Statistics with gfastats

Metric	asm1	asm2
Total scaffold length	1162964953	1177494911
GC content %	43.18	44.10
# gaps in scaffolds	223	200
Total gap length in scaffolds	22300	20000
# scaffolds	352	957
Largest scaffold	88620470	86024699
Scaffold N50	25842608	27786026
Scaffold L50	11	11
Scaffold L90	64	111
# contigs	575	1157
Largest contig	48674044	40161822
Contig N50	7854909	6633807
Contig L50	45	44
Contig L90	171	251

K-mer Multiplicity asm1 Only (Stacked)

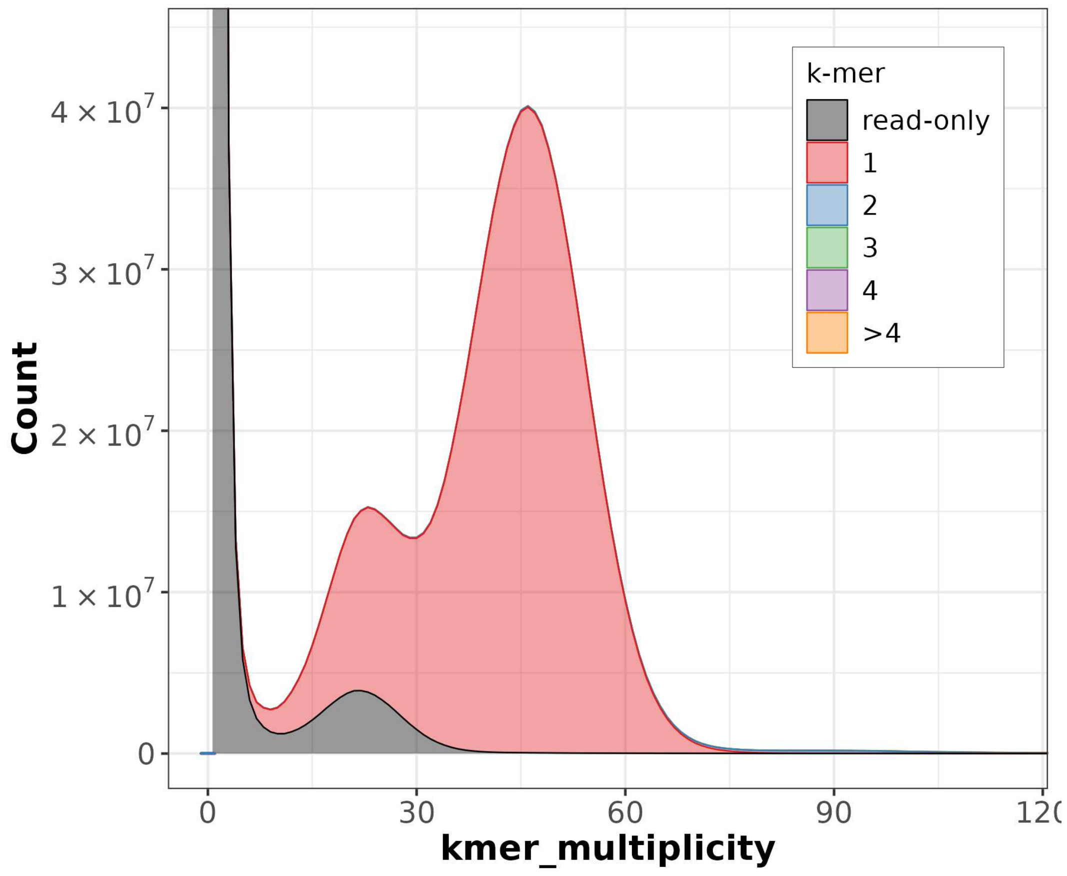

K-mer Multiplicity asm1 + asm2 (Stacked)

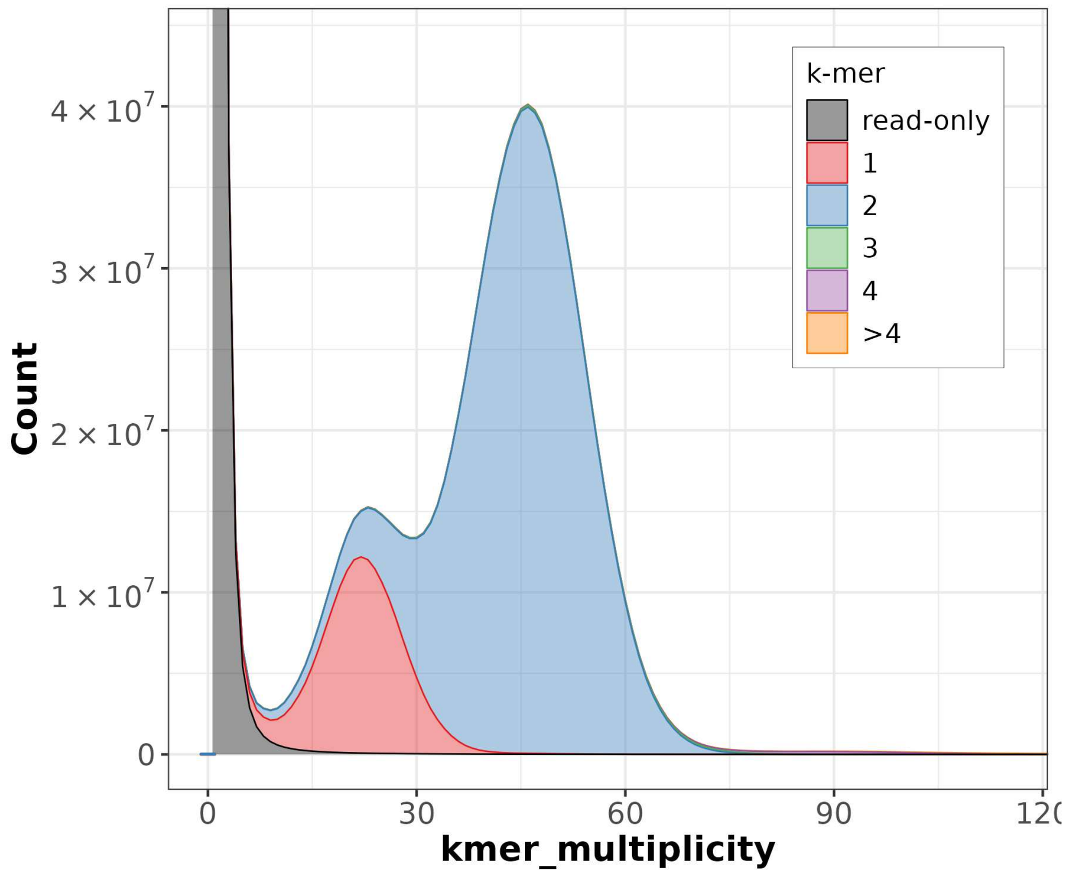

Assembly ID: corvus_moneduloides

Database built with kmer size: 21 bp

Number of haploptypes/assemblies: 2

Expected genome size: 1058828845

Heterozygosity: 0.45 %

QV Score

assembly_name	Assembly_Only	Total_Kmers	QV_Score
corvus_moneduloides.asm1	935372	1083856111	43.8603
corvus_moneduloides.asm2	1633405	905180377	40.6549
Both	2568777	1989036488	42.1087

Kmer Completeness

assembly_name	Kmers_Assembly	Kmers_Reads	%
corvus_moneduloides.asm1	1011329430	1059986510	95.4097
corvus_moneduloides.asm2	856098166	1059986510	80.765
both	1038266789	1059986510	97.9509

BUSCOv5 (database: passeriformes)

Metric	asm1 #	asm1 %	asm2 #	asm2 %
Complete	10436	96.3	8986	82.8
Complete and single-copy	10387	95.8	8959	82.6
Complete and duplicated	49	0.5	27	0.2
Fragmented	62	0.6	81	0.7
Missing	346	3.1	1777	16.5
Total searched groups	10844	100.0	10844	100.0

Genomescope2 Profile (Linear)

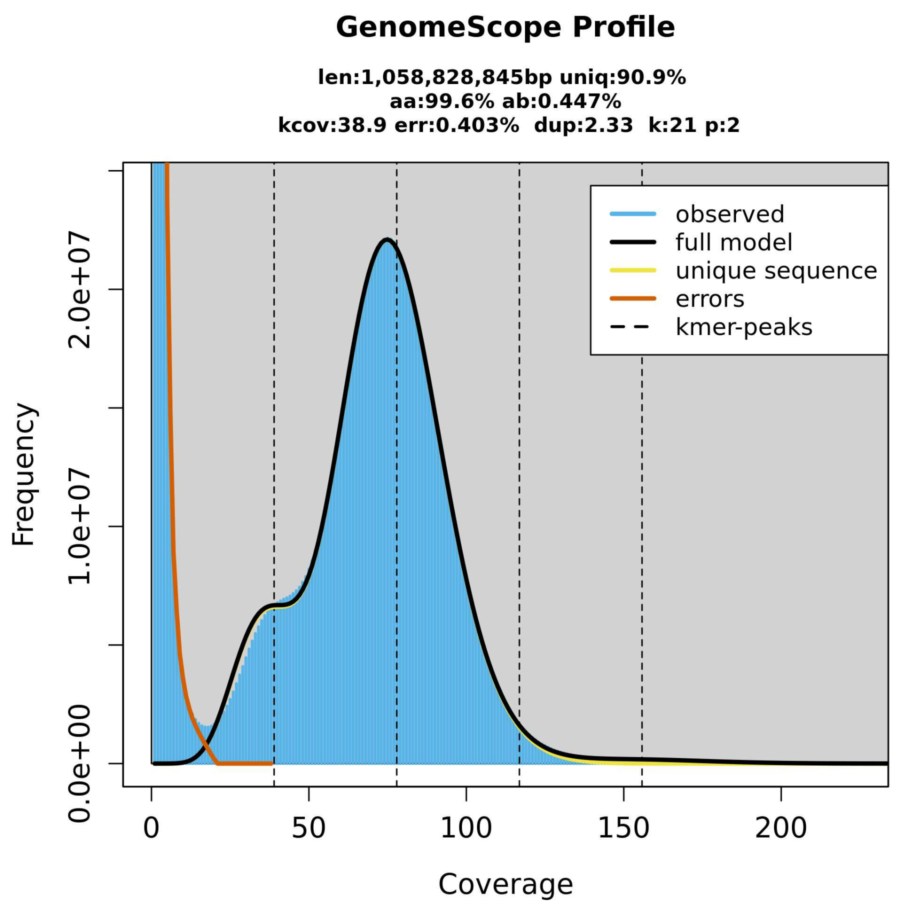

Genomescope2 Profile (Log)

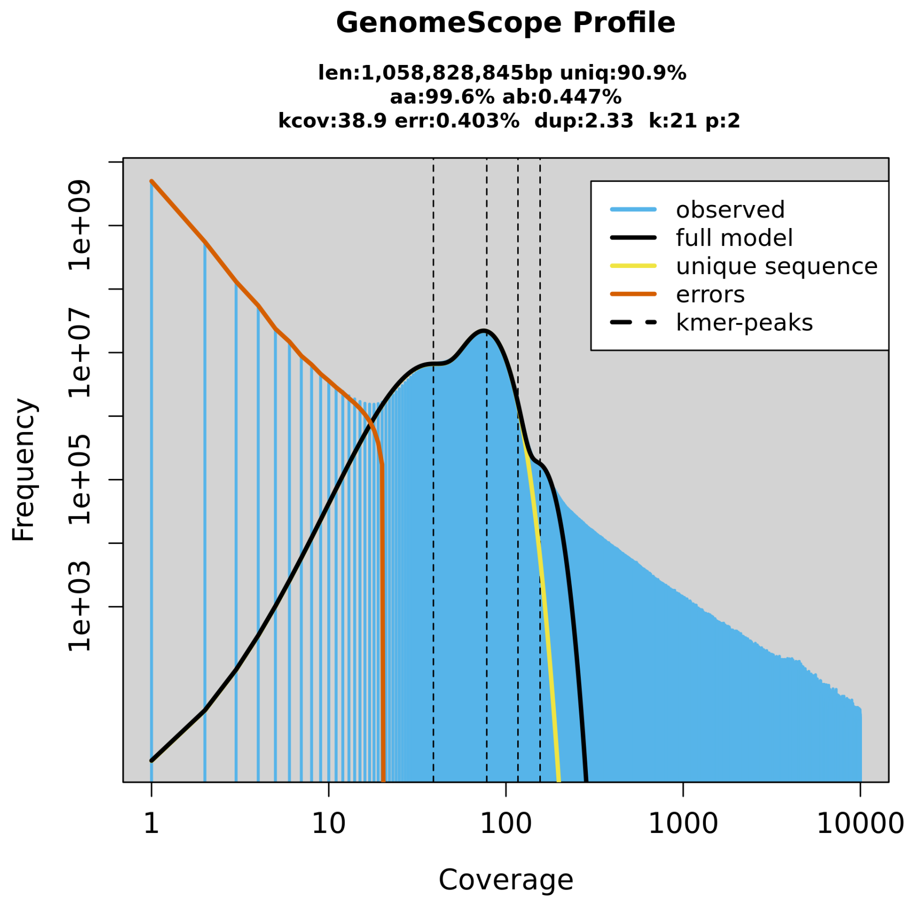

Genome Statistics with gfastats

Metric	asm1	asm2
Total scaffold length	1112734206	905198257
GC content %	42.57	42.67
# gaps in scaffolds	289	0
Total gap length in scaffolds	28870195	0
# scaffolds	106	894
Largest scaffold	165740467	12641944
Scaffold N50	74699841	2563033
Scaffold L50	5	105
Scaffold L90	20	381
# contigs	395	894
Largest contig	46782926	12641944
Contig N50	11524098	2563033
Contig L50	30	105
Contig L90	104	381

K-mer Multiplicity asm1 Only (Stacked)

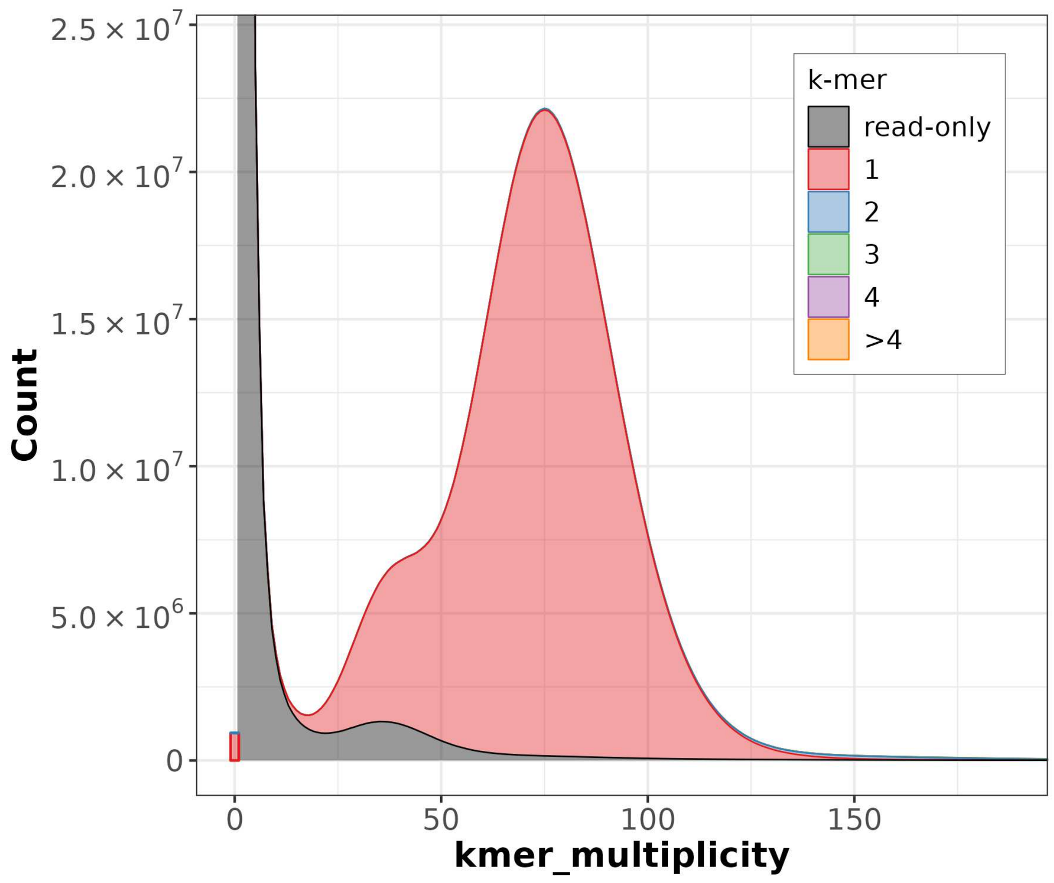

K-mer Multiplicity asm1 + asm2 (Stacked)

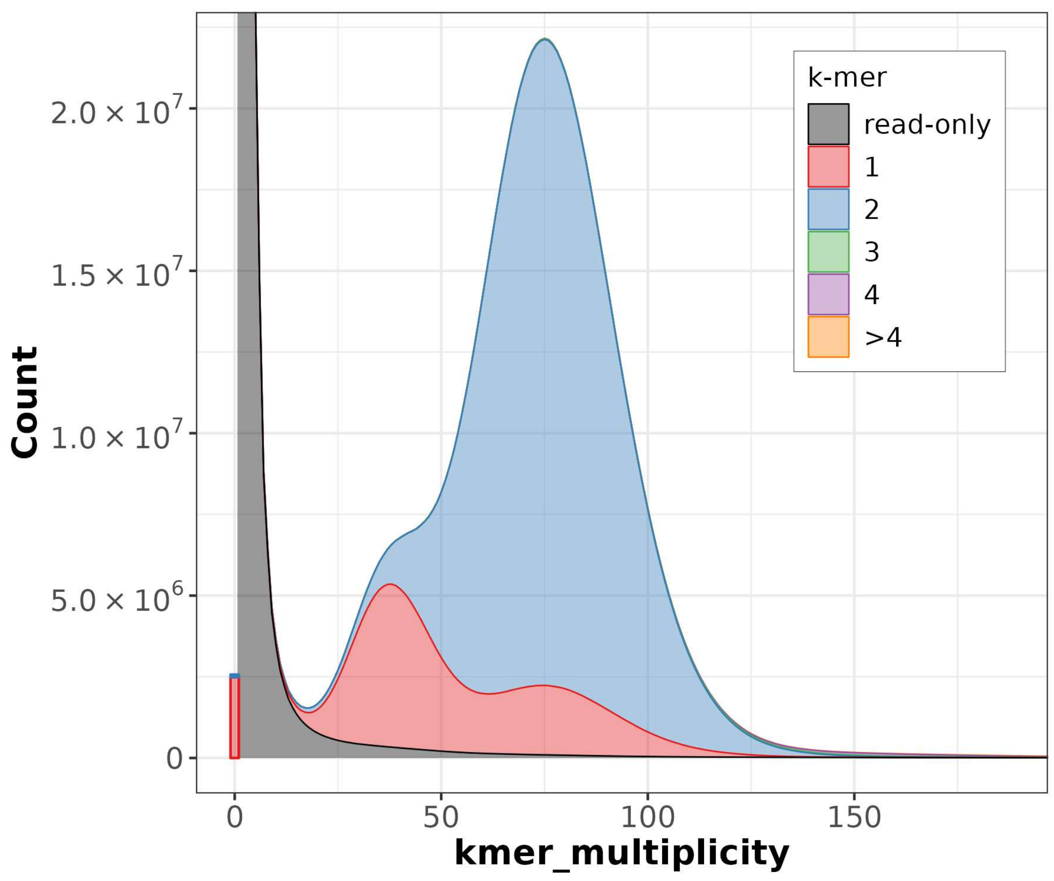

Assembly ID: corvus_macrorhynchos

Database built with kmer size: 21 bp

Number of haploptypes/assemblies: 1

Expected genome size: 1073516218

Heterozygosity: 0.4 %

QV Score

assembly_name	Assembly_Only	Total_Kmers	QV_Score
corvus_macrorhynchos.asm1	226750	1013793606	49.7258
ALT_missing	0	0	nan
Both	226750	1013793606	49.7258

Kmer Completeness

assembly_name	Kmers_Assembly	Kmers_Reads	%
corvus_macrorhynchos.asm1	988452825	1064348938	92.8692
ALT_missing	0	1064348938	0
both	988452825	1064348938	92.8692

BUSCOv5 (database: passeriformes)

Metric	asm1 #	asm1 %
Complete	9541	88.0
Complete and single-copy	9517	87.8
Complete and duplicated	24	0.2
Fragmented	348	3.2
Missing	955	8.8
Total searched groups	10844	100.0

Genomescope2 Profile (Linear)

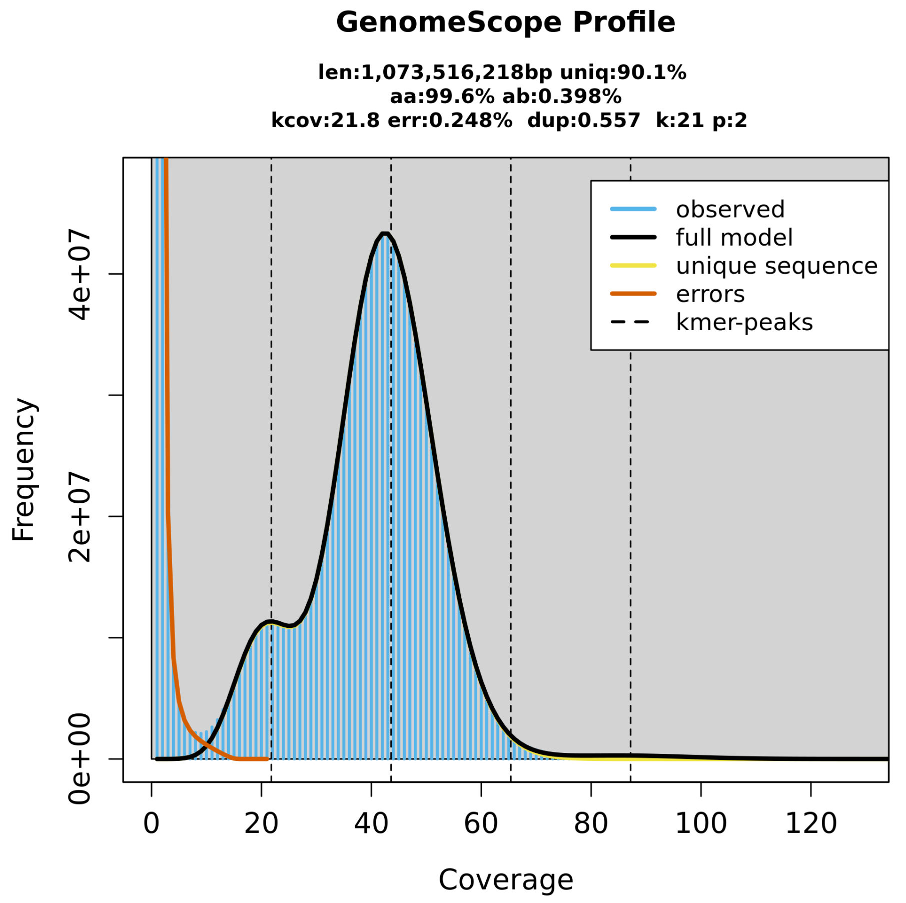

Genomescope2 Profile (Log)

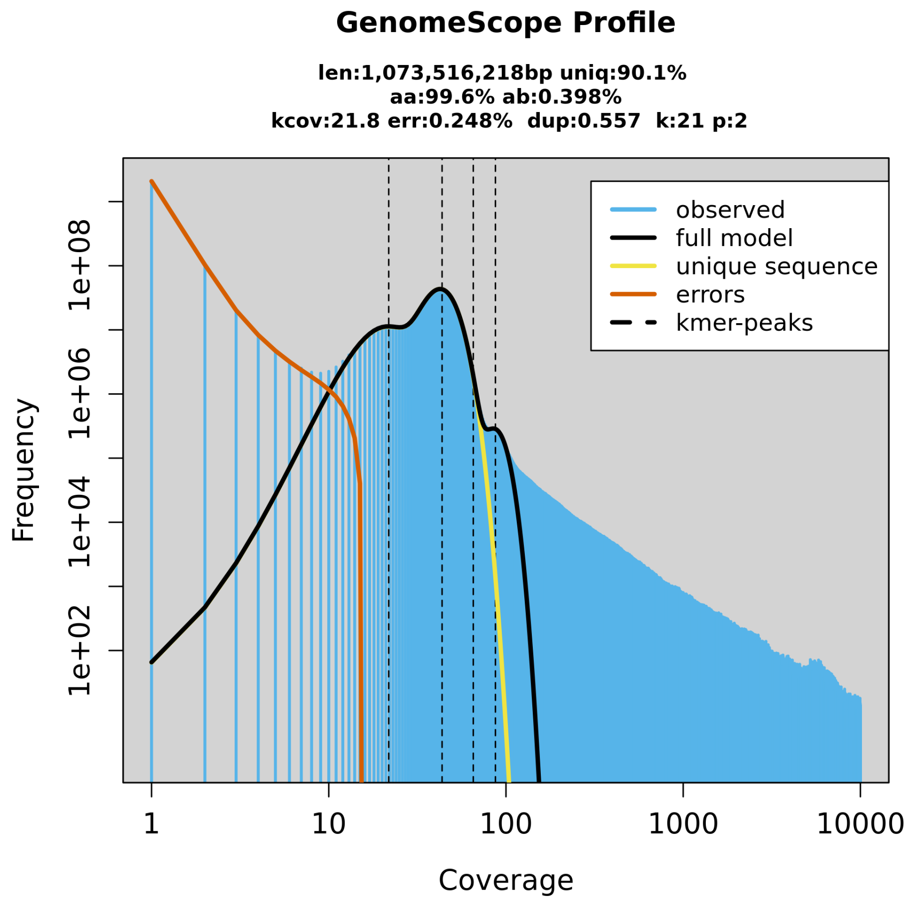

Genome Statistics with gfastats

Metric	asm1
Total scaffold length	1018435877
GC content %	42.01
# gaps in scaffolds	35779
Total gap length in scaffolds	3596231
# scaffolds	16523
Largest scaffold	3346143
Scaffold N50	133802
Scaffold L50	2023
Scaffold L90	8512
# contigs	52302
Largest contig	413299
Contig N50	49133
Contig L50	5606
Contig L90	22902

K-mer Multiplicity asm1 Only (Stacked)

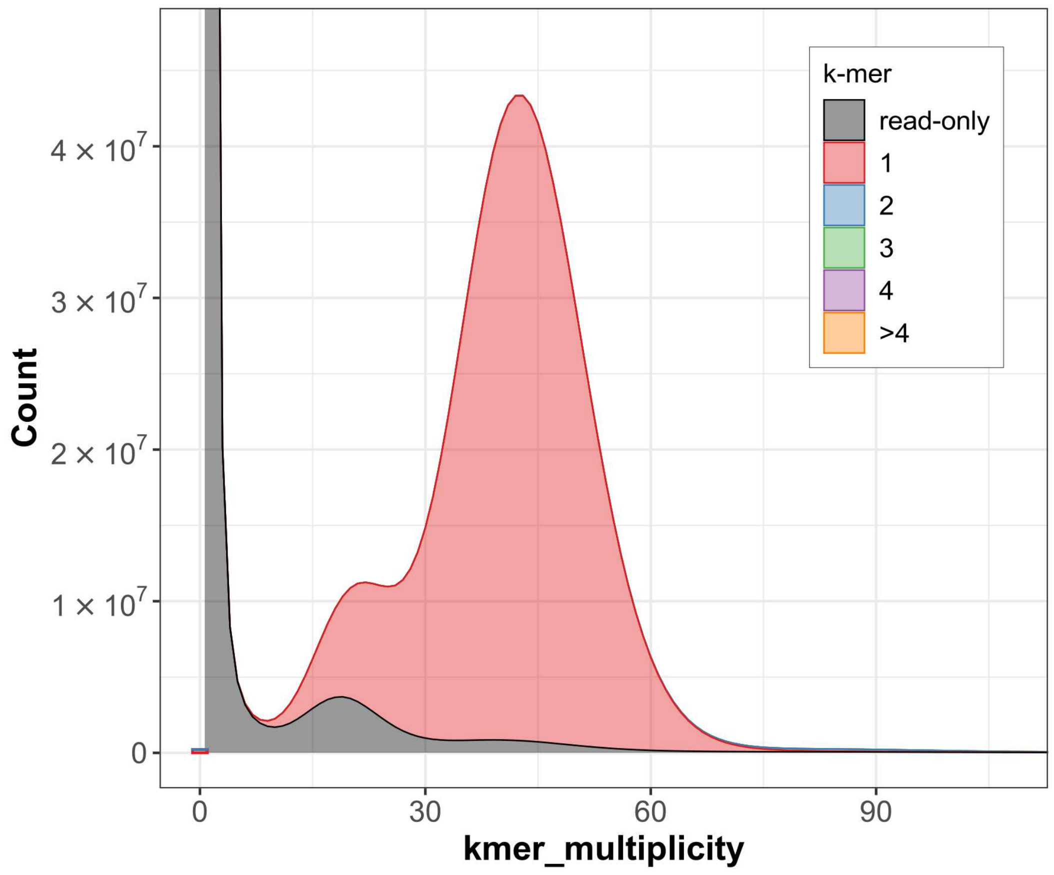

Assembly ID: corvus_kubaryi

Database built with kmer size: 21 bp

Number of haploptypes/assemblies: 2

Expected genome size: 1113063796

Heterozygosity: 0.01 %

QV Score

assembly_name	Assembly_Only	Total_Kmers	QV_Score
corvus_kubaryi.asm1	94708	1046014626	53.6535
corvus_kubaryi.asm2	95056	1056616574	53.6814
Both	189764	2102631200	53.6675

Kmer Completeness

assembly_name	Kmers_Assembly	Kmers_Reads	%
corvus_kubaryi.asm1	977904232	1023324505	95.5615
corvus_kubaryi.asm2	991909837	1023324505	96.9301
both	992048214	1023324505	96.9437

BUSCOv5 (database: passeriformes)

Metric	asm1 #	asm1 %	asm2 #	asm2 %
Complete	10221	94.2	10333	95.3
Complete and single-copy	9869	91.0	10039	92.6
Complete and duplicated	352	3.2	294	2.7
Fragmented	76	0.7	76	0.7
Missing	547	5.1	435	4.0
Total searched groups	10844	100.0	10844	100.0

Genomescope2 Profile (Linear)

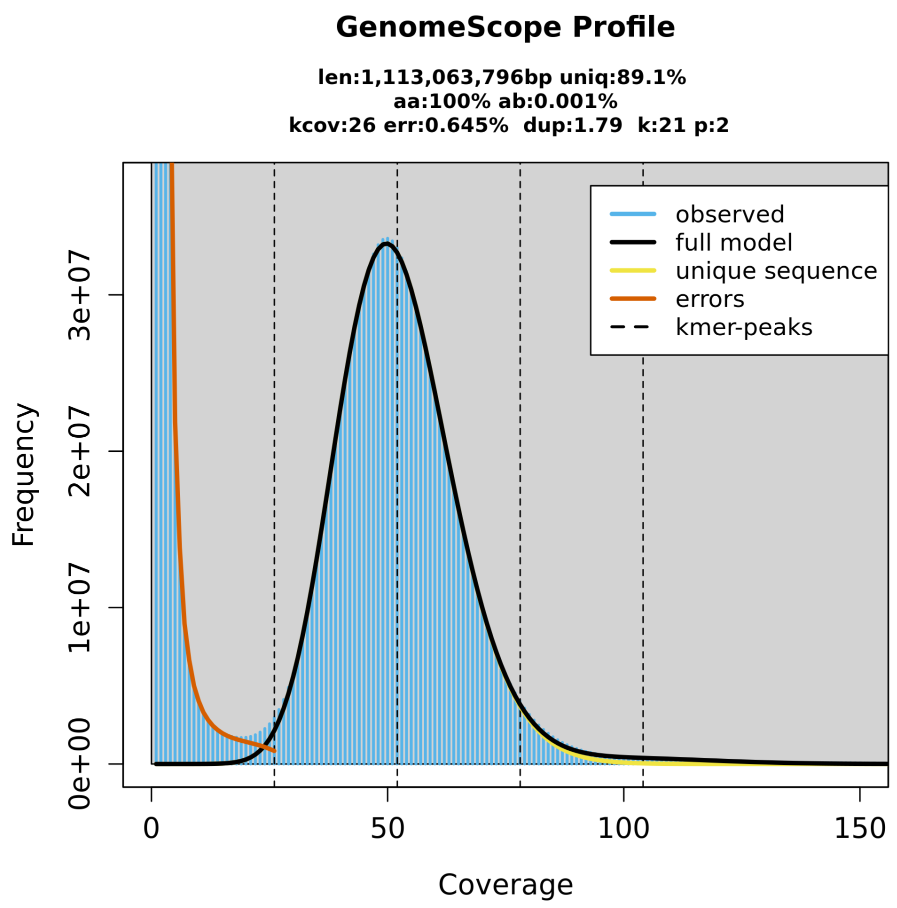

Genomescope2 Profile (Log)

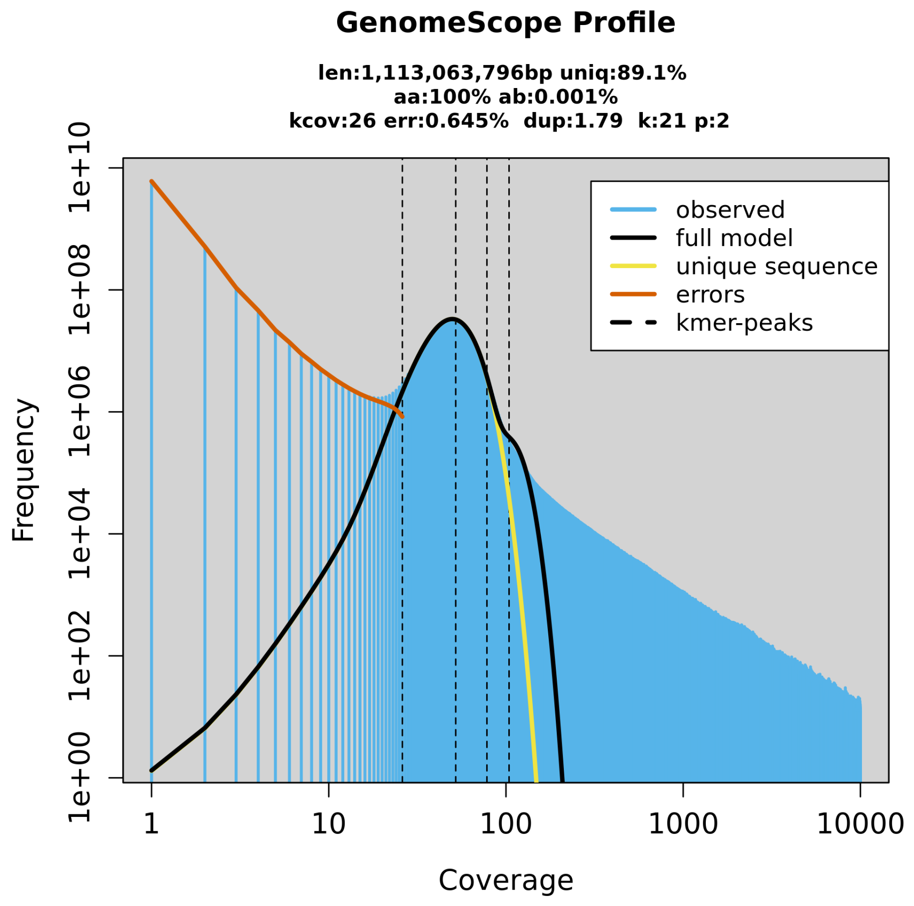

Genome Statistics with gfastats

Metric	asm1	asm2
Total scaffold length	1057484666	1068123824
GC content %	42.14	42.10
# gaps in scaffolds	10579	10675
Total gap length in scaffolds	11229600	11264890
# scaffolds	1443	1443
Largest scaffold	57584386	57584386
Scaffold N50	17891236	17097965
Scaffold L50	18	19
Scaffold L90	82	82
# contigs	12022	12118
Largest contig	1366860	1366860
Contig N50	211131	211228
Contig L50	1437	1453
Contig L90	5350	5400

K-mer Multiplicity asm1 Only (Stacked)

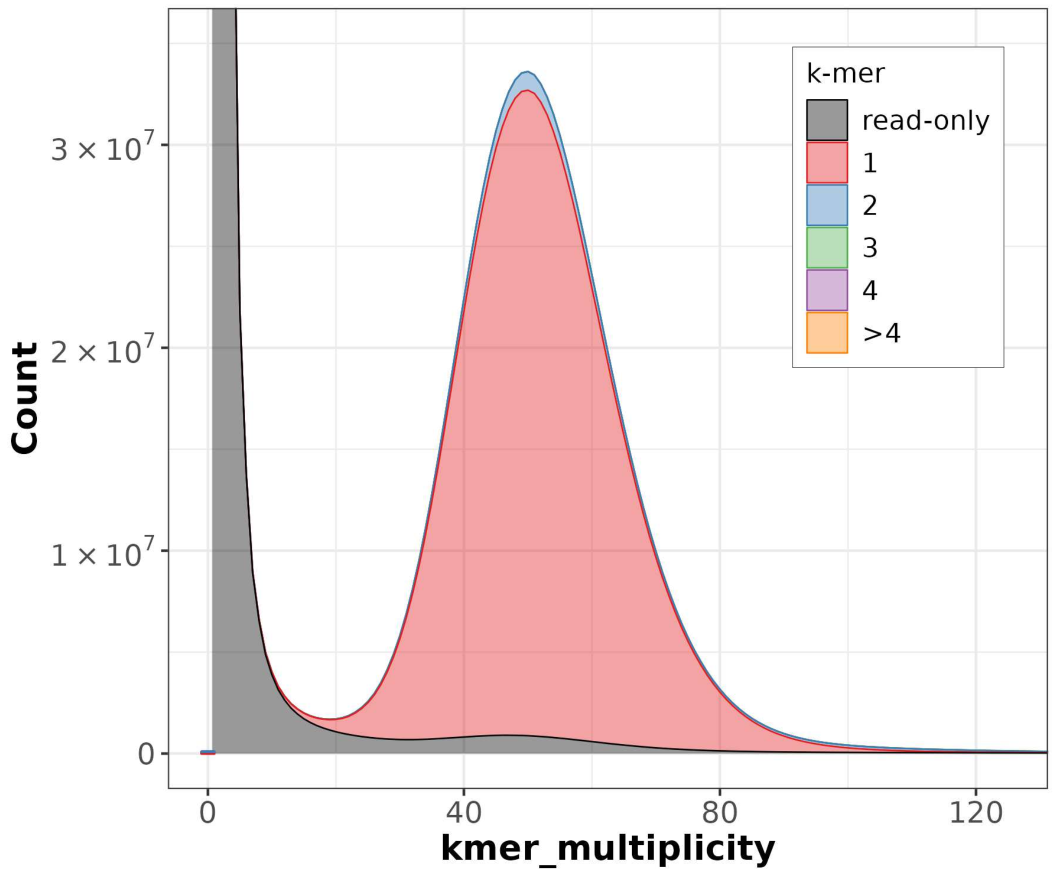

K-mer Multiplicity asm1 + asm2 (Stacked)

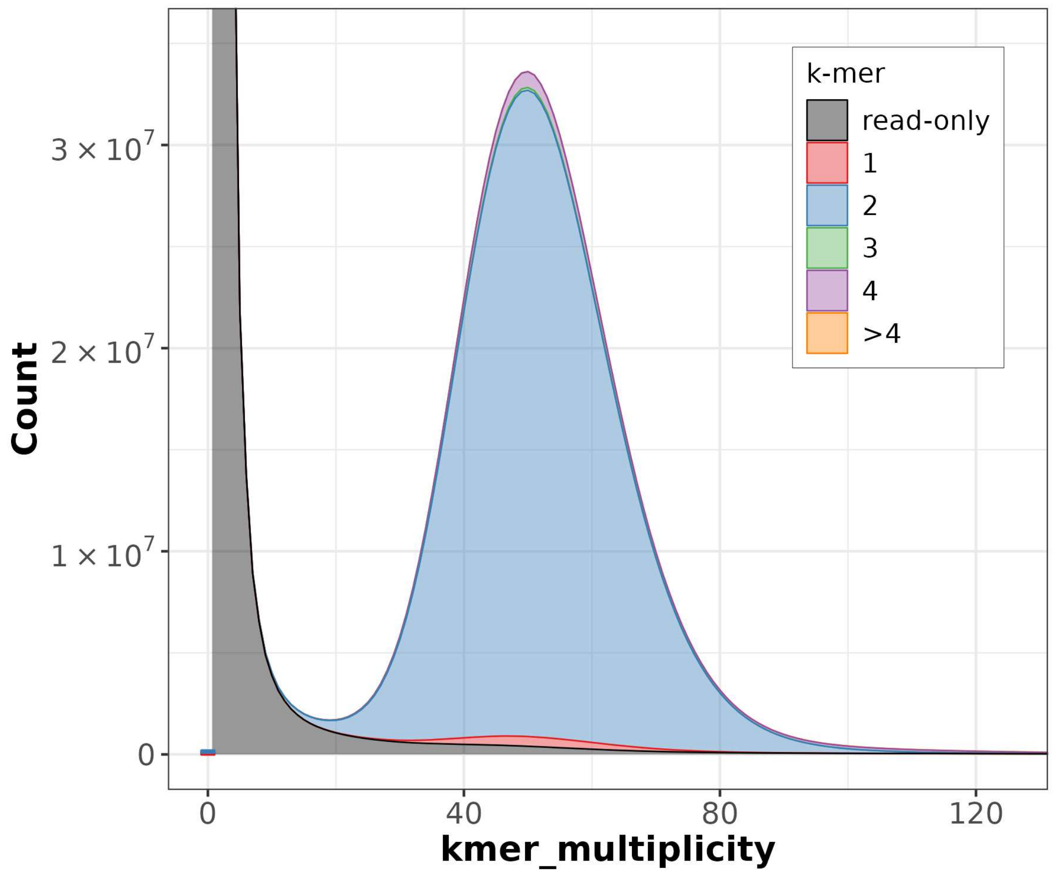

Assembly ID: corvus_hawaiiensis

Database built with kmer size: 31 bp

Number of haploptypes/assemblies: 2

Expected genome size: 456950664

Heterozygosity: 100.0 %

QV Score

assembly_name	Assembly_Only	Total_Kmers	QV_Score
corvus_hawaiiensis.asm1	1381549	1149771634	42.4222
corvus_hawaiiensis.asm2	225718	628789229	47.6708
Both	1607267	1778560863	43.6601

Kmer Completeness

assembly_name	Kmers_Assembly	Kmers_Reads	%
corvus_hawaiiensis.asm1	990227407	1008729096	98.1658
corvus_hawaiiensis.asm2	561338518	1008729096	55.6481
both	999798627	1008729096	99.1147

BUSCOv5 (database: passeriformes)

Metric	asm1 #	asm1 %	asm2 #	asm2 %
Complete	10489	96.8	5925	54.6
Complete and single-copy	10460	96.5	5899	54.4
Complete and duplicated	29	0.3	26	0.2
Fragmented	53	0.5	102	0.9
Missing	302	2.7	4817	44.5
Total searched groups	10844	100.0	10844	100.0

Genomescope2 Profile (Linear)

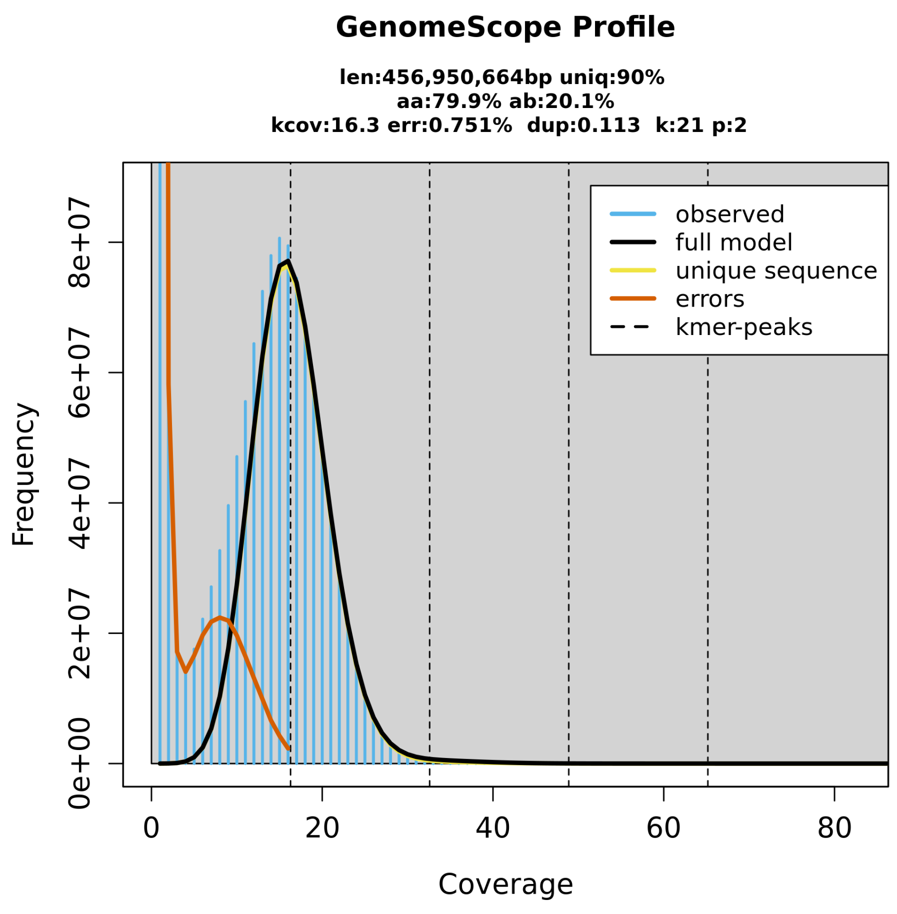

Genomescope2 Profile (Log)

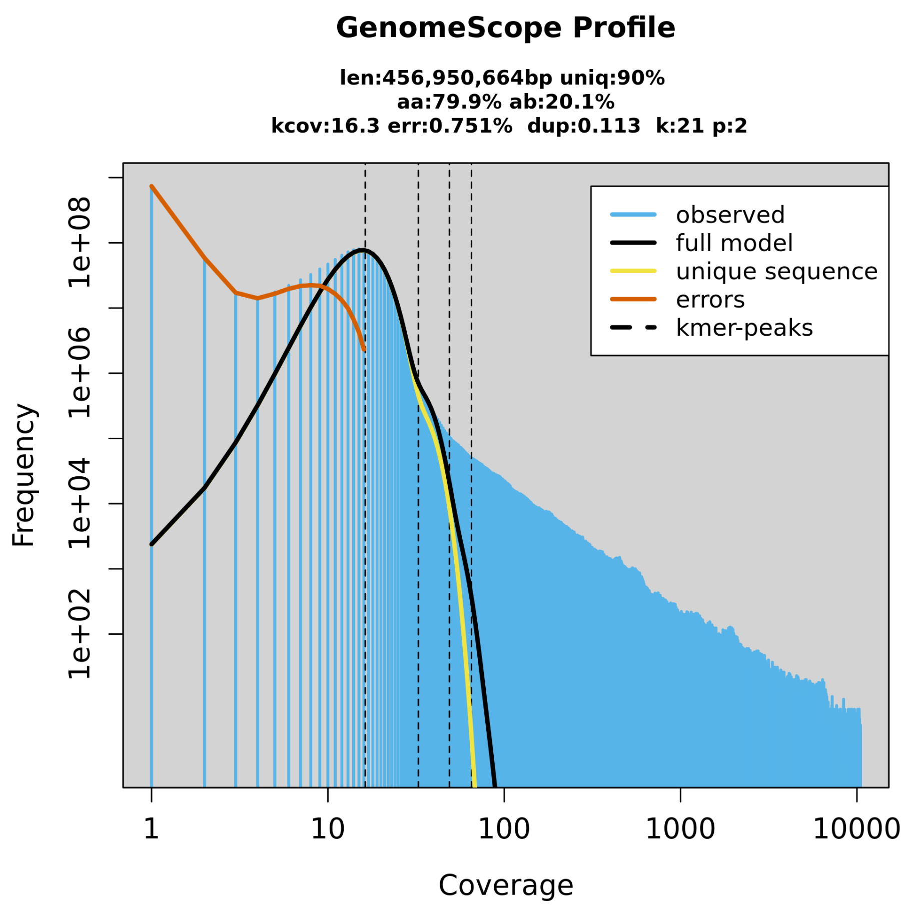

Genome Statistics with gfastats

Metric	asm1	asm2
Total scaffold length	1151594481	628844469
GC content %	43.30	43.16
# gaps in scaffolds	294	0
Total gap length in scaffolds	1813227	0
# scaffolds	187	2762
Largest scaffold	123451405	4019362
Scaffold N50	76278832	584432
Scaffold L50	6	299
Scaffold L90	23	1311
# contigs	481	2762
Largest contig	80326523	4019362
Contig N50	23064755	584432
Contig L50	15	299
Contig L90	72	1311

K-mer Multiplicity asm1 Only (Stacked)

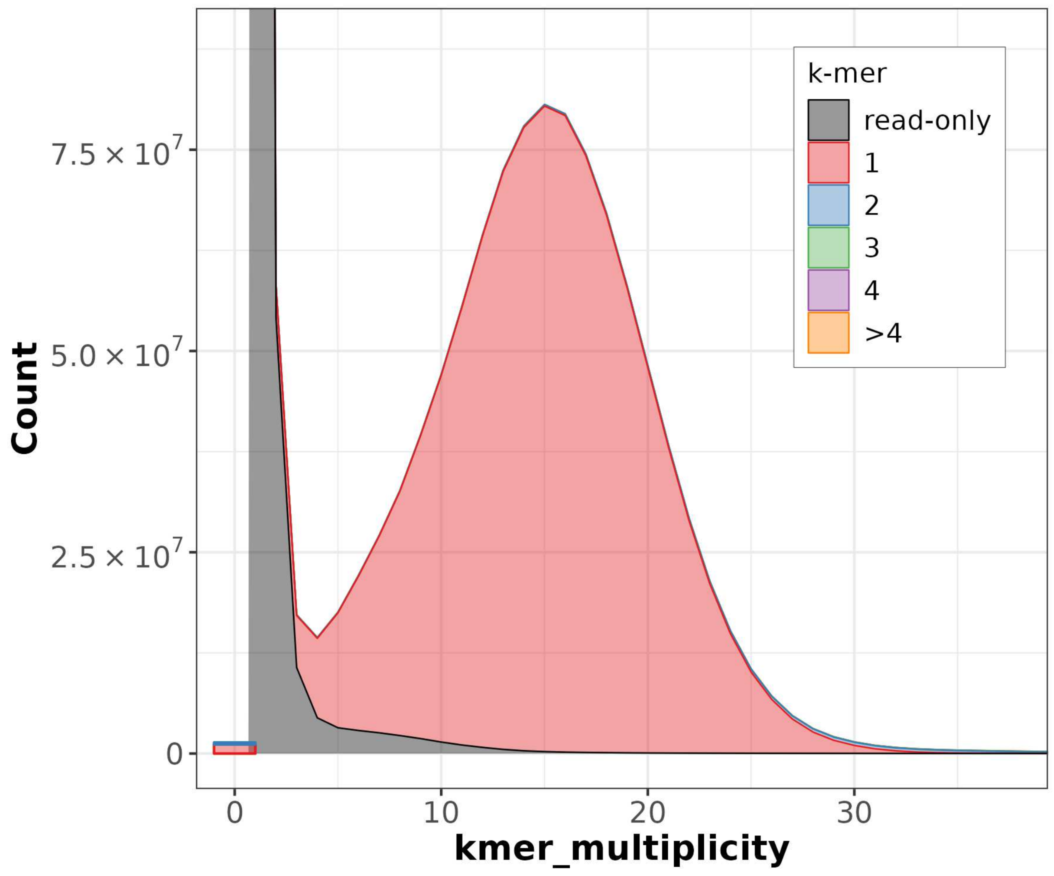

K-mer Multiplicity asm1 + asm2 (Stacked)

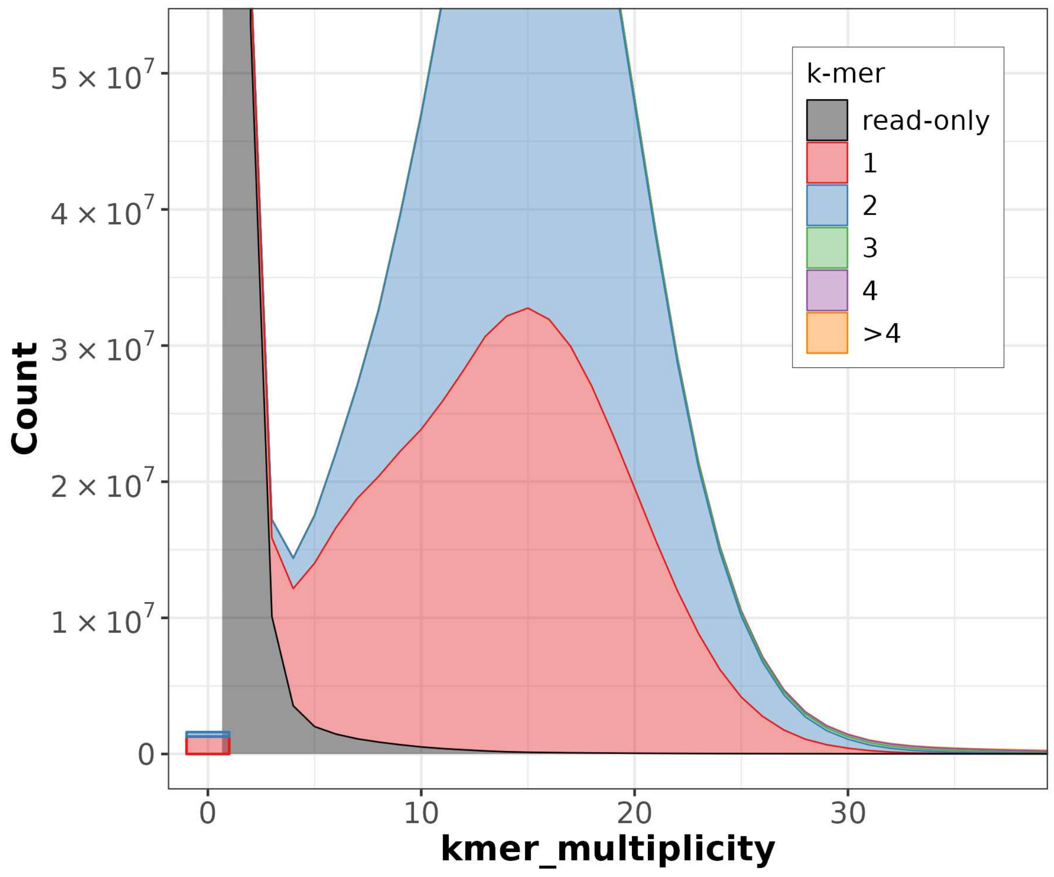

Assembly ID: corvus_brachyrhynchos

Database built with kmer size: 21 bp

Number of haploptypes/assemblies: 1

Expected genome size: 967936602

Heterozygosity: 0.778 %

QV Score

assembly_name	Assembly_Only	Total_Kmers	QV_Score
corvus_brachyrhynchos.asm1	3923690	1049988941	37.4894
ALT_missing	0	0	nan
Both	3923690	1049988941	37.4894

Kmer Completeness

assembly_name	Kmers_Assembly	Kmers_Reads	%
corvus_brachyrhynchos.asm1	991553734	1044521904	94.929
ALT_missing	0	1044521904	0
both	991553734	1044521904	94.929

BUSCOv5 (database: passeriformes)

Metric	asm1 #	asm1 %
Complete	10397	95.9
Complete and single-copy	10363	95.6
Complete and duplicated	34	0.3
Fragmented	105	1.0
Missing	342	3.1
Total searched groups	10844	100.0

Genomescope2 Profile (Linear)

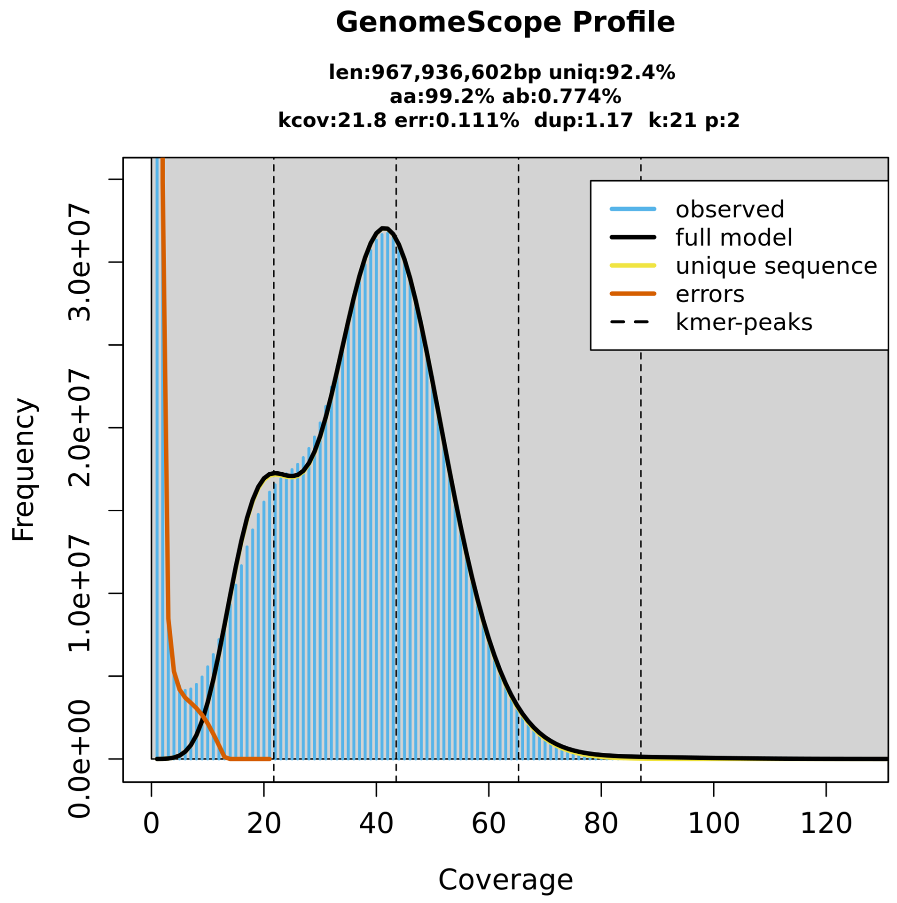

Genomescope2 Profile (Log)

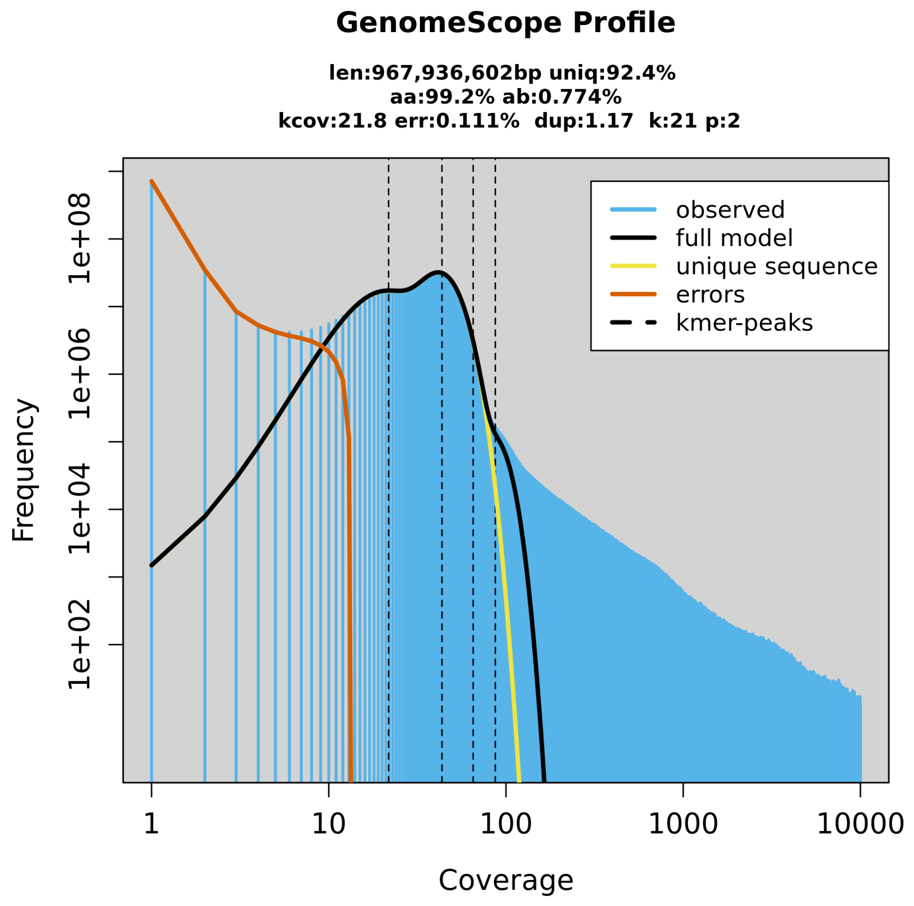

Genome Statistics with gfastats

Metric	asm1
Total scaffold length	1091312783
GC content %	41.85
# gaps in scaffolds	79099
Total gap length in scaffolds	39530922
# scaffolds	10547
Largest scaffold	25919742
Scaffold N50	6953989
Scaffold L50	49
Scaffold L90	195
# contigs	89646
Largest contig	264908
Contig N50	29093
Contig L50	10509
Contig L90	38490

K-mer Multiplicity asm1 Only (Stacked)

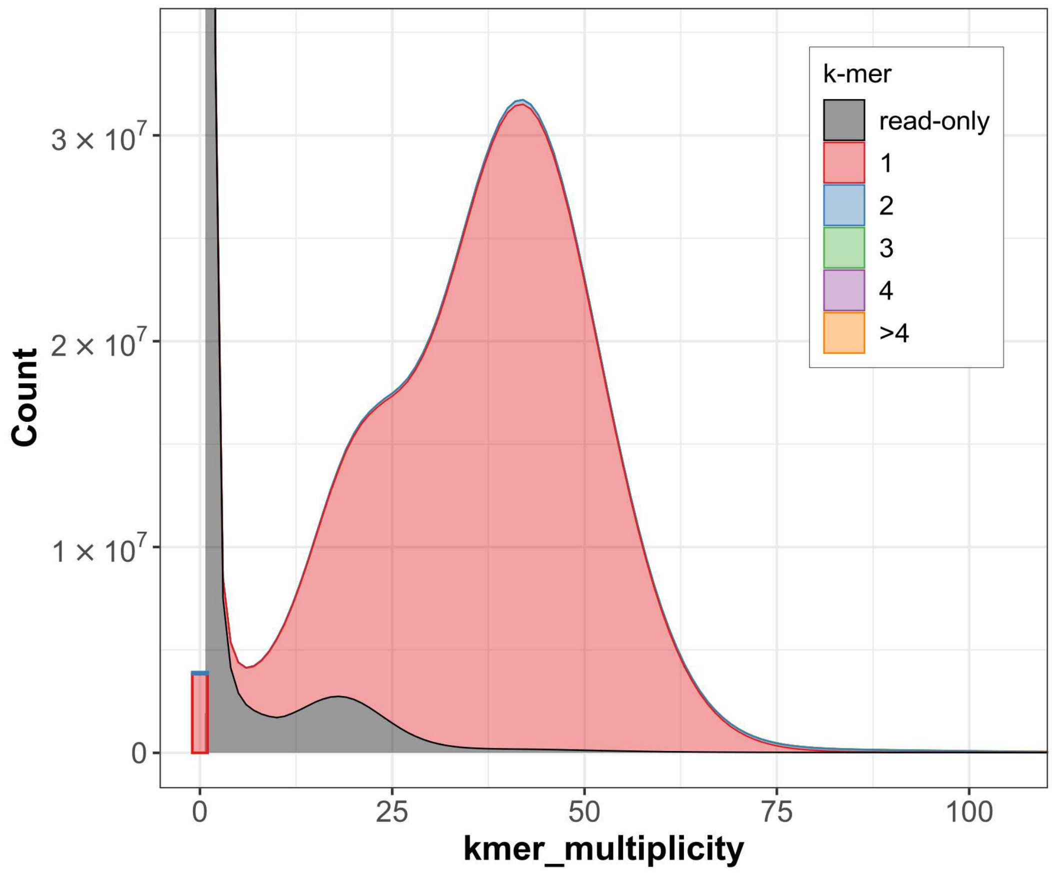

Assembly ID: cnemophilus_loriae

Database built with kmer size: 21 bp

Number of haploptypes/assemblies: 1

Expected genome size: 1015050288

Heterozygosity: 0.876 %

QV Score

assembly_name	Assembly_Only	Total_Kmers	QV_Score
cnemophilus_loriae.asm1	1094158	1034293968	42.9756
ALT_missing	0	0	nan
Both	1094158	1034293968	42.9756

Kmer Completeness

assembly_name	Kmers_Assembly	Kmers_Reads	%
cnemophilus_loriae.asm1	996675583	1093442004	91.1503
ALT_missing	0	1093442004	0
both	996675583	1093442004	91.1503

BUSCOv5 (database: passeriformes)

Metric	asm1 #	asm1 %
Complete	10391	95.8
Complete and single-copy	10361	95.5
Complete and duplicated	30	0.3
Fragmented	80	0.7
Missing	373	3.5
Total searched groups	10844	100.0

Genomescope2 Profile (Linear)

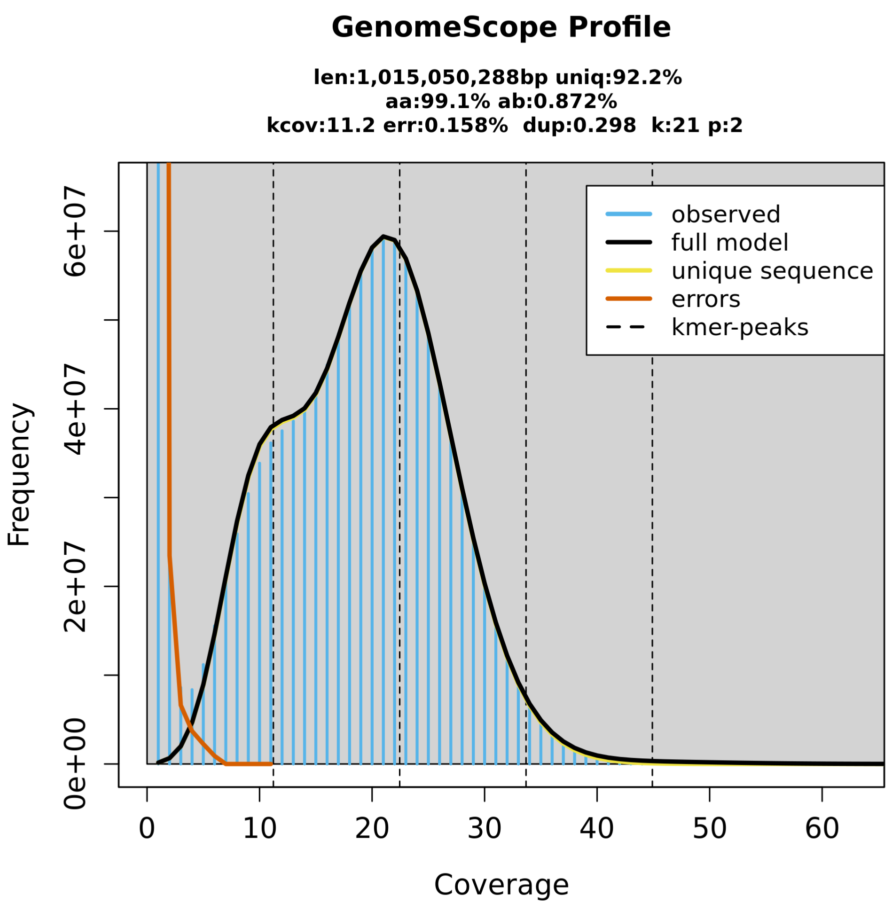

Genomescope2 Profile (Log)

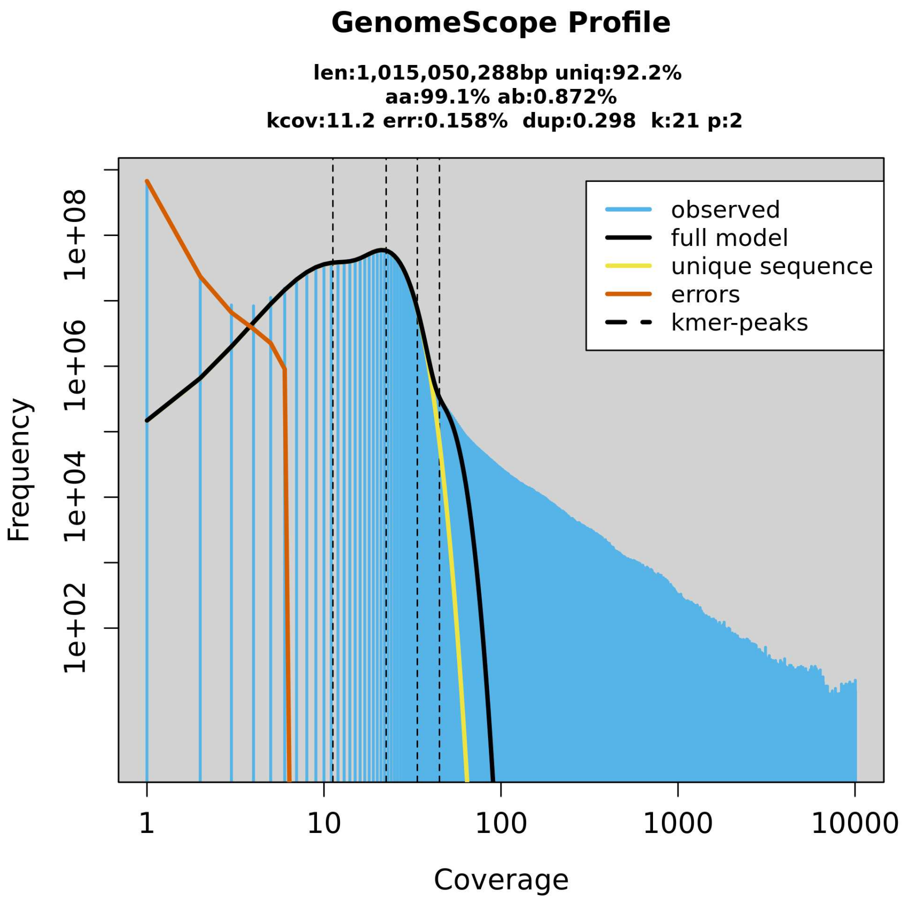

Genome Statistics with gfastats

Metric	asm1
Total scaffold length	1043492295
GC content %	42.25
# gaps in scaffolds	52732
Total gap length in scaffolds	7793787
# scaffolds	17495
Largest scaffold	7605640
Scaffold N50	1426893
Scaffold L50	200
Scaffold L90	867
# contigs	70227
Largest contig	499561
Contig N50	67183
Contig L50	4330
Contig L90	17021

K-mer Multiplicity asm1 Only (Stacked)

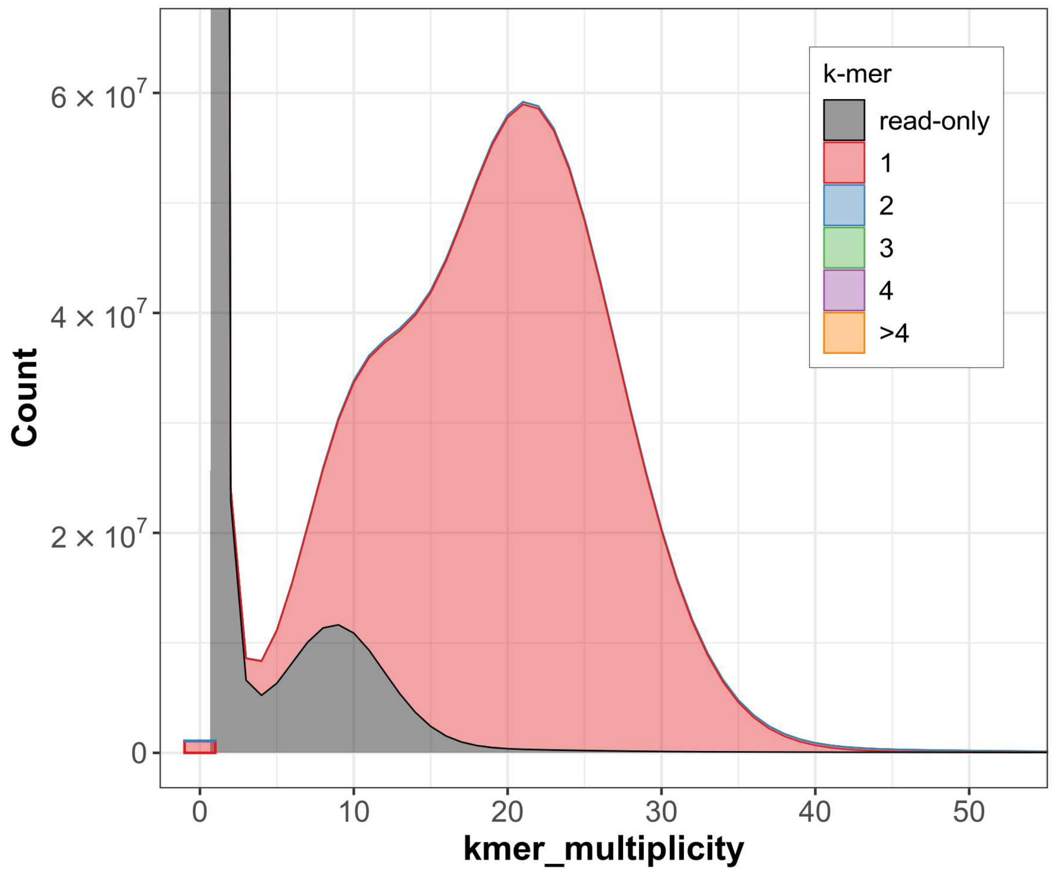

Assembly ID: aphelocoma_coerulescens

Database built with kmer size: 21 bp

Number of haploptypes/assemblies: 1

Expected genome size: 849419954

Heterozygosity: 5.544 %

QV Score

assembly_name	Assembly_Only	Total_Kmers	QV_Score
aphelocoma_coerulescens.asm1	436046	1041083219	47.0009
ALT_missing	0	0	nan
Both	436046	1041083219	47.0009

Kmer Completeness

assembly_name	Kmers_Assembly	Kmers_Reads	%
aphelocoma_coerulescens.asm1	1002456092	1059991445	94.5721
ALT_missing	0	1059991445	0
both	1002456092	1059991445	94.5721

BUSCOv5 (database: passeriformes)

Metric	asm1 #	asm1 %
Complete	10456	96.4
Complete and single-copy	10437	96.2
Complete and duplicated	19	0.2
Fragmented	66	0.6
Missing	322	3.0
Total searched groups	10844	100.0

Genomescope2 Profile (Linear)

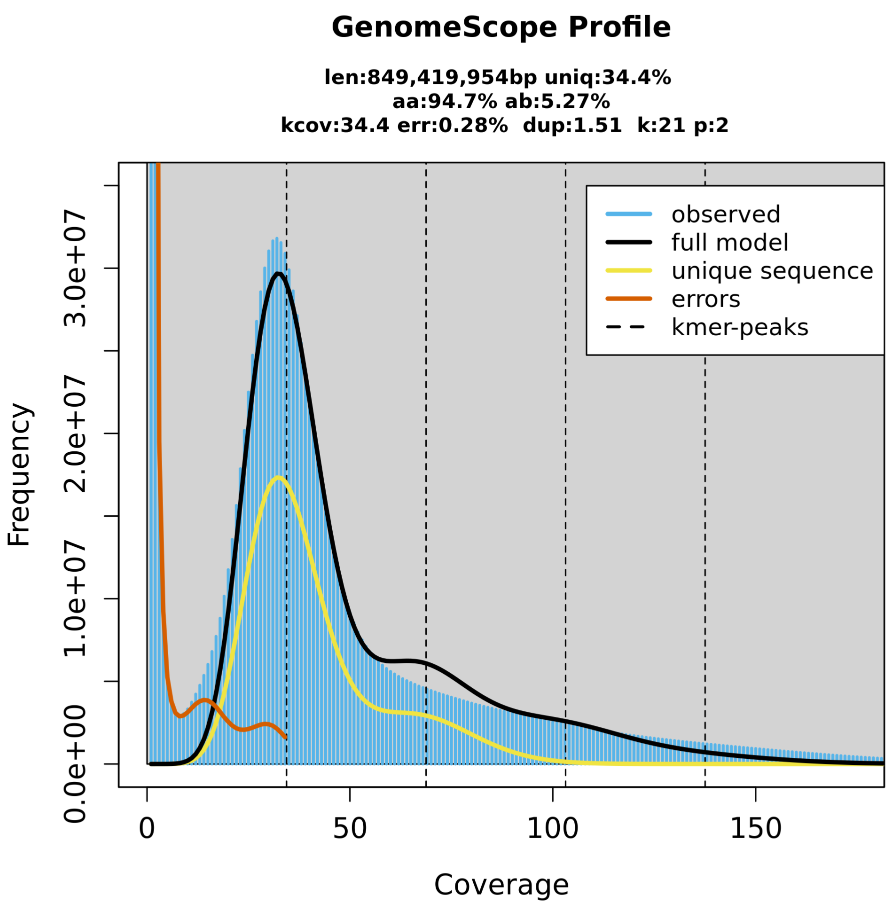

Genomescope2 Profile (Log)

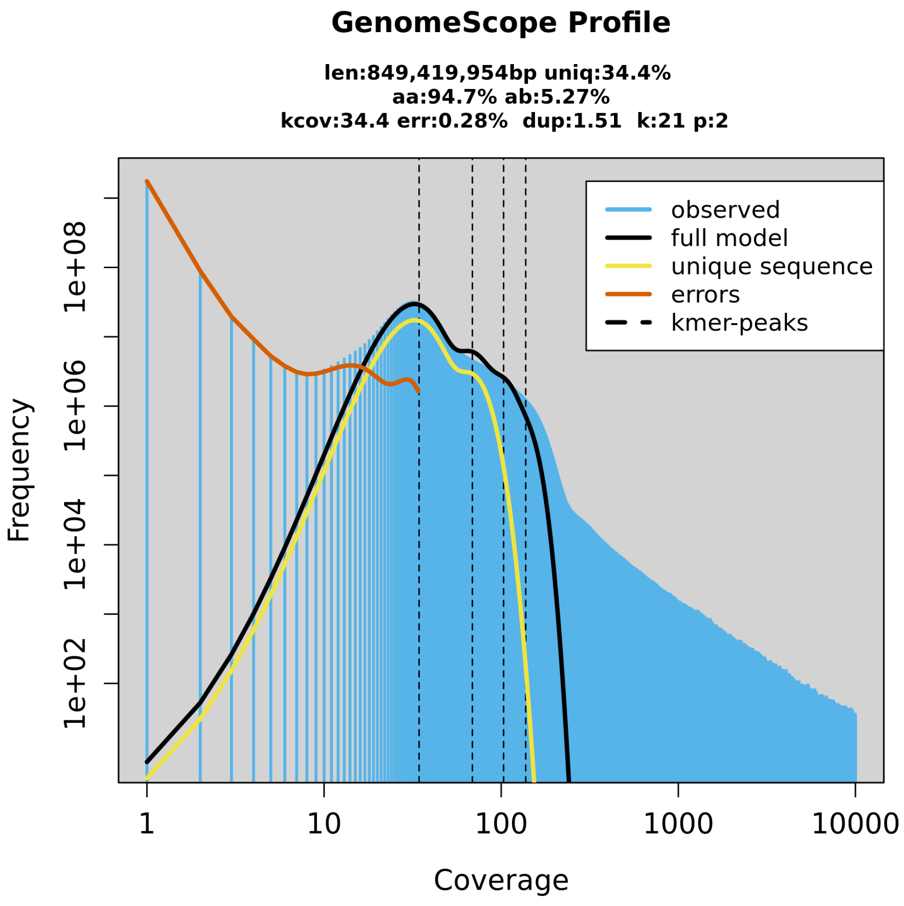

Genome Statistics with gfastats

Metric	asm1
Total scaffold length	1060855343
GC content %	42.08
# gaps in scaffolds	22509
Total gap length in scaffolds	19287934
# scaffolds	1701
Largest scaffold	29167858
Scaffold N50	7634200
Scaffold L50	42
Scaffold L90	170
# contigs	24210
Largest contig	731130
Contig N50	118847
Contig L50	2548
Contig L90	9456

K-mer Multiplicity asm1 Only (Stacked)

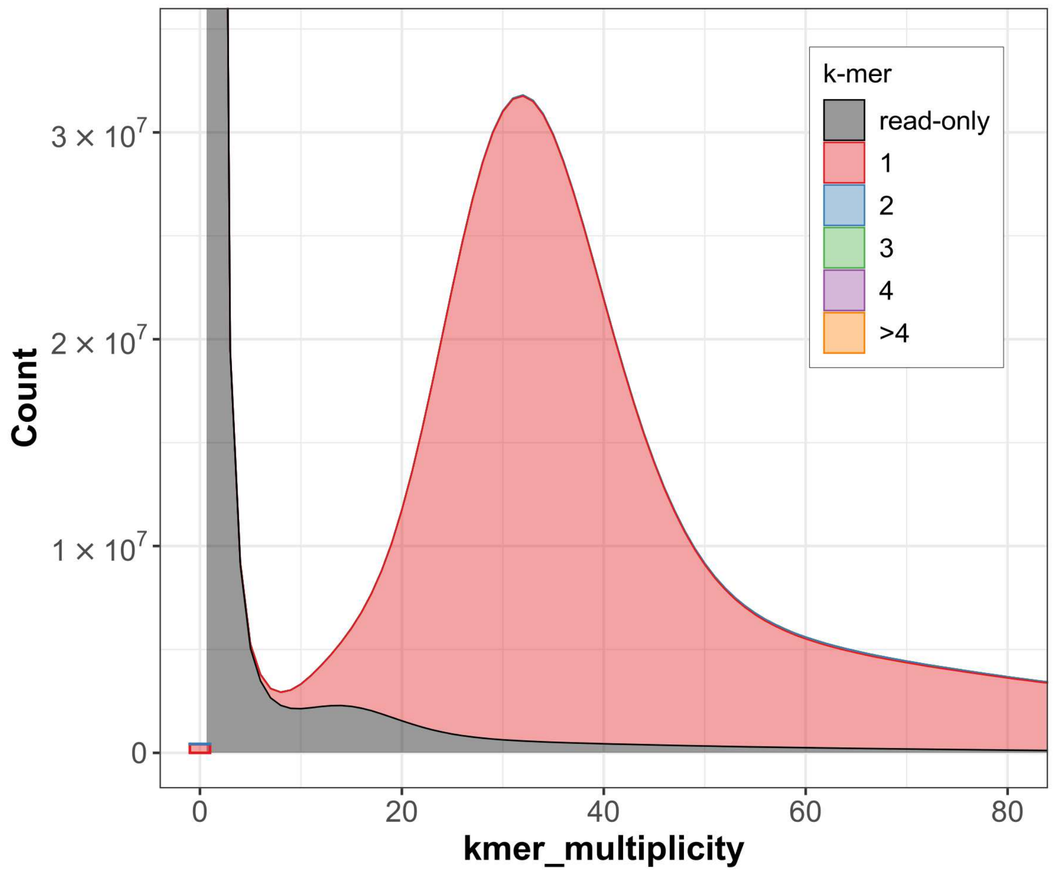

Assembly ID: aphelocoma_californica

Database built with kmer size: 31 bp

Number of haploptypes/assemblies: 2

Expected genome size: 1074075332

Heterozygosity: 0.664 %

QV Score

assembly_name	Assembly_Only	Total_Kmers	QV_Score
aphelocoma_californica.asm1	13095	1348439925	63.3494
aphelocoma_californica.asm2	10683	1191226535	63.6952
Both	23778	2539666460	63.5082

Kmer Completeness

assembly_name	Kmers_Assembly	Kmers_Reads	%
aphelocoma_californica.asm1	1023672124	1108168477	92.3751
aphelocoma_californica.asm2	949302914	1108168477	85.6641
both	1103328527	1108168477	99.5632

BUSCOv5 (database: passeriformes)

Metric	asm1 #	asm1 %	asm2 #	asm2 %
Complete	10454	96.4	9995	92.2
Complete and single-copy	10403	95.9	9966	91.9
Complete and duplicated	51	0.5	29	0.3
Fragmented	59	0.5	62	0.6
Missing	331	3.1	787	7.2
Total searched groups	10844	100.0	10844	100.0

Genomescope2 Profile (Linear)

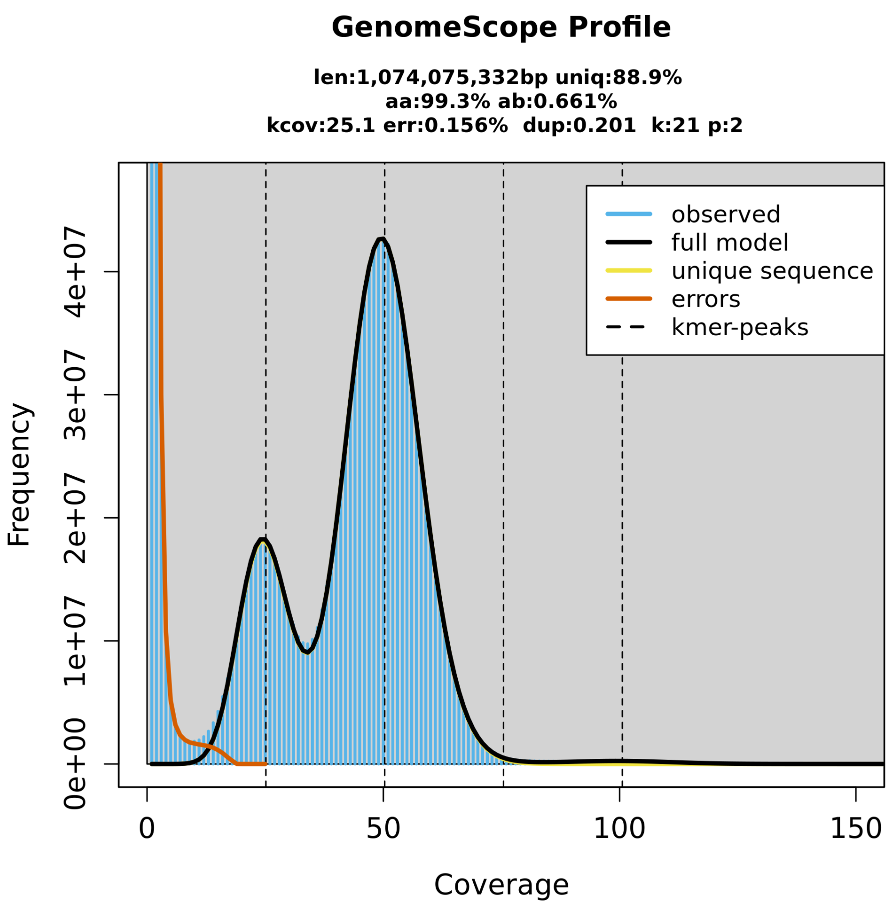

Genomescope2 Profile (Log)

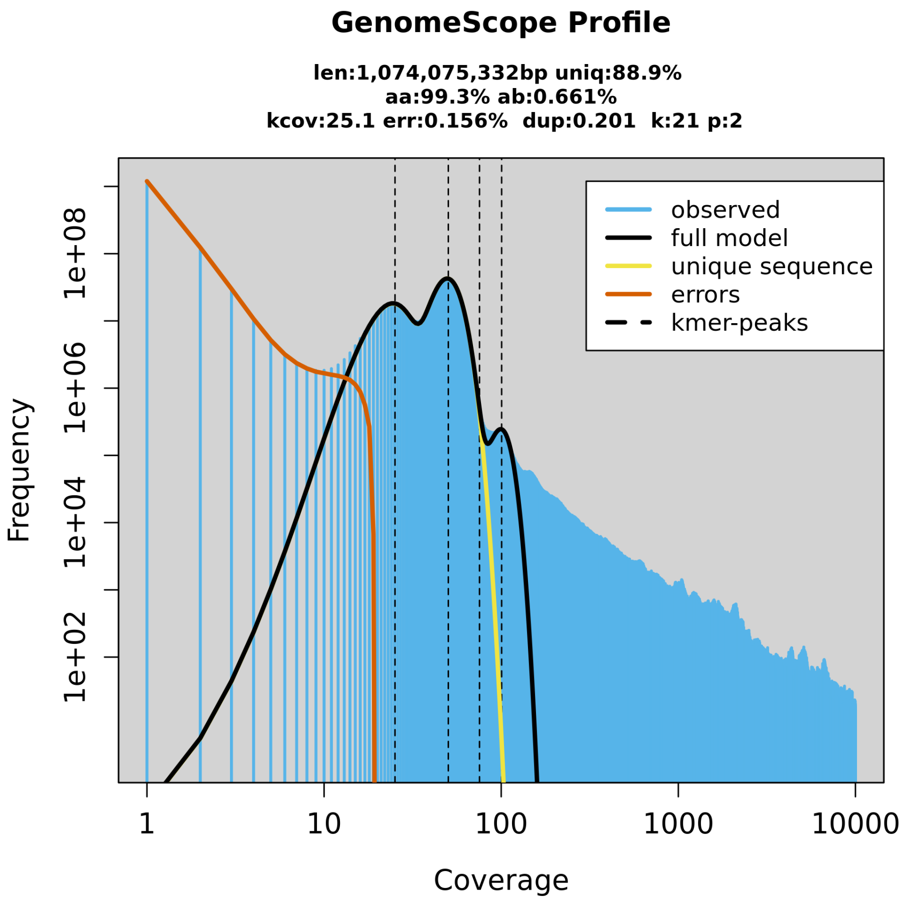

Genome Statistics with gfastats

Metric	asm1	asm2
Total scaffold length	1348500425	1191279315
GC content %	44.58	44.29
# gaps in scaffolds	272	168
Total gap length in scaffolds	27200	16800
# scaffolds	1393	1631
Largest scaffold	158410743	160920769
Scaffold N50	66144195	65728546
Scaffold L50	7	6
Scaffold L90	115	92
# contigs	1665	1799
Largest contig	77933725	52308079
Contig N50	11534525	11670509
Contig L50	28	27
Contig L90	245	218

K-mer Multiplicity asm1 Only (Stacked)

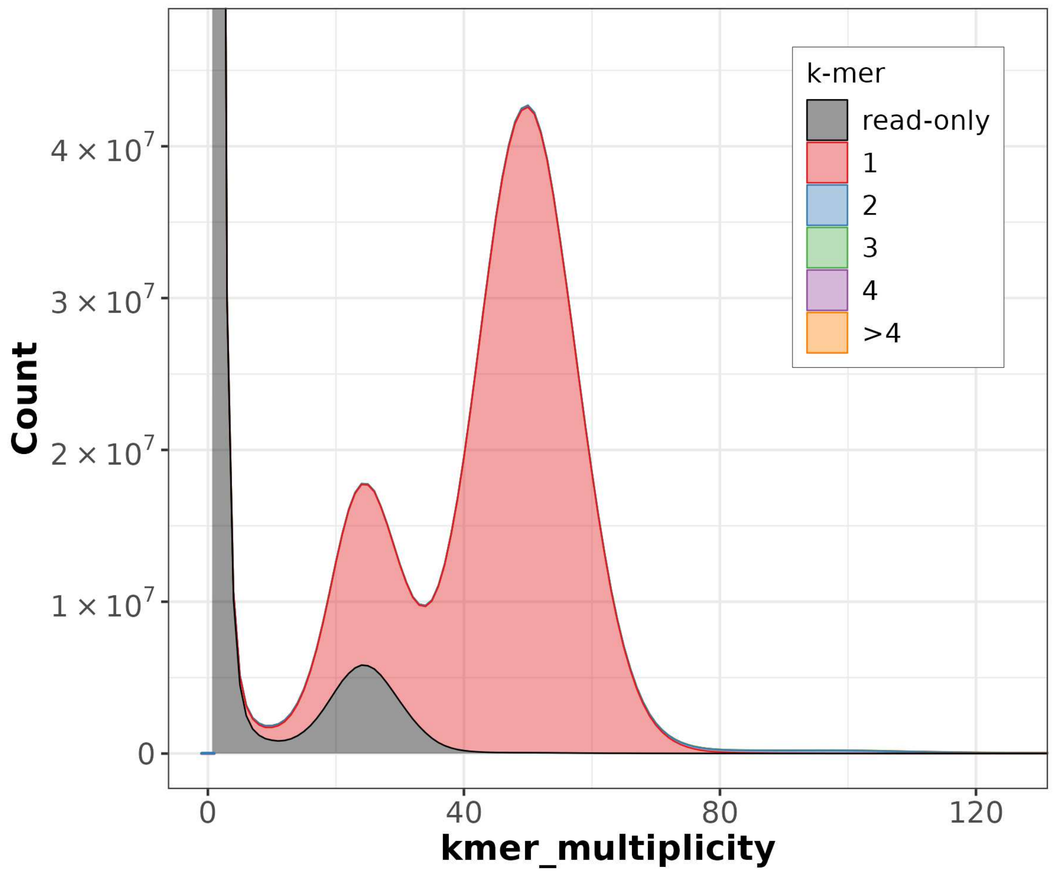

K-mer Multiplicity asm1 + asm2 (Stacked)

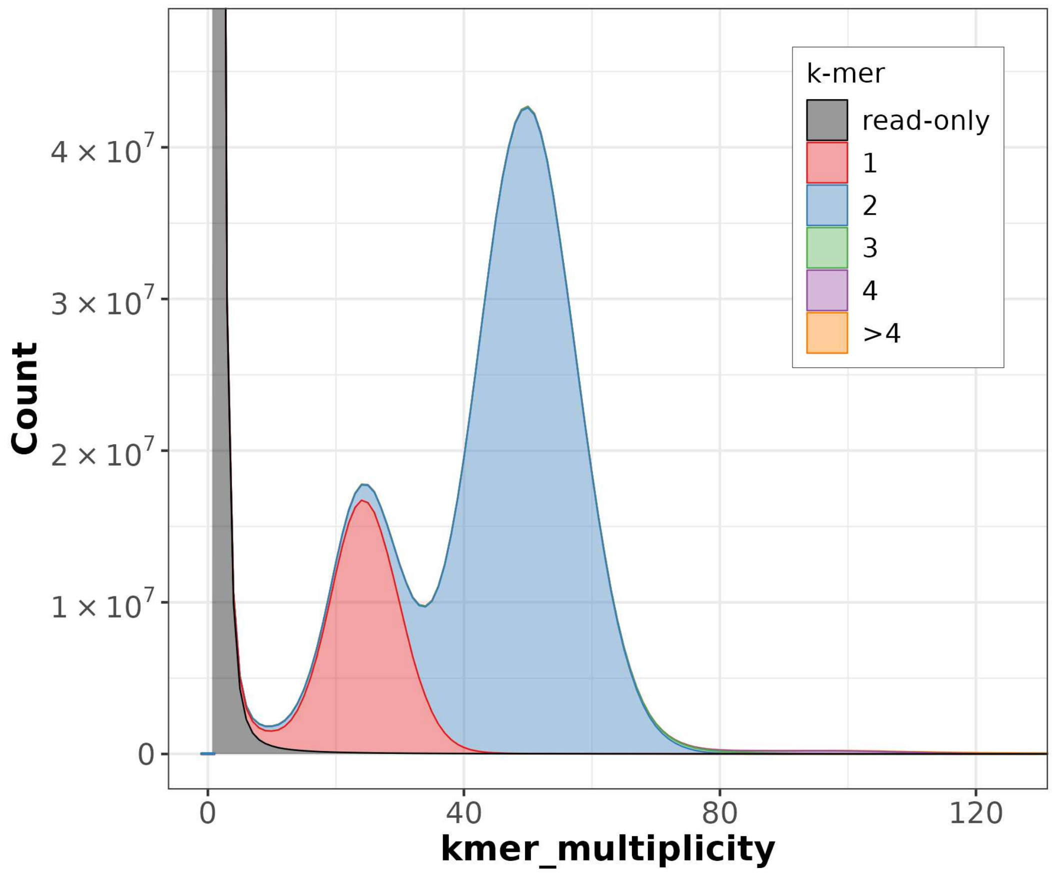

cyanocitta__stelleri HiC Heatmap (assembly 1)

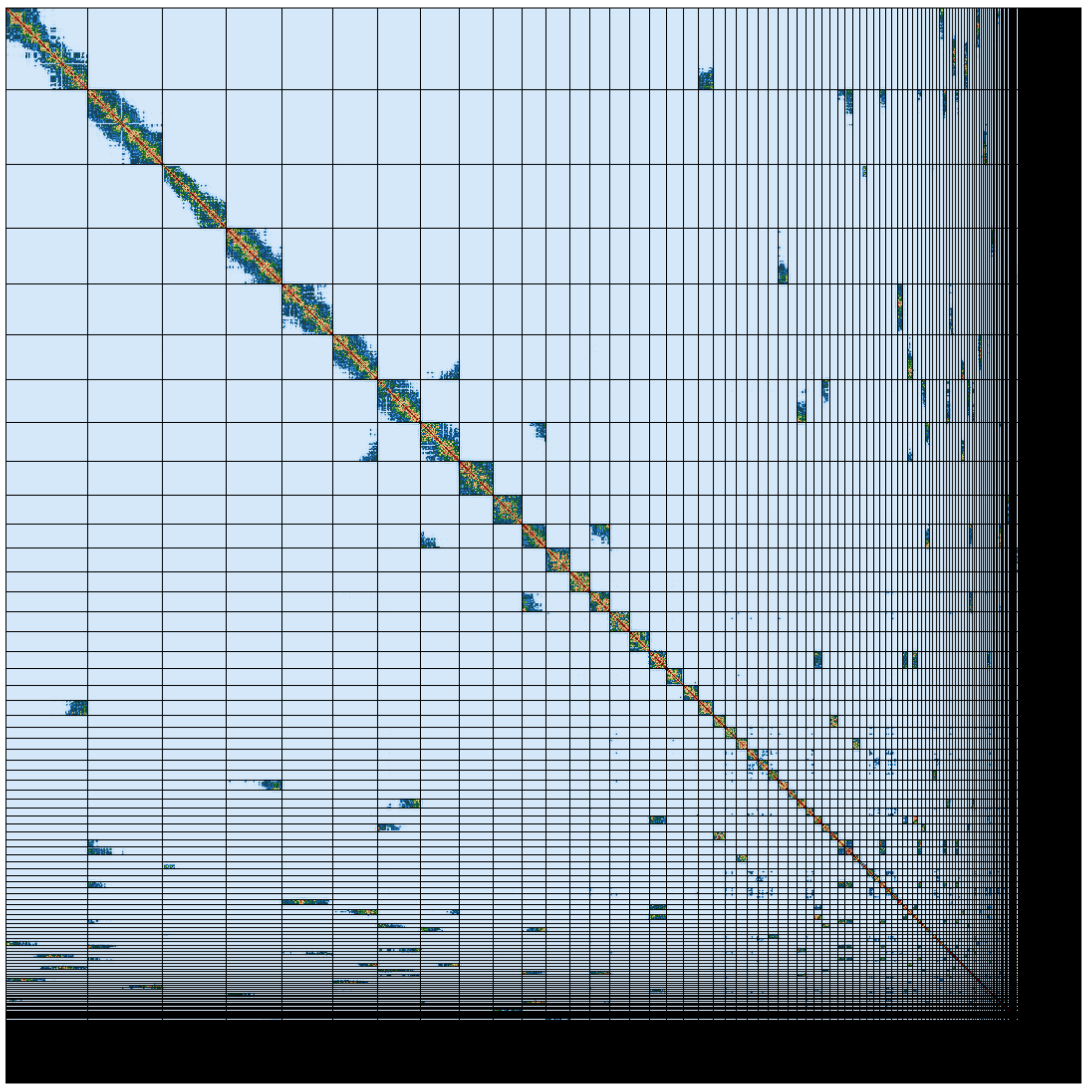

corvus__moneduloides HiC Heatmap (assembly 2)

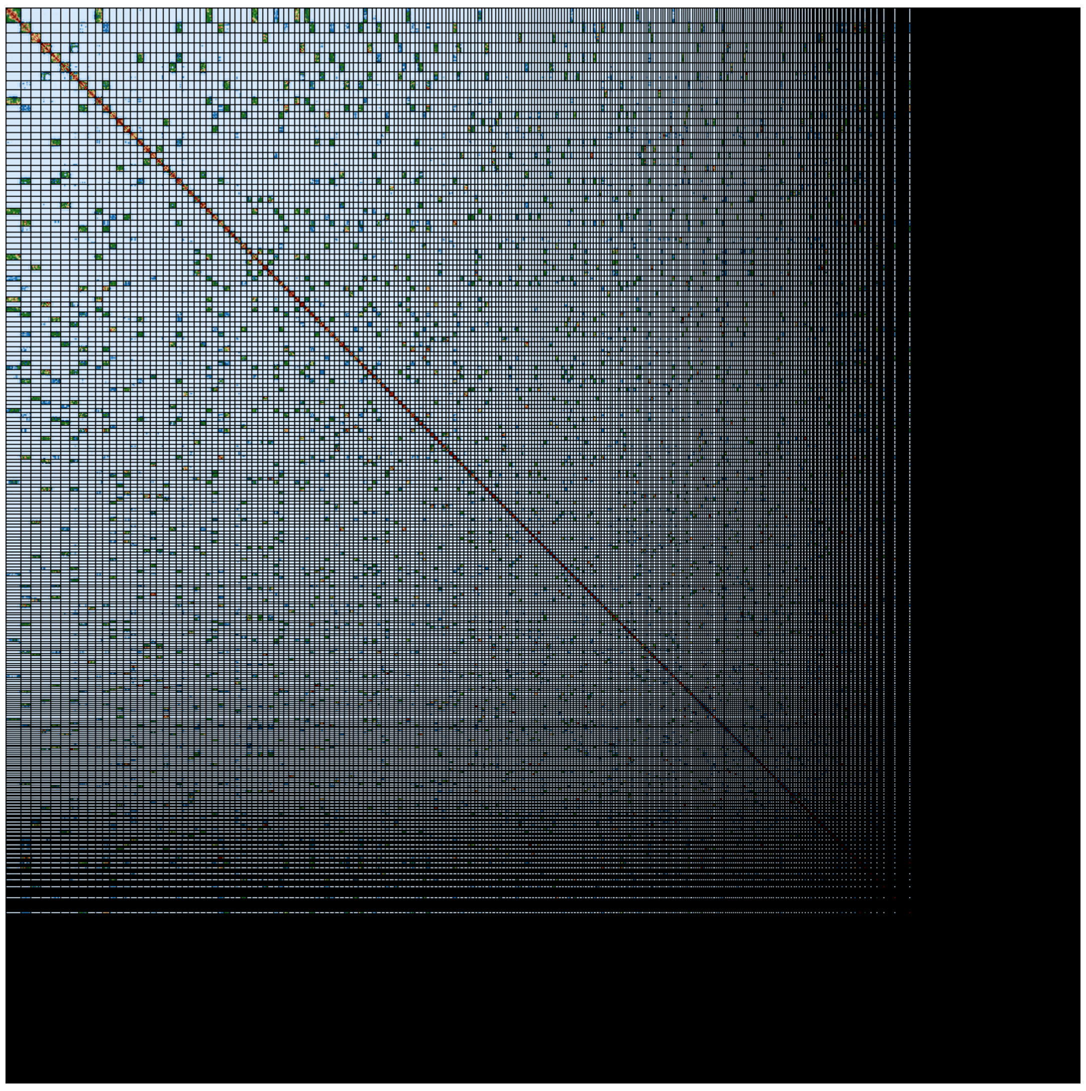

cyanocitta__stelleri HiC Heatmap (assembly 2)

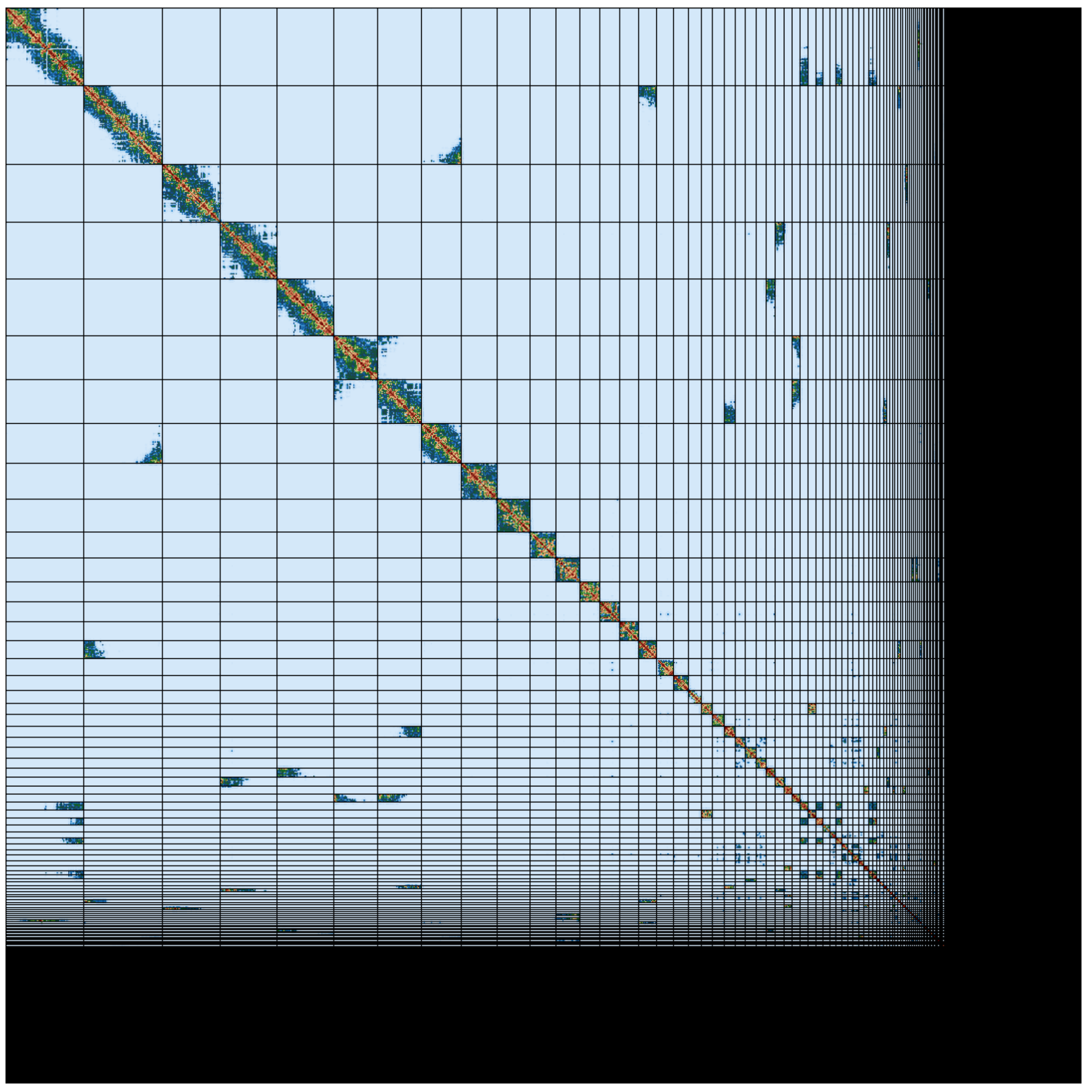

corvus__hawaiiensis HiC Heatmap (assembly 1)

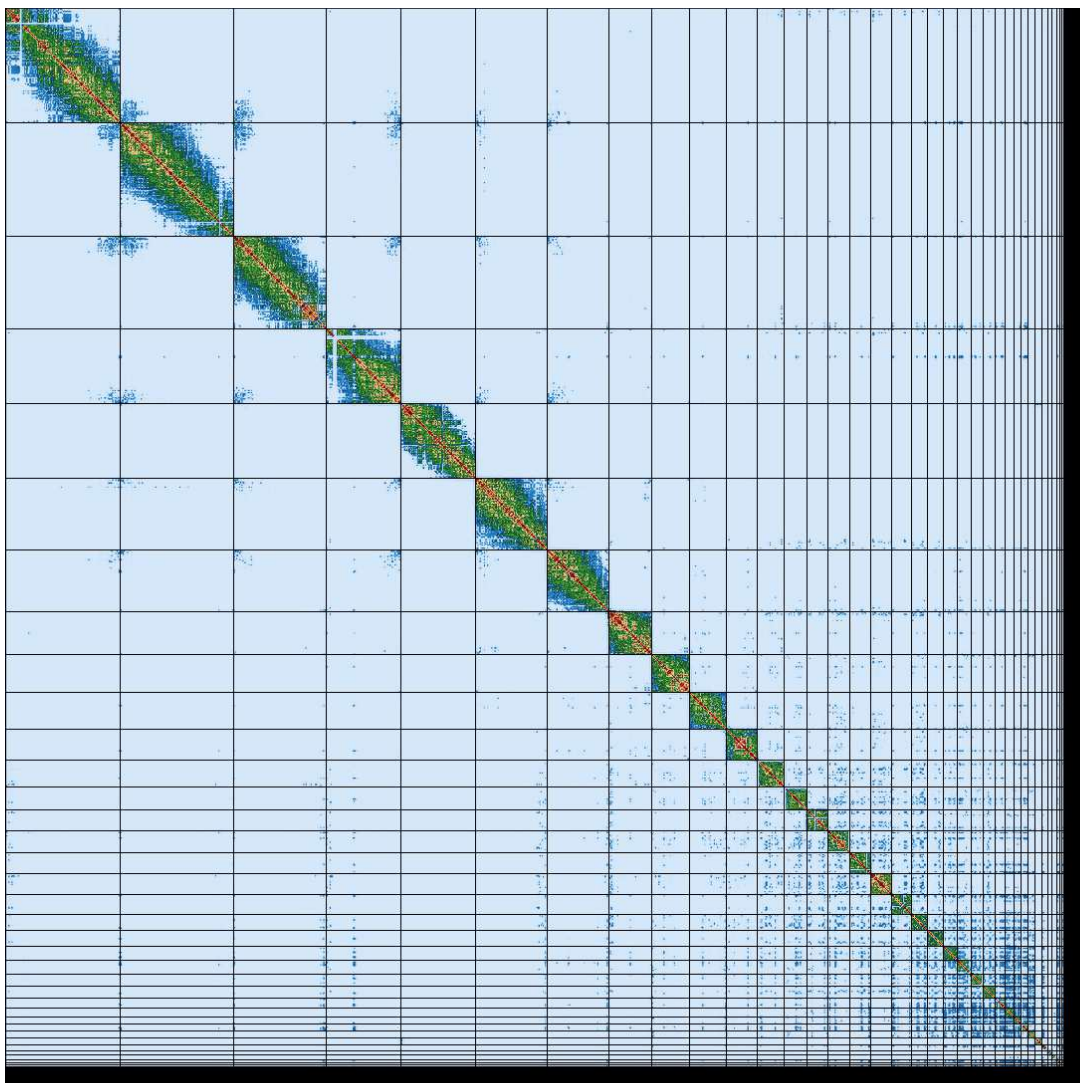

corvus__moneduloides HiC Heatmap (assembly 1)

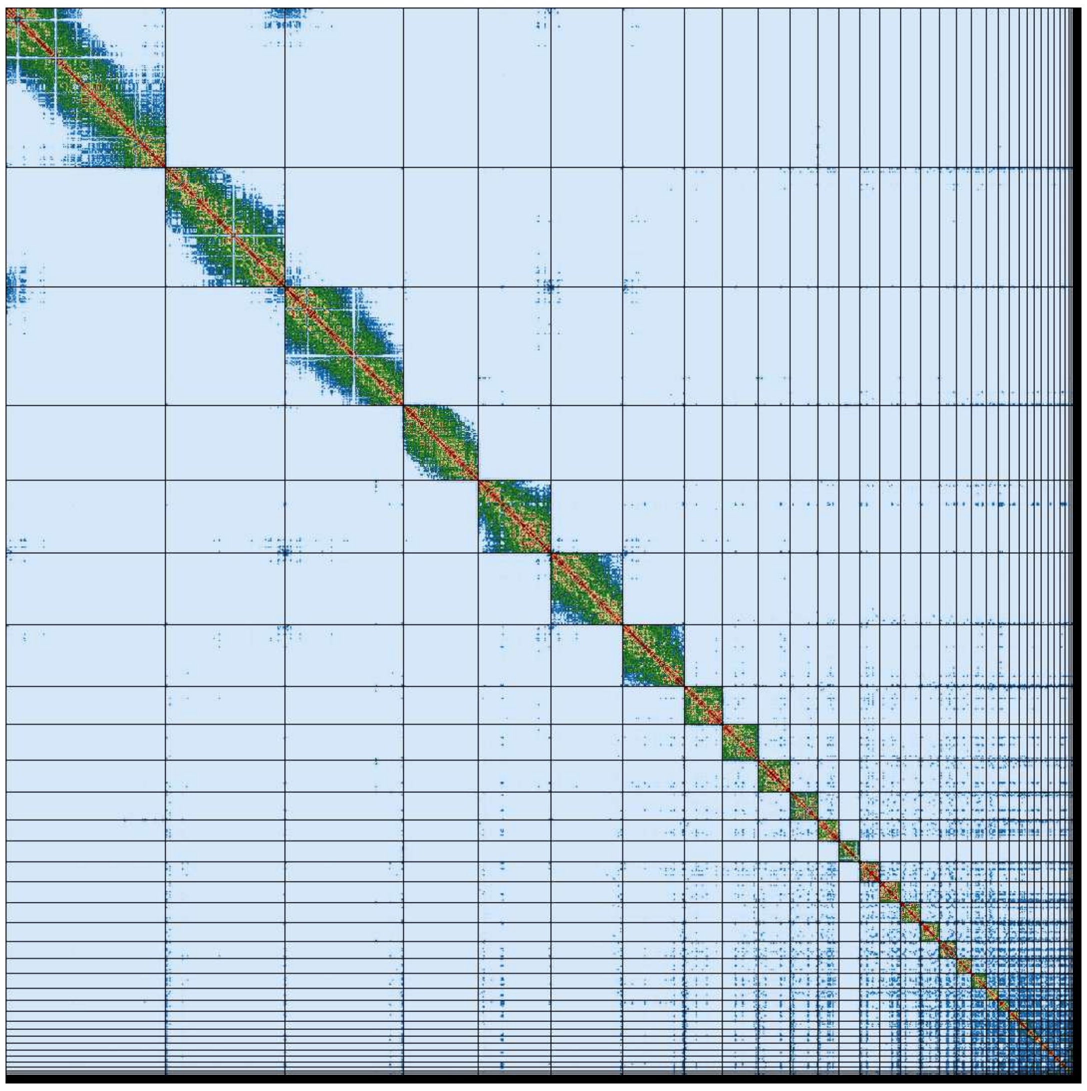

corvus__hawaiiensis HiC Heatmap (assembly 2)

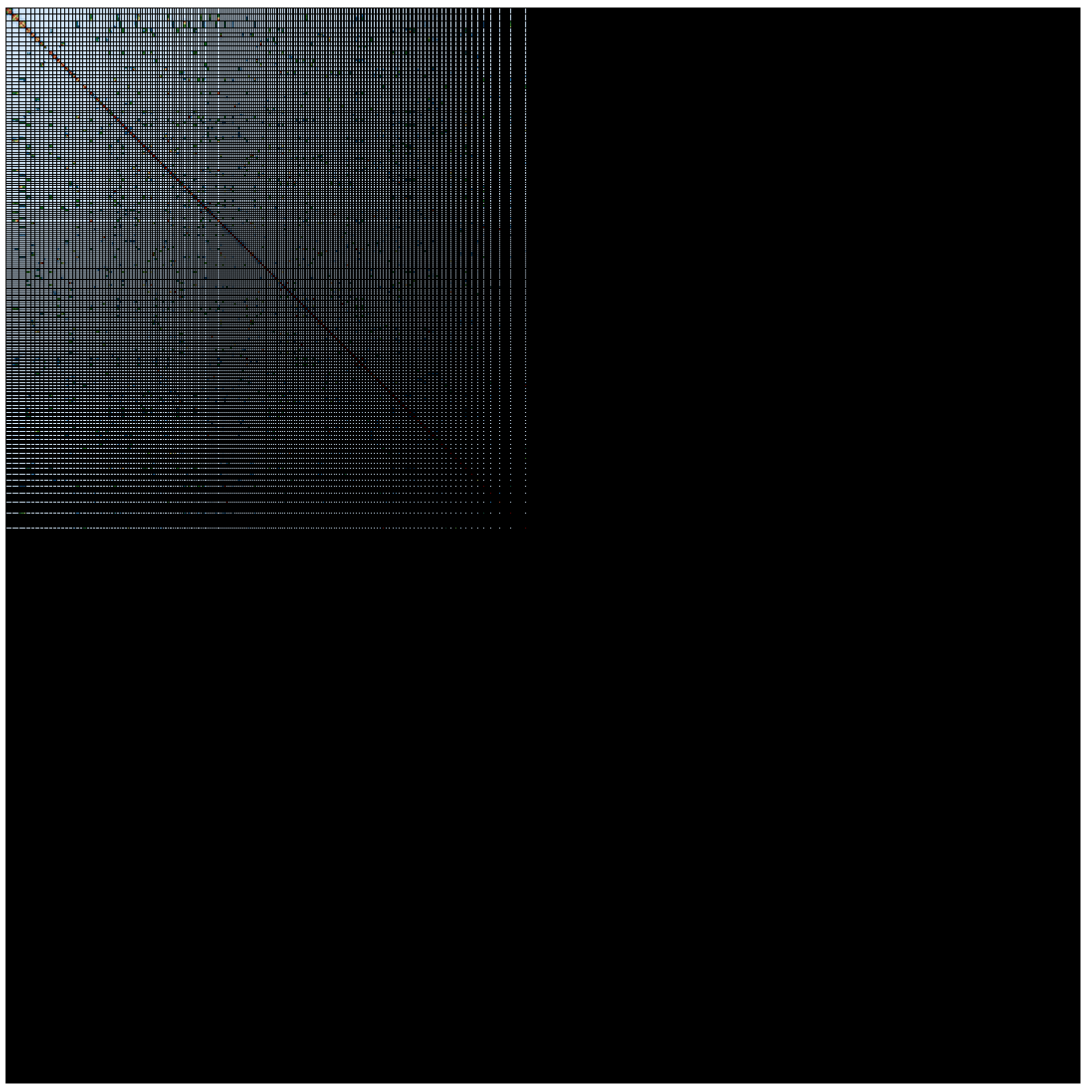

aphelocoma__californica HiC Heatmap (assembly 1)

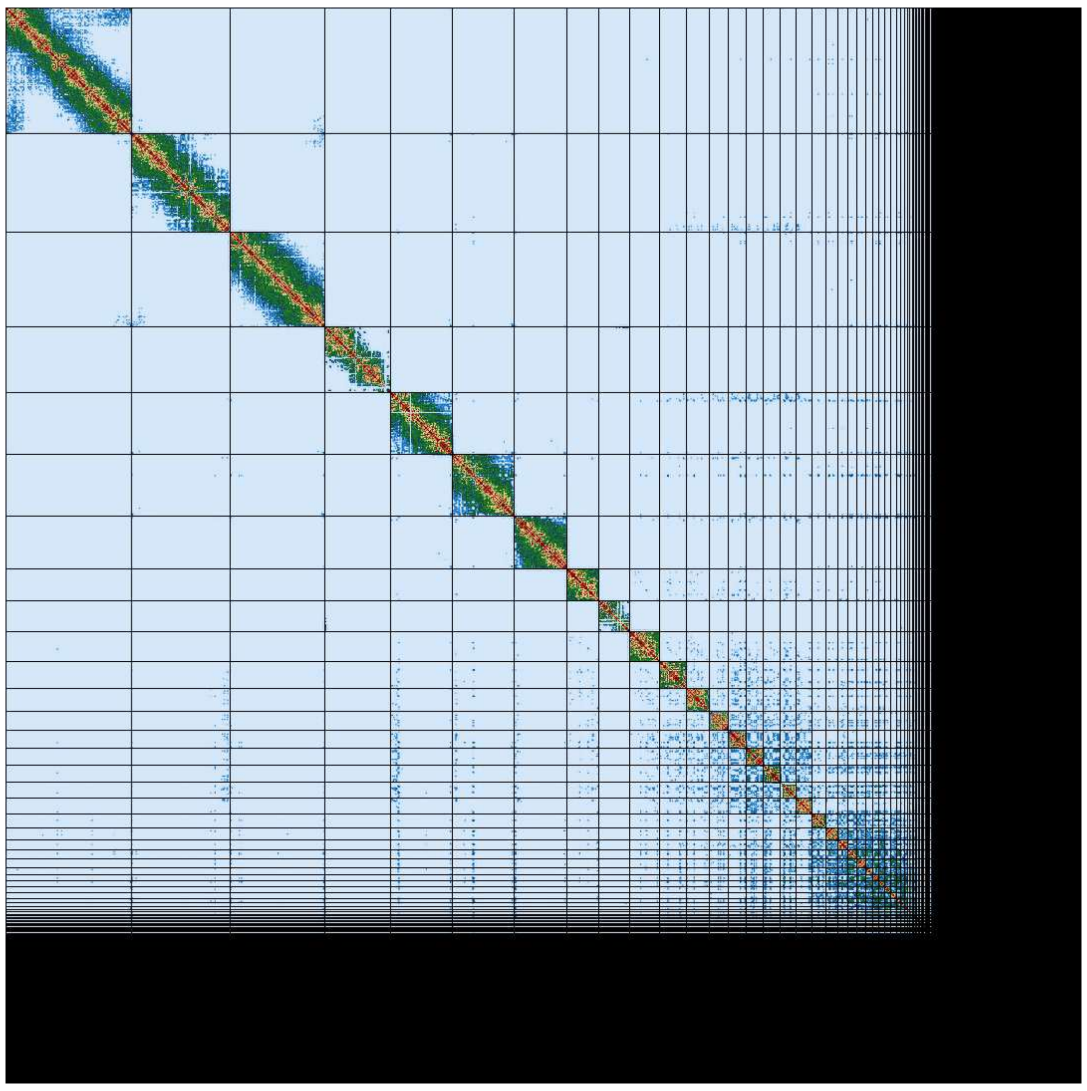

aphelocoma__californica HiC Heatmap (assembly 2)

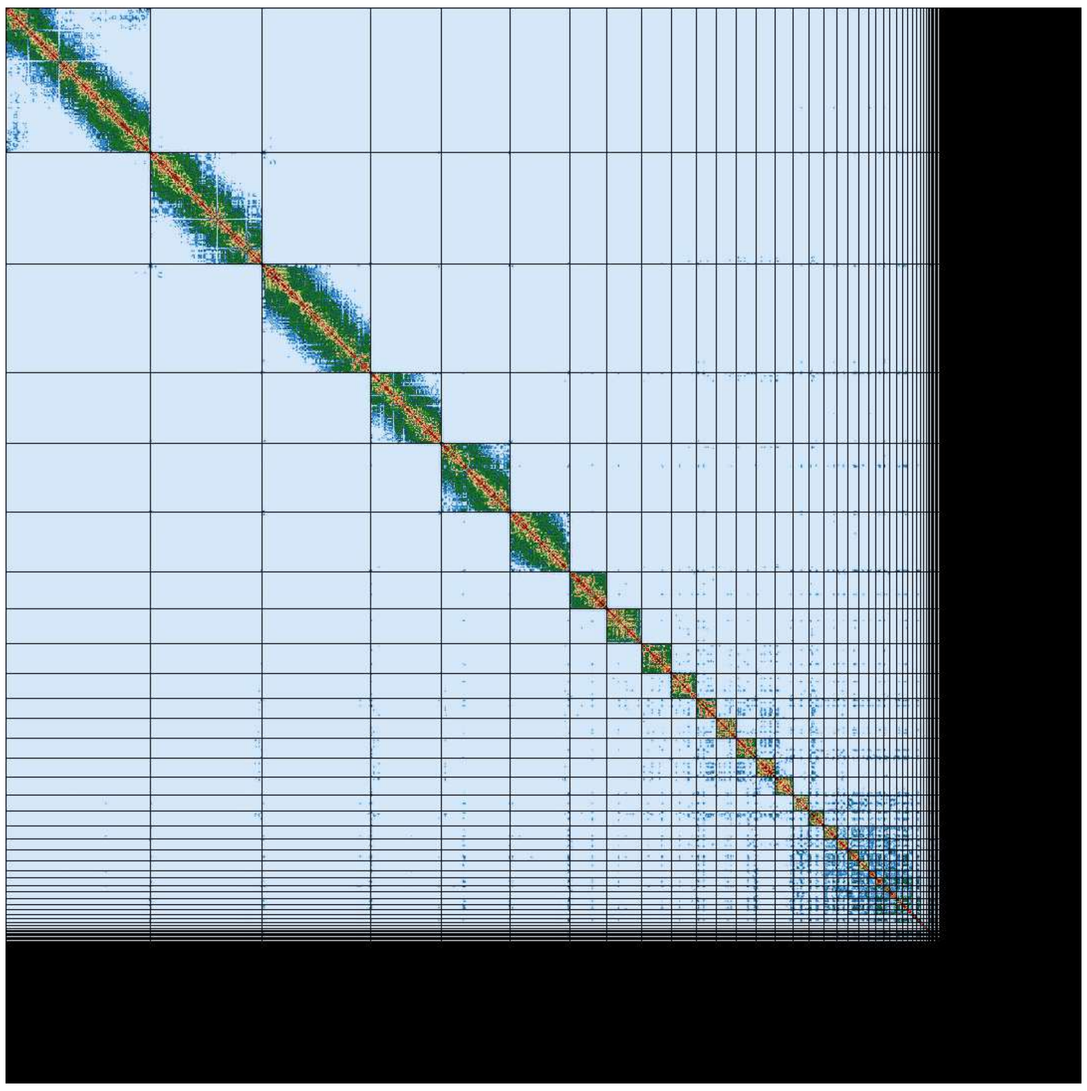

ASM_ID	Total_bp	GC_%	Gaps_per_Gb	Scaf	Cont	Longest_scaf	Scaf_N50	Scaf_L50	Scaf_N90	Longest_cont	Cont_N50	Cont_L50	Cont_N90	qv	Kmer_Compl	Comp_BUSCOs_%	Comp_Single_BUSCOs_%
pica_pica	1064369002	42.10	45569	24674	73177	153442850	73415481	5	11159609	567729	43520	6859	8877	57.4339	92.6015	95.6	95.5
oriolus_oriolus	1038866468	42.44	44791	22076	68608	4327510	716337	377	81853	513590	67624	4217	13130	43.2532	92.7765	94.2	94.0
garrulus_glandarius	1106508028	41.49	51888	48662	106077	153777791	72774246	6	7855194	328506	37784	8070	5669	54.0793	94.2164	95.8	95.5
cyanocitta_stelleri.asm1	1162964953	43.18	191	352	575	88620470	25842608	11	2628163	48674044	7854909	45	1180092	63.8786	94.3357	96.4	96.2
cyanocitta_stelleri.asm2	1177494911	44.10	169	957	1157	86024699	27786026	11	745298	40161822	6633807	44	659730	62.7371	88.4770	92.1	91.8
corvus_moneduloides.asm1	1112734206	42.57	259	106	395	165740467	74699841	5	14829806	46782926	11524098	30	2427983	43.8603	95.4097	96.3	95.8
corvus_moneduloides.asm2	905198257	42.67	0	894	894	12641944	2563033	105	593314	12641944	2563033	105	593314	40.6549	80.7650	82.8	82.6
corvus_macrorhynchos	1018435877	42.01	35131	16523	52302	3346143	133802	2023	26580	413299	49133	5606	10274	49.7258	92.8692	88.0	87.8
corvus_kubaryi.asm1	1057484666	42.14	10003	1443	12022	57584386	17891236	18	1633376	1366860	211131	1437	47382	53.6535	95.5615	94.2	91.0
corvus_kubaryi.asm2	1068123824	42.10	9994	1443	12118	57584386	17097965	19	1634489	1366860	211228	1453	47426	53.6814	96.9301	95.3	92.6
corvus_hawaiiensis.asm1	1151594481	43.30	255	187	481	123451405	76278832	6	13093161	80326523	23064755	15	2484873	42.4222	98.1658	96.8	96.5
corvus_hawaiiensis.asm2	628844469	43.16	0	2762	2762	4019362	584432	299	75900	4019362	584432	299	75900	47.6708	55.6481	54.6	54.4
corvus_brachyrhynchos	1091312783	41.85	72480	10547	89646	25919742	6953989	49	1168119	264908	29093	10509	6830	37.4894	94.9290	95.9	95.6
cnemophilus_loriae	1043492295	42.25	50534	17495	70227	7605640	1426893	200	233074	499561	67183	4330	13765	42.9756	91.1503	95.8	95.5
aphelocoma_coerulescens	1060855343	42.08	21217	1701	24210	29167858	7634200	42	1297942	731130	118847	2548	26590	47.0009	94.5721	96.4	96.2
aphelocoma_californica.asm1	1348500425	44.58	201	1393	1665	158410743	66144195	7	468665	77933725	11534525	28	403960	63.3494	92.3751	96.4	95.9
aphelocoma_californica.asm2	1191279315	44.29	141	1631	1799	160920769	65728546	6	489716	52308079	11670509	27	394688	63.6952	85.6641	92.2	91.9

Total_bp	GC_%	Gaps_per_Gb	Scaf	Cont	Longest_scaf	Scaf_N50	Scaf_L50	Scaf_N90	Longest_cont	Cont_N50	Cont_L50	Cont_N90	qv	Kmer_Compl	Comp_BUSCOs_%	Comp_Single_BUSCOs_%
Max	Max	Max	Max	Max	Max	Max	Max	Max	Max	Max	Max	Max	Max	Max	Max	Max
Min	Min	Min	Min	Min	Min	Min	Min	Min	Min	Min	Min	Min	Min	Min	Min	Min

ASM_ID	Gaps_per_Gb	Scaf_N50	Cont_N50	qv	Kmer_Compl	Comp_Single_BUSCOs_%
pica_pica	45569	73415481	43520	57.4339	92.6015	95.5
oriolus_oriolus	44791	716337	67624	43.2532	92.7765	94.0
garrulus_glandarius	51888	72774246	37784	54.0793	94.2164	95.5
cyanocitta_stelleri.asm1	191	25842608	7854909	63.8786	94.3357	96.2
cyanocitta_stelleri.asm2	169	27786026	6633807	62.7371	88.4770	91.8
corvus_moneduloides.asm1	259	74699841	11524098	43.8603	95.4097	95.8
corvus_moneduloides.asm2	0	2563033	2563033	40.6549	80.7650	82.6
corvus_macrorhynchos	35131	133802	49133	49.7258	92.8692	87.8
corvus_kubaryi.asm1	10003	17891236	211131	53.6535	95.5615	91.0
corvus_kubaryi.asm2	9994	17097965	211228	53.6814	96.9301	92.6
corvus_hawaiiensis.asm1	255	76278832	23064755	42.4222	98.1658	96.5
corvus_hawaiiensis.asm2	0	584432	584432	47.6708	55.6481	54.4
corvus_brachyrhynchos	72480	6953989	29093	37.4894	94.9290	95.6
cnemophilus_loriae	50534	1426893	67183	42.9756	91.1503	95.5
aphelocoma_coerulescens	21217	7634200	118847	47.0009	94.5721	96.2
aphelocoma_californica.asm1	201	66144195	11534525	63.3494	92.3751	95.9
aphelocoma_californica.asm2	141	65728546	11670509	63.6952	85.6641	91.9

Gaps_per_Gb	Scaf_N50	Cont_N50	qv	Kmer_Compl	Comp_Single_BUSCOs_%
< 200	> 100Mbp	> 10Mbp	> 50	> 95%	> 95%
200 - 1000	10Mbp - 100Mbp	1Mbp - 10Mbp	40 - 50	90% - 95%	90% - 95%
1000 - 10000	0.1Mbp - 10Mbp	0.01Mbp - 1Mbp	35 - 40	80% - 90%	80% - 90%
> 10000	< 0.1Mbp	< 0.01Mbp	< 35	< 80%	< 80%

Case 2a: Full assembly pipeline evaluation

Assembly ID: Scapyr_cont

Database built with kmer size: 21 bp

Number of haploptypes/assemblies: 2

Expected genome size: 300901319

Heterozygosity: 2.322 %

QV Score

assembly_name	Assembly_Only	Total_Kmers	QV_Score
Scapyr_cont.asm1	56735	661890069	53.8914
Scapyr_cont.asm2	11733	34111974	47.8565
Both	68468	696002043	53.2932

Kmer Completeness

assembly_name	Kmers_Assembly	Kmers_Reads	%
Scapyr_cont.asm1	359238784	364489292	98.5595
Scapyr_cont.asm2	14170545	364489292	3.88778
both	359247051	364489292	98.5618

BUSCOv5 (database: insecta)

Metric	asm1 #	asm1 %	asm2 #	asm2 %
Complete	1354	99.0	50	3.7
Complete and single-copy	17	1.2	31	2.3
Complete and duplicated	1337	97.8	19	1.4
Fragmented	0	0.0	6	0.4
Missing	13	1.0	1311	95.9
Total searched groups	1367	100.0	1367	100.0

Genomescope2 Profile (Linear)

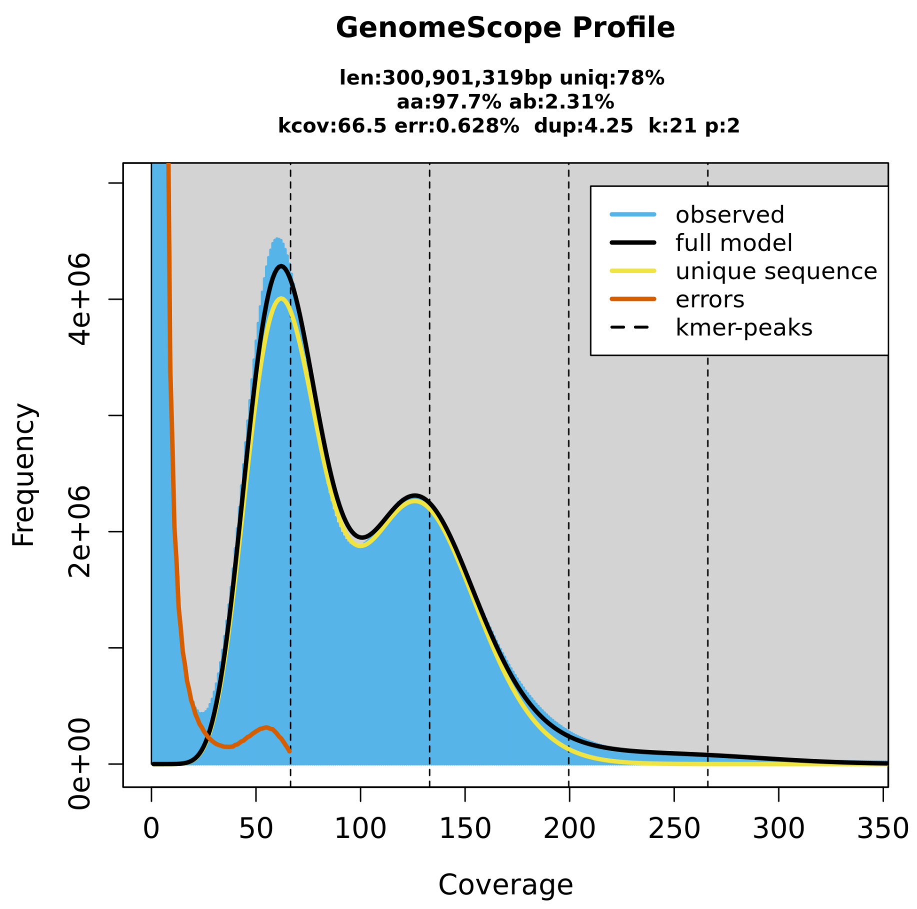

Smudgeplot Profile

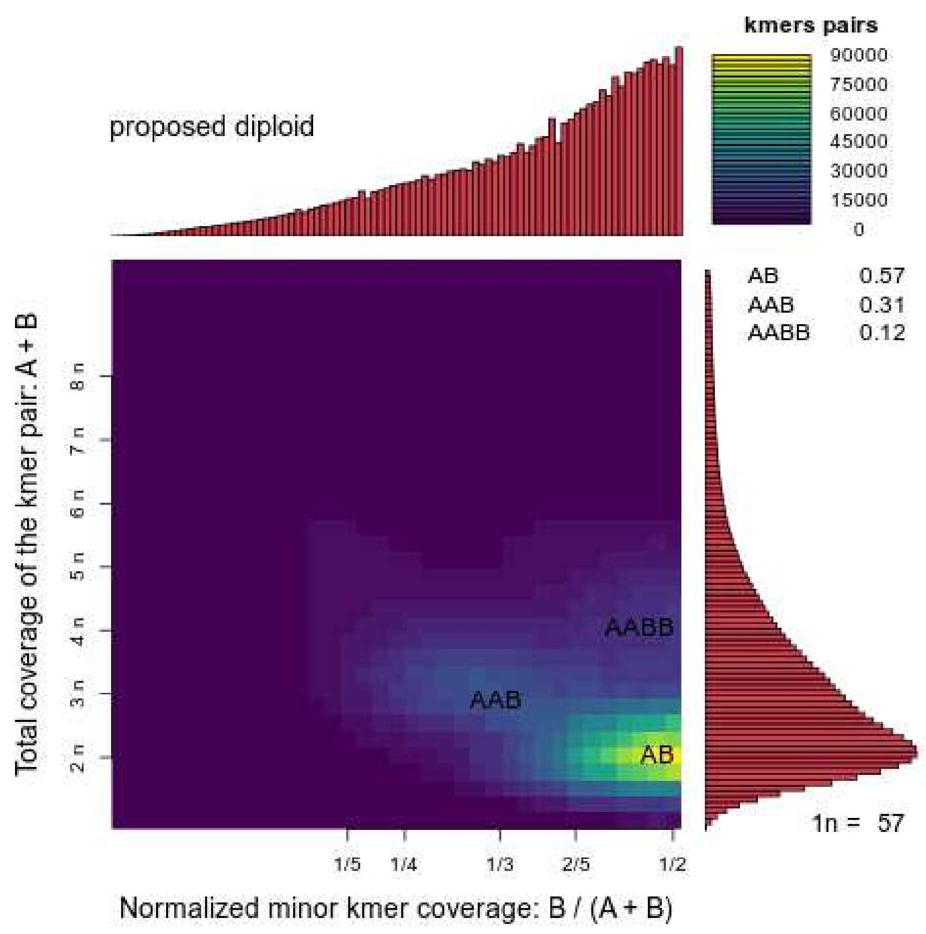

Genome Statistics with gfastats

Metric	asm1	asm2
Total scaffold length	661901849	34141814
GC content %	32.78	32.26
# gaps in scaffolds	0	0
Total gap length in scaffolds	0	0
# scaffolds	589	1492
Largest scaffold	43965498	491049
Scaffold N50	11589531	23693
Scaffold L50	16	522
Scaffold L90	106	1223
# contigs	589	1492
Largest contig	43965498	491049
Contig N50	11589531	23693
Contig L50	16	522
Contig L90	106	1223

K-mer Multiplicity asm1 Only (Flat)

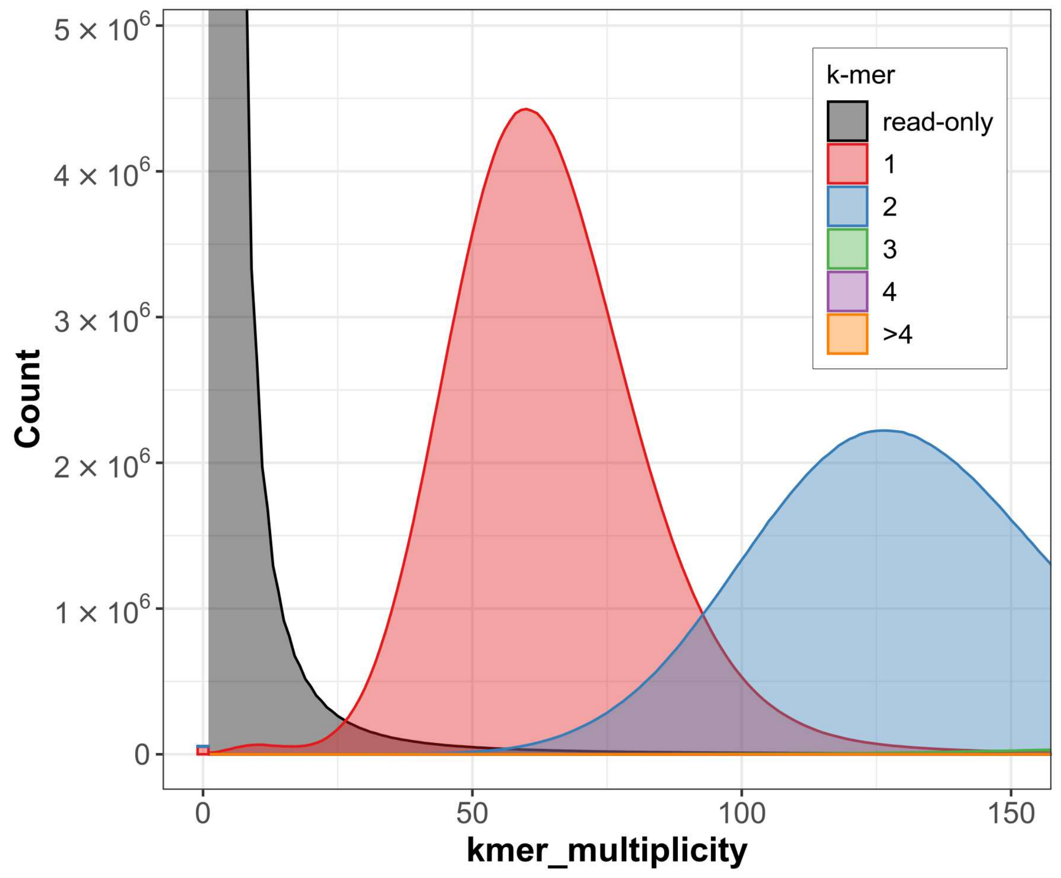

K-mer Multiplicity asm1 + asm2 (Flat)

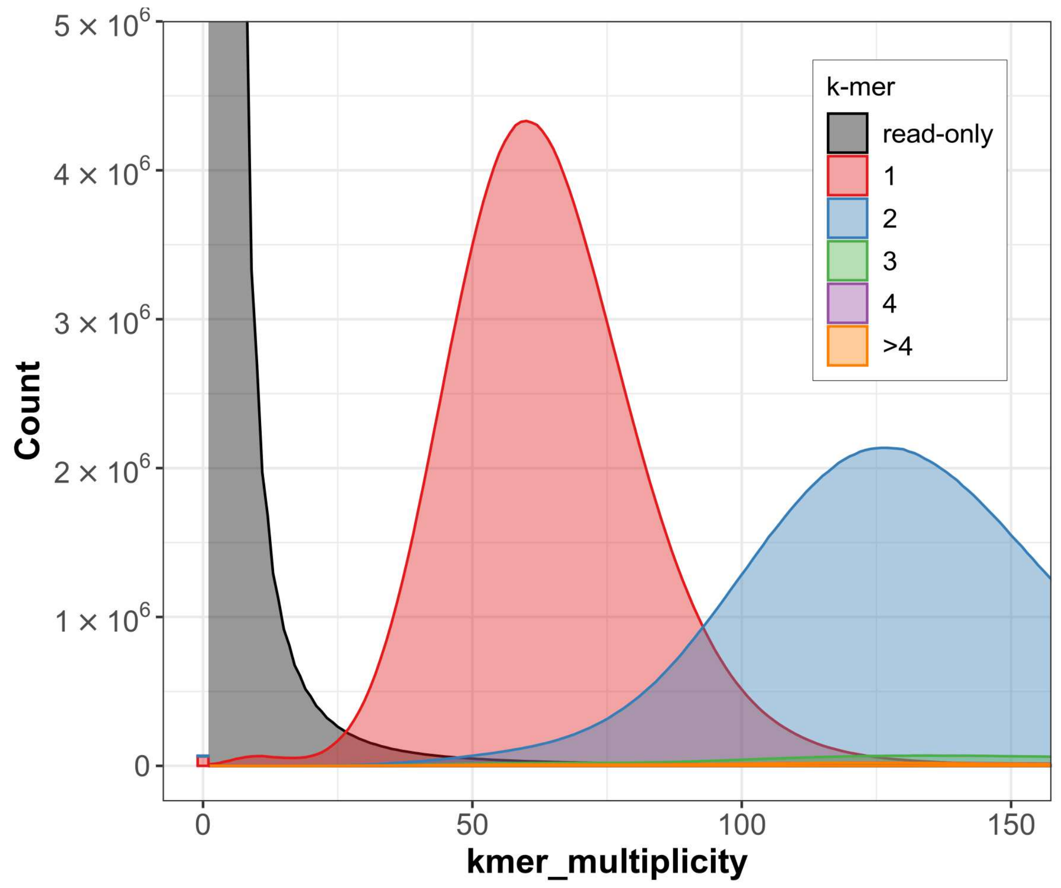

Assembly ID: Scapyr_purg

Database built with kmer size: 21 bp

Number of haploptypes/assemblies: 2

Expected genome size: 300901319

Heterozygosity: 2.322 %

QV Score

assembly_name	Assembly_Only	Total_Kmers	QV_Score
Scapyr_purg.asm1	41524	339502360	52.3474
Scapyr_purg.asm2	11114	321265475	57.8321
Both	52638	660767835	54.2095

Kmer Completeness

assembly_name	Kmers_Assembly	Kmers_Reads	%
Scapyr_purg.asm1	266490206	364489292	73.1133
Scapyr_purg.asm2	265171642	364489292	72.7516
both	359210063	364489292	98.5516

BUSCOv5 (database: insecta)

Metric	asm1 #	asm1 %	asm2 #	asm2 %
Complete	1354	99.1	1350	98.8
Complete and single-copy	1349	98.7	1342	98.2
Complete and duplicated	5	0.4	8	0.6
Fragmented	0	0.0	2	0.1
Missing	13	0.9	15	1.1
Total searched groups	1367	100.0	1367	100.0

Genomescope2 Profile (Linear)

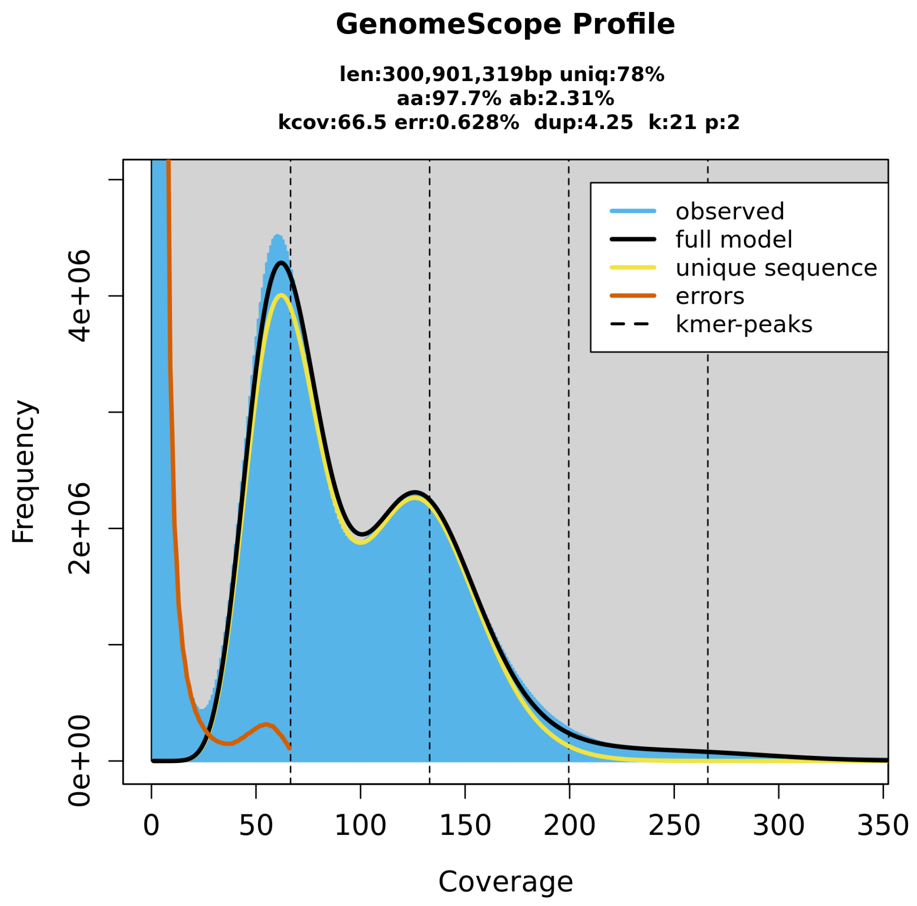

Smudgeplot Profile

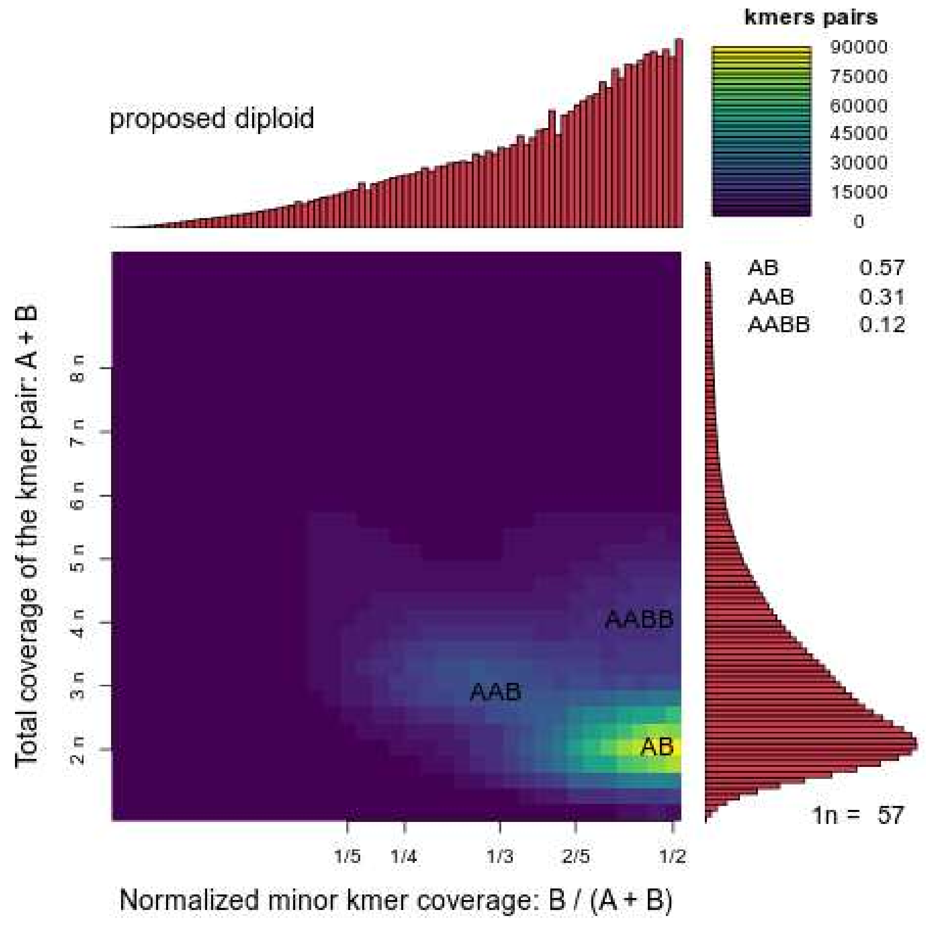

Genome Statistics with gfastats

Metric	asm1	asm2
Total scaffold length	339508160	321271535
GC content %	32.98	32.63
# gaps in scaffolds	0	0
Total gap length in scaffolds	0	0
# scaffolds	290	303
Largest scaffold	43965498	43837227
Scaffold N50	14568262	9868577
Scaffold L50	8	9
Scaffold L90	56	53
# contigs	290	303
Largest contig	43965498	43837227
Contig N50	14568262	9868577
Contig L50	8	9
Contig L90	56	53

K-mer Multiplicity asm1 Only (Flat)

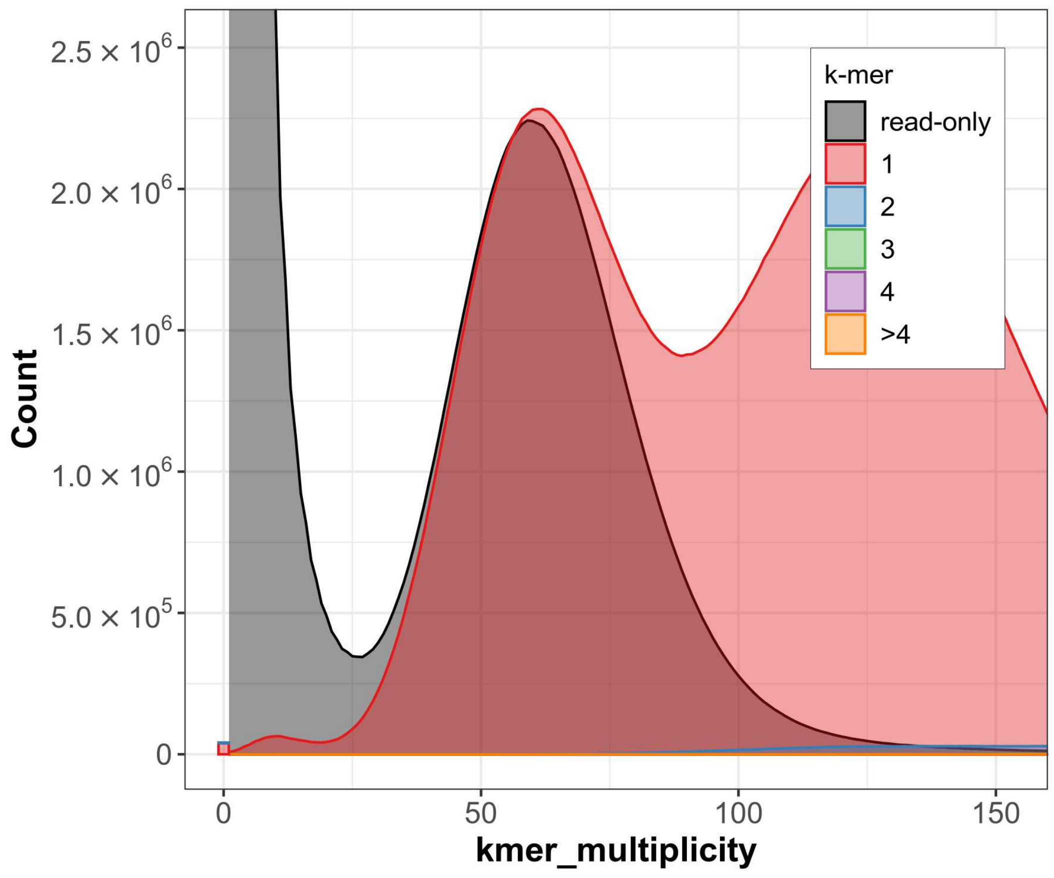

K-mer Multiplicity asm1 + asm2 (Flat)

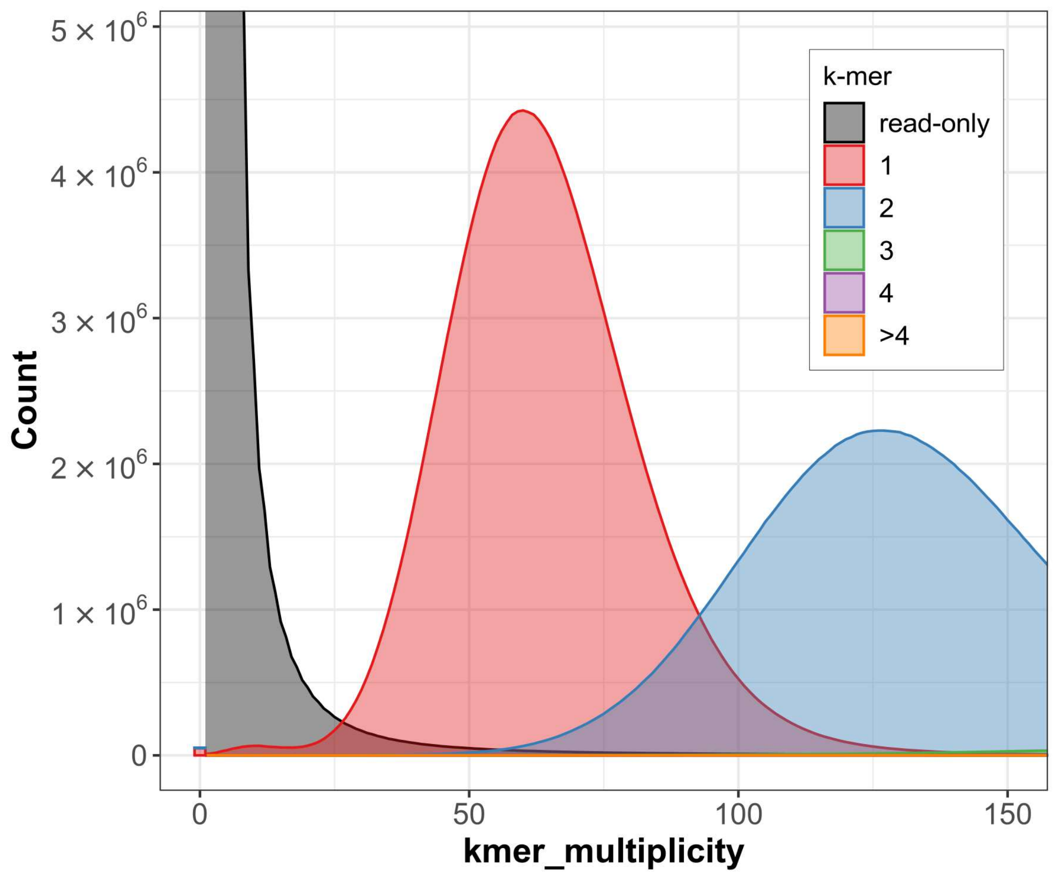

Assembly ID: Scapyr_scaf

Database built with kmer size: 21 bp

Number of haploptypes/assemblies: 2

Expected genome size: 300901319

Heterozygosity: 2.322 %

QV Score

assembly_name	Assembly_Only	Total_Kmers	QV_Score
Scapyr_scaf.asm1	41524	339502280	52.3474
Scapyr_scaf.asm2	11114	321265475	57.8321
Both	52638	660767755	54.2095

Kmer Completeness

assembly_name	Kmers_Assembly	Kmers_Reads	%
Scapyr_scaf.asm1	266490186	364489292	73.1133
Scapyr_scaf.asm2	265171642	364489292	72.7516
both	359210063	364489292	98.5516

BUSCOv5 (database: insecta)

Metric	asm1 #	asm1 %	asm2 #	asm2 %
Complete	1355	99.2	1350	98.8
Complete and single-copy	1350	98.8	1342	98.2
Complete and duplicated	5	0.4	8	0.6
Fragmented	0	0.0	2	0.1
Missing	12	0.8	15	1.1
Total searched groups	1367	100.0	1367	100.0

Genomescope2 Profile (Linear)

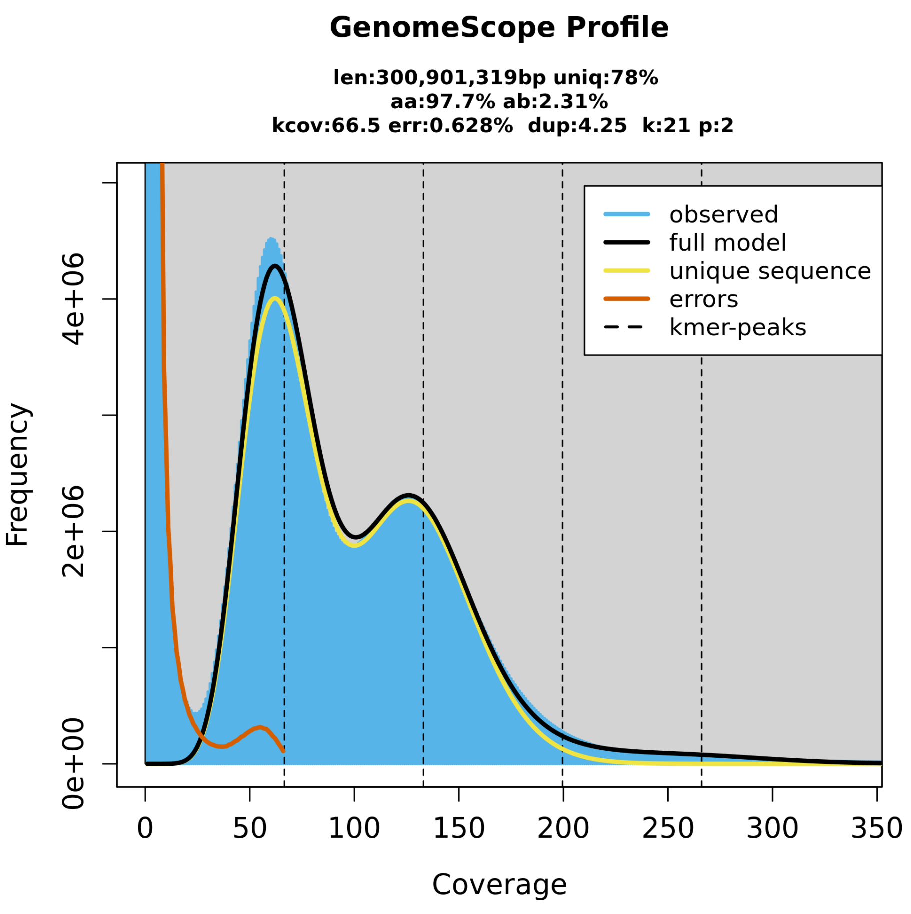

Smudgeplot Profile

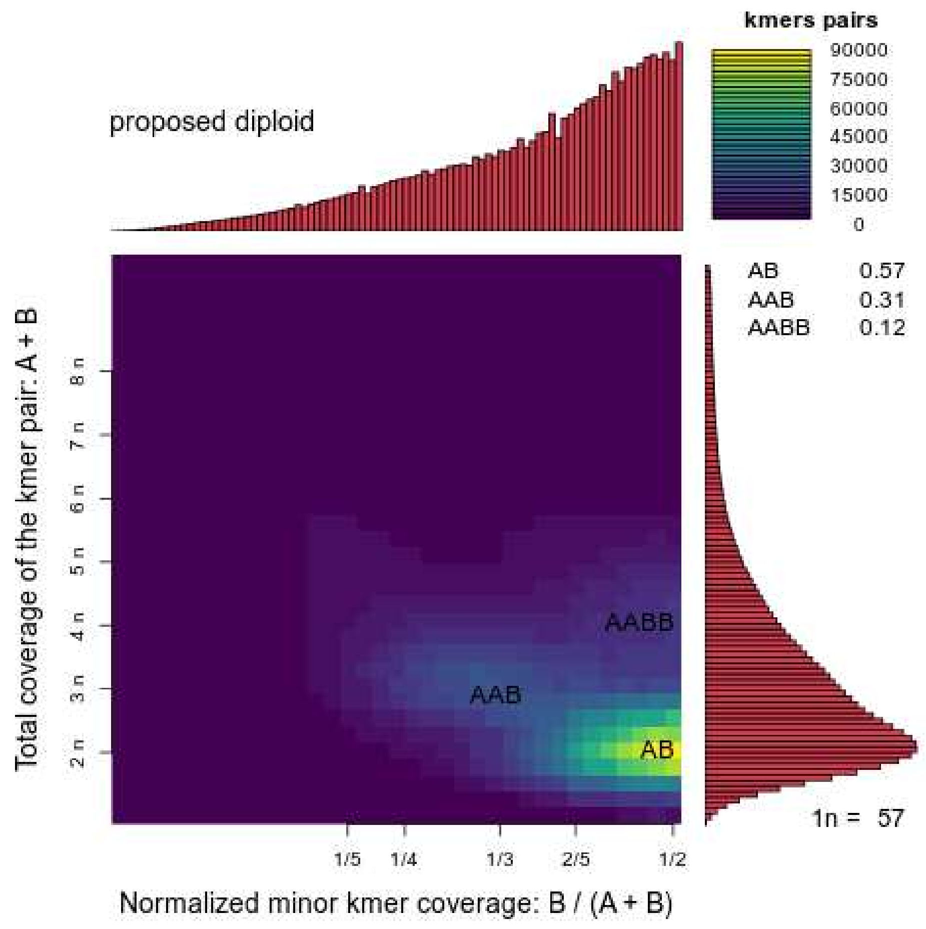

Genome Statistics with gfastats

Metric	asm1	asm2
Total scaffold length	339557660	321271535
GC content %	32.98	32.63
# gaps in scaffolds	99	0
Total gap length in scaffolds	49500	0
# scaffolds	195	303
Largest scaffold	71711308	43837227
Scaffold N50	47615032	9868577
Scaffold L50	3	9
Scaffold L90	14	53
# contigs	294	303
Largest contig	43965498	43837227
Contig N50	14568262	9868577
Contig L50	8	9
Contig L90	60	53

K-mer Multiplicity asm1 Only (Flat)

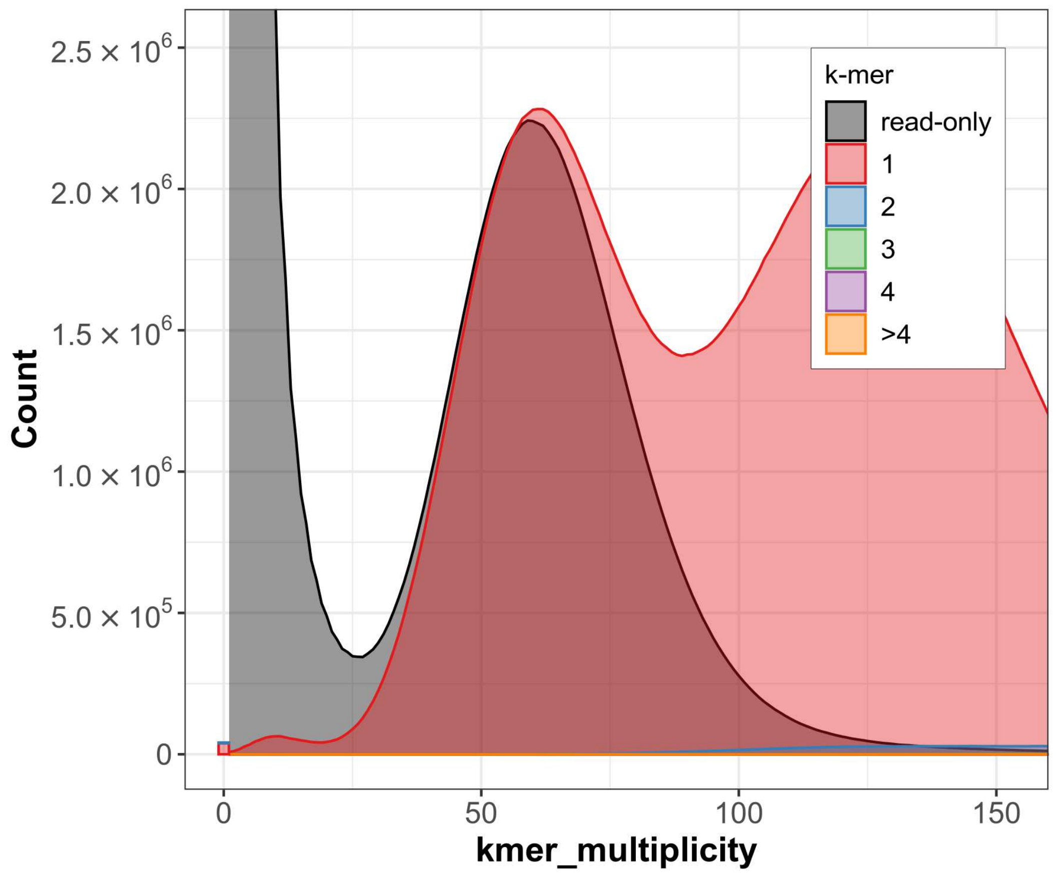

K-mer Multiplicity asm1 + asm2 (Flat)

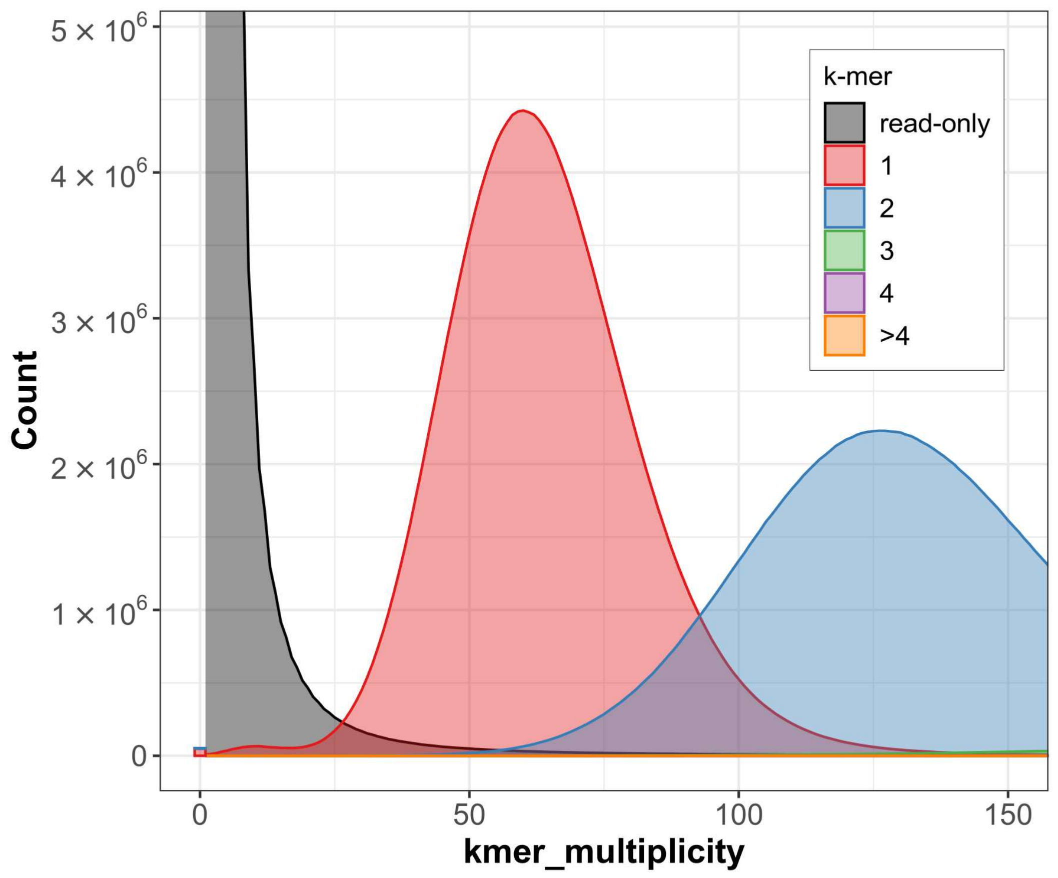

Assembly ID: Scapyr_final

Database built with kmer size: 21 bp

Number of haploptypes/assemblies: 2

Expected genome size: 300901319

Heterozygosity: 2.322 %

QV Score

assembly_name	Assembly_Only	Total_Kmers	QV_Score
Scapyr_final.asm1	18499	339499852	55.859
Scapyr_final.asm2	8651	321264511	58.9201
Both	27150	660764363	57.0849

Kmer Completeness

assembly_name	Kmers_Assembly	Kmers_Reads	%
Scapyr_final.asm1	266502815	364489292	73.1168
Scapyr_final.asm2	265179214	364489292	72.7536
both	359225422	364489292	98.5558

BUSCOv5 (database: insecta)

Metric	asm1 #	asm1 %	asm2 #	asm2 %
Complete	1355	99.2	1350	98.8
Complete and single-copy	1350	98.8	1342	98.2
Complete and duplicated	5	0.4	8	0.6
Fragmented	0	0.0	2	0.1
Missing	12	0.8	15	1.1
Total searched groups	1367	100.0	1367	100.0

Genomescope2 Profile (Linear)

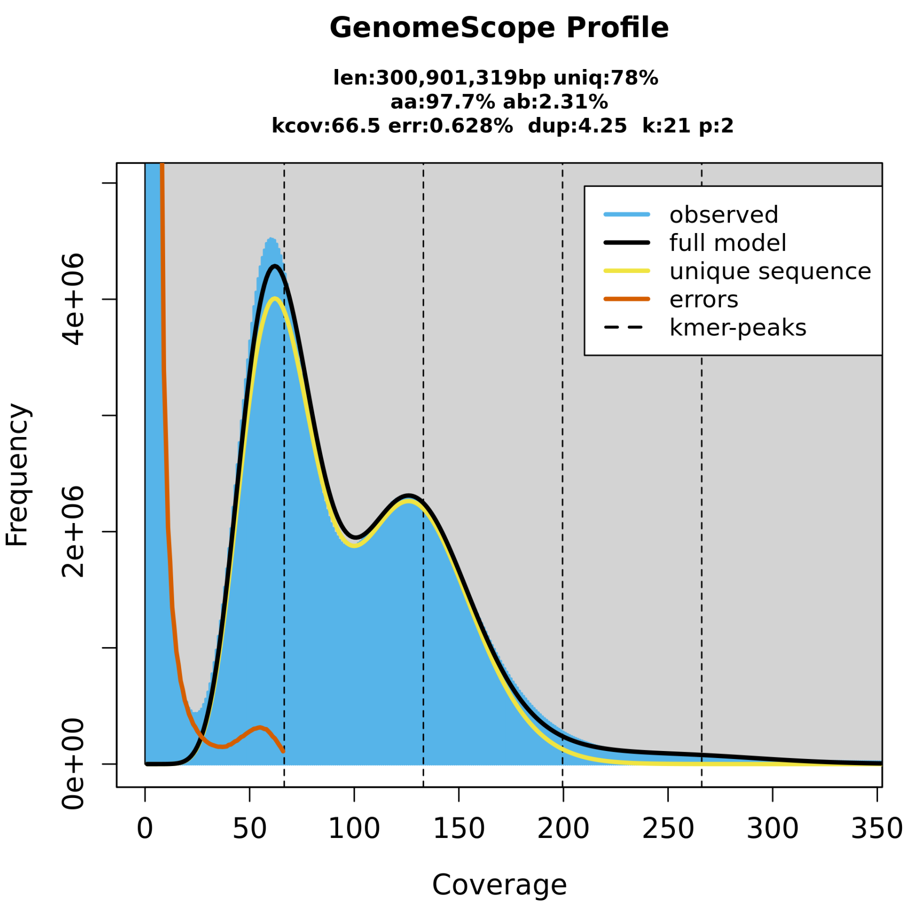

Smudgeplot Profile

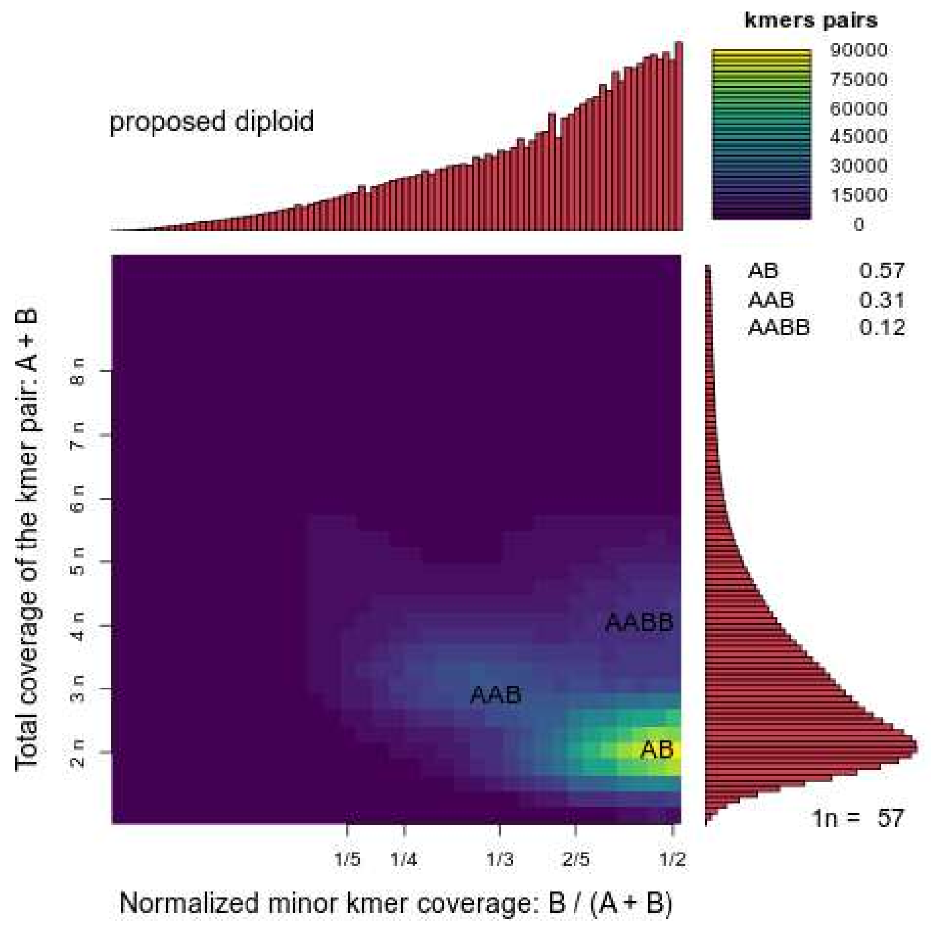

Genome Statistics with gfastats

Metric	asm1	asm2
Total scaffold length	339555232	321270571
GC content %	32.98	32.63
# gaps in scaffolds	99	0
Total gap length in scaffolds	49500	0
# scaffolds	195	303
Largest scaffold	71711214	43837241
Scaffold N50	47615007	9868581
Scaffold L50	3	9
Scaffold L90	14	53
# contigs	294	303
Largest contig	43965472	43837241
Contig N50	14568203	9868581
Contig L50	8	9
Contig L90	60	53

K-mer Multiplicity asm1 Only (Flat)

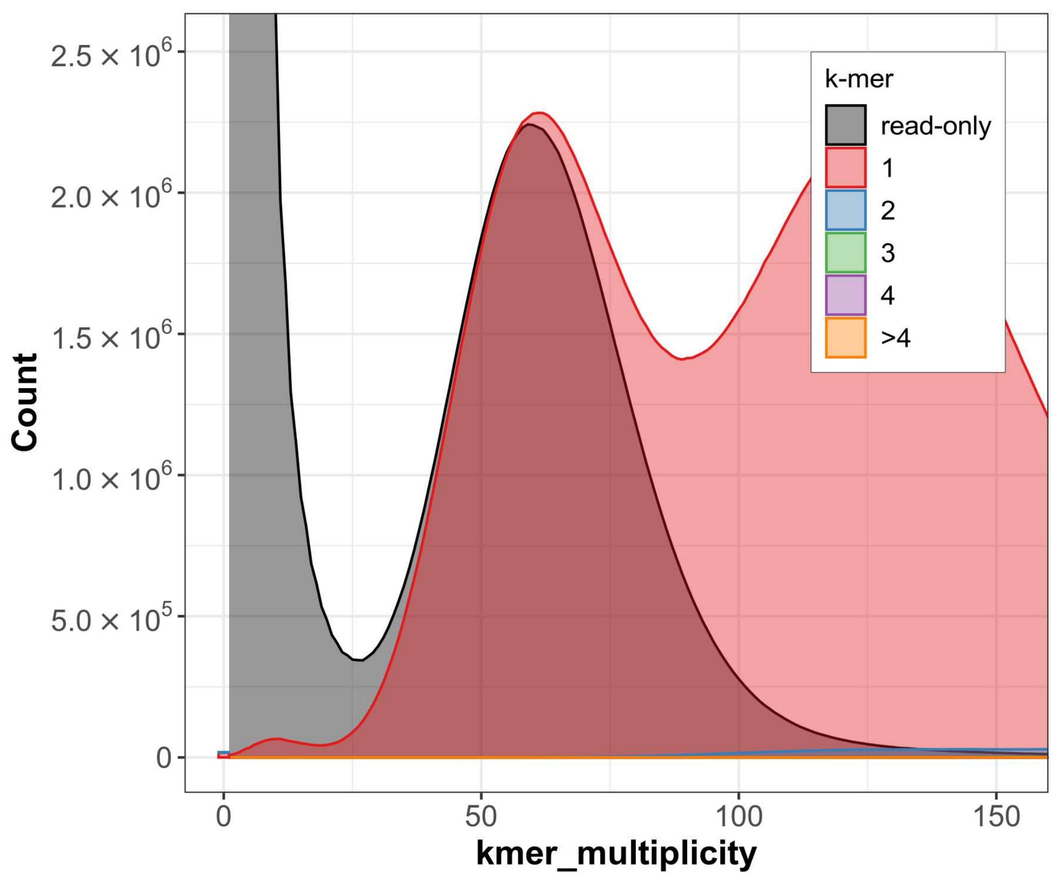

K-mer Multiplicity asm1 + asm2 (Flat)

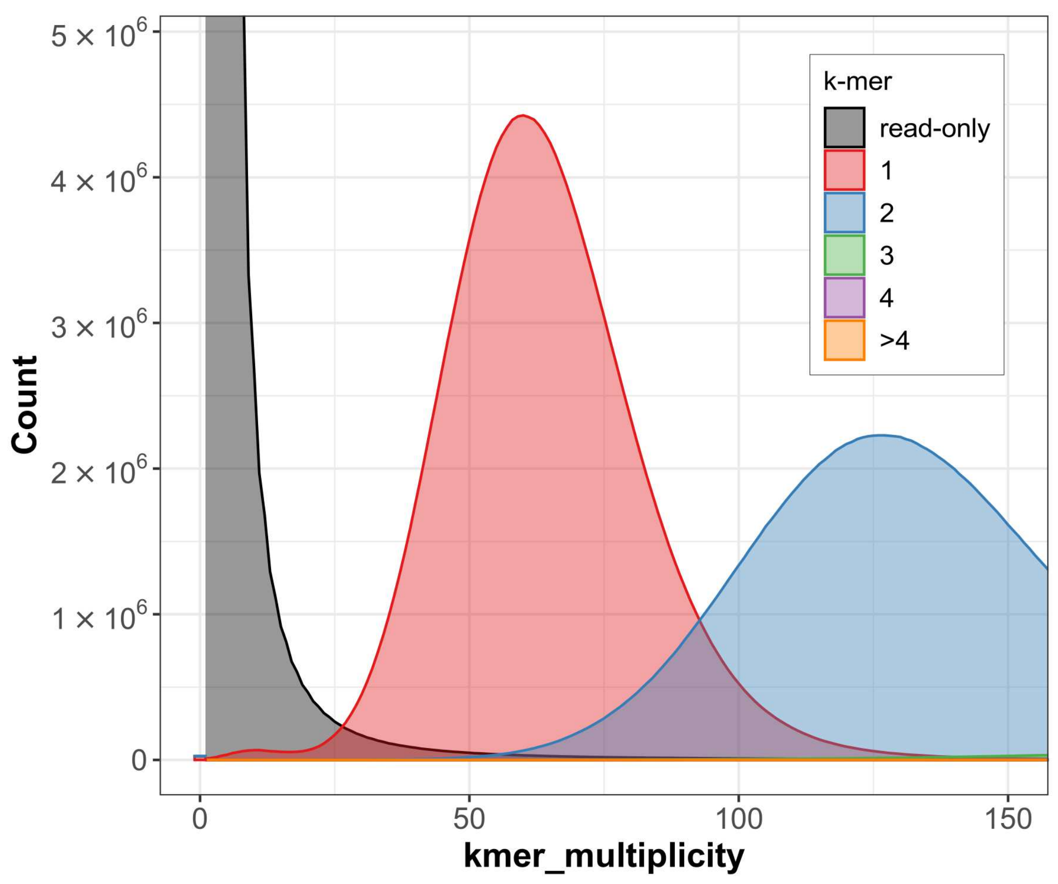

Scapyr__cont HiC Heatmap (assembly 1)

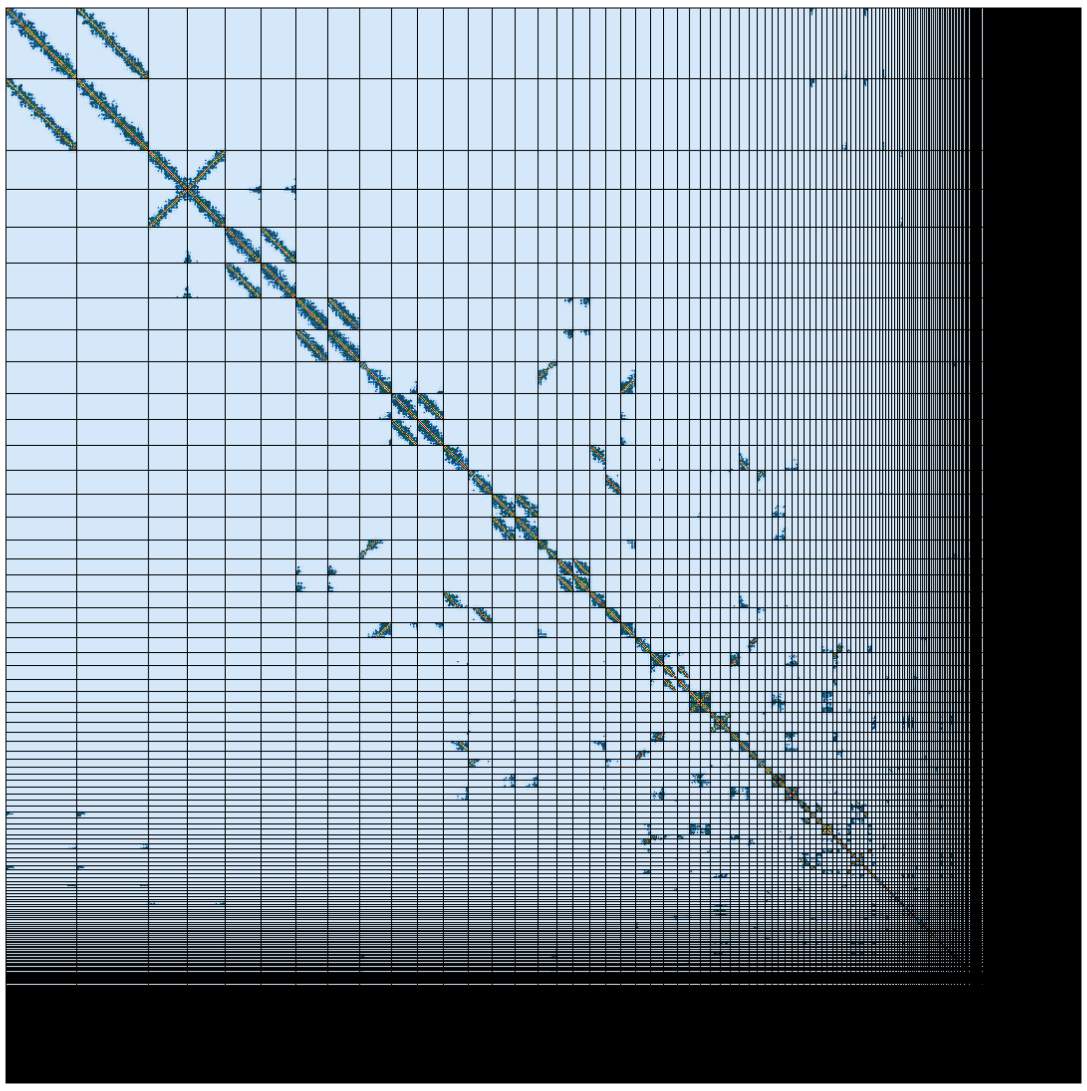

Scapyr__purg HiC Heatmap (assembly 2)

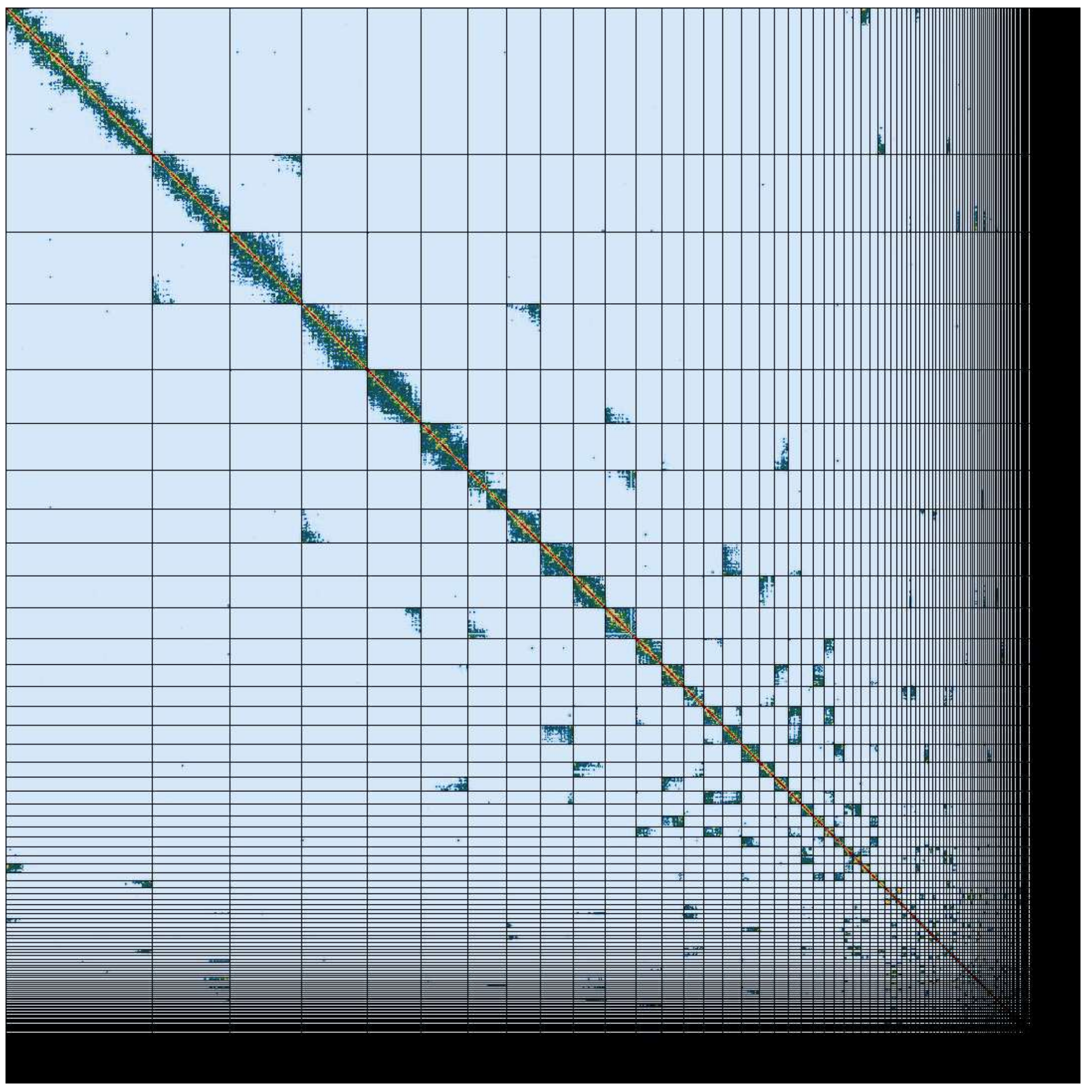

Scapyr__cont HiC Heatmap (assembly 2)

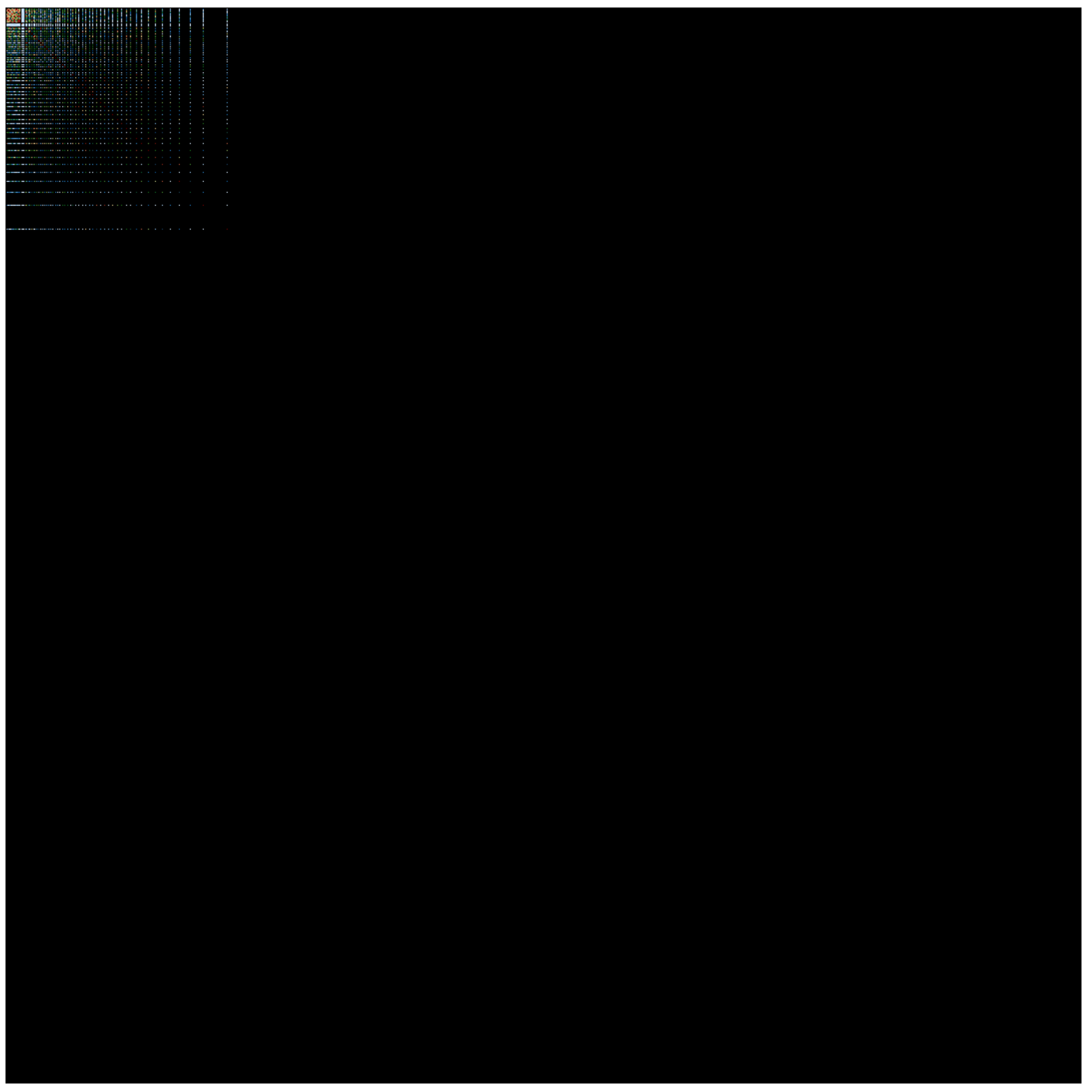

Scapyr__scaf HiC Heatmap (assembly 1)

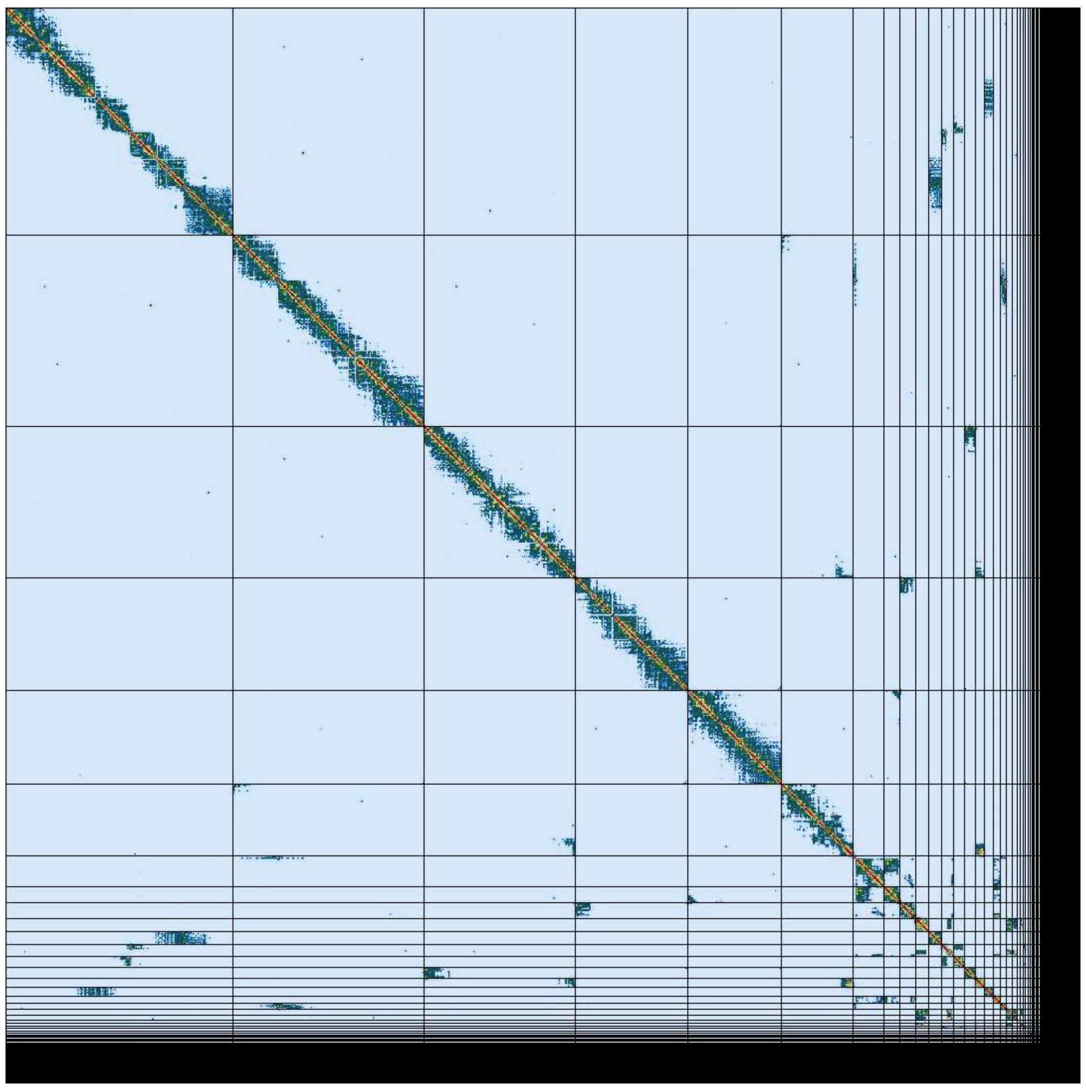

Scapyr__purg HiC Heatmap (assembly 1)

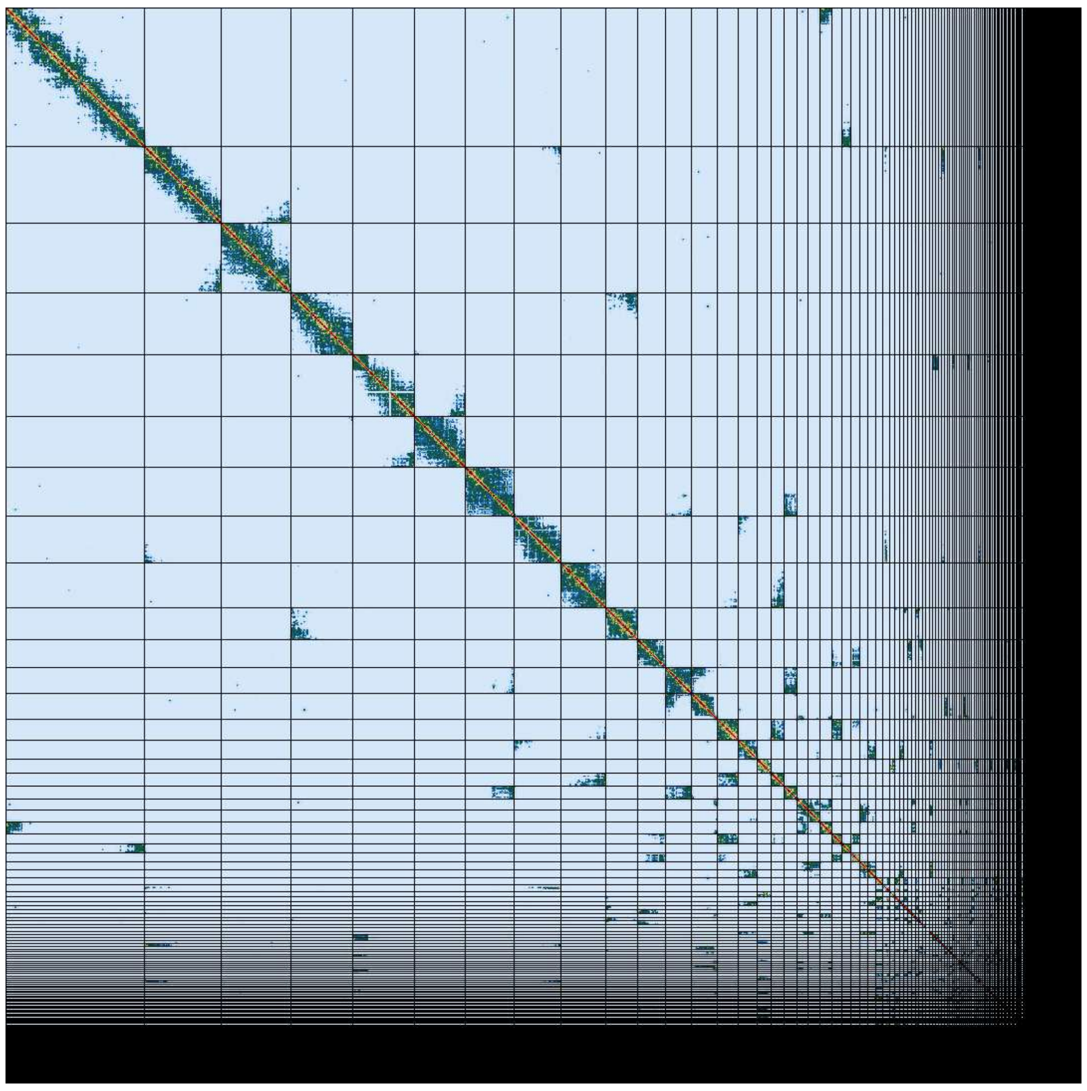

Scapyr__scaf HiC Heatmap (assembly 2)

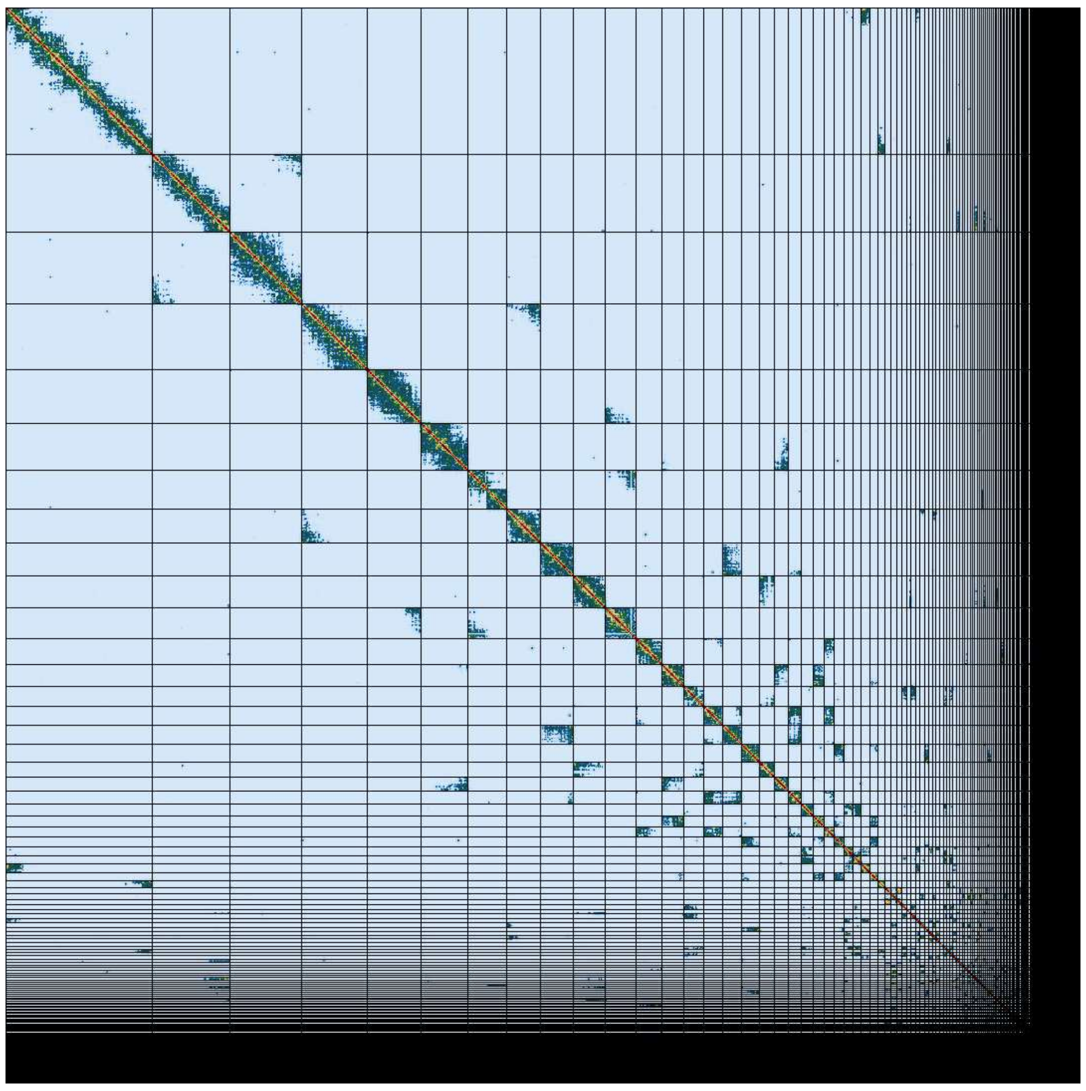

Scapyr__final HiC Heatmap (assembly 1)

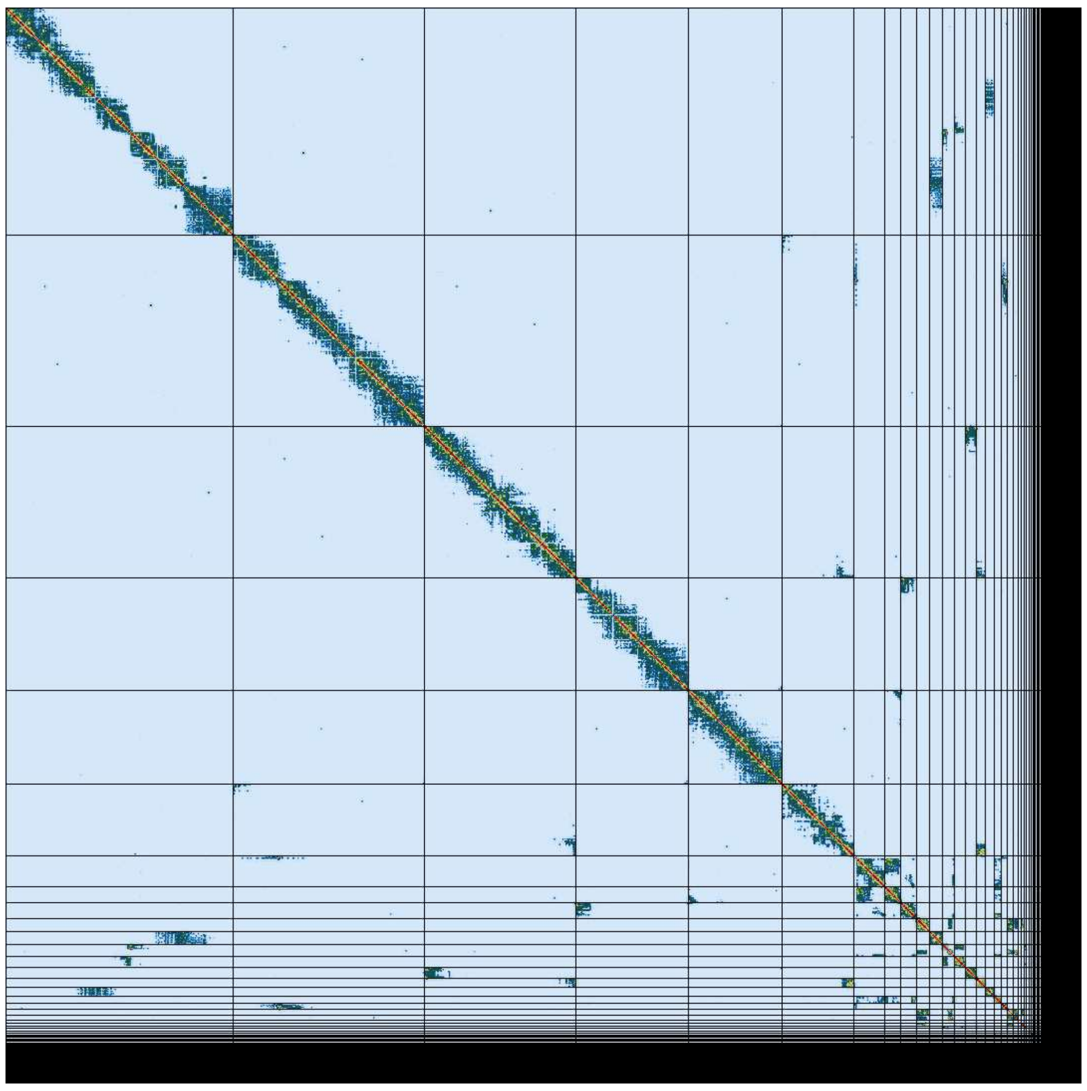

Scapyr__final HiC Heatmap (assembly 2)

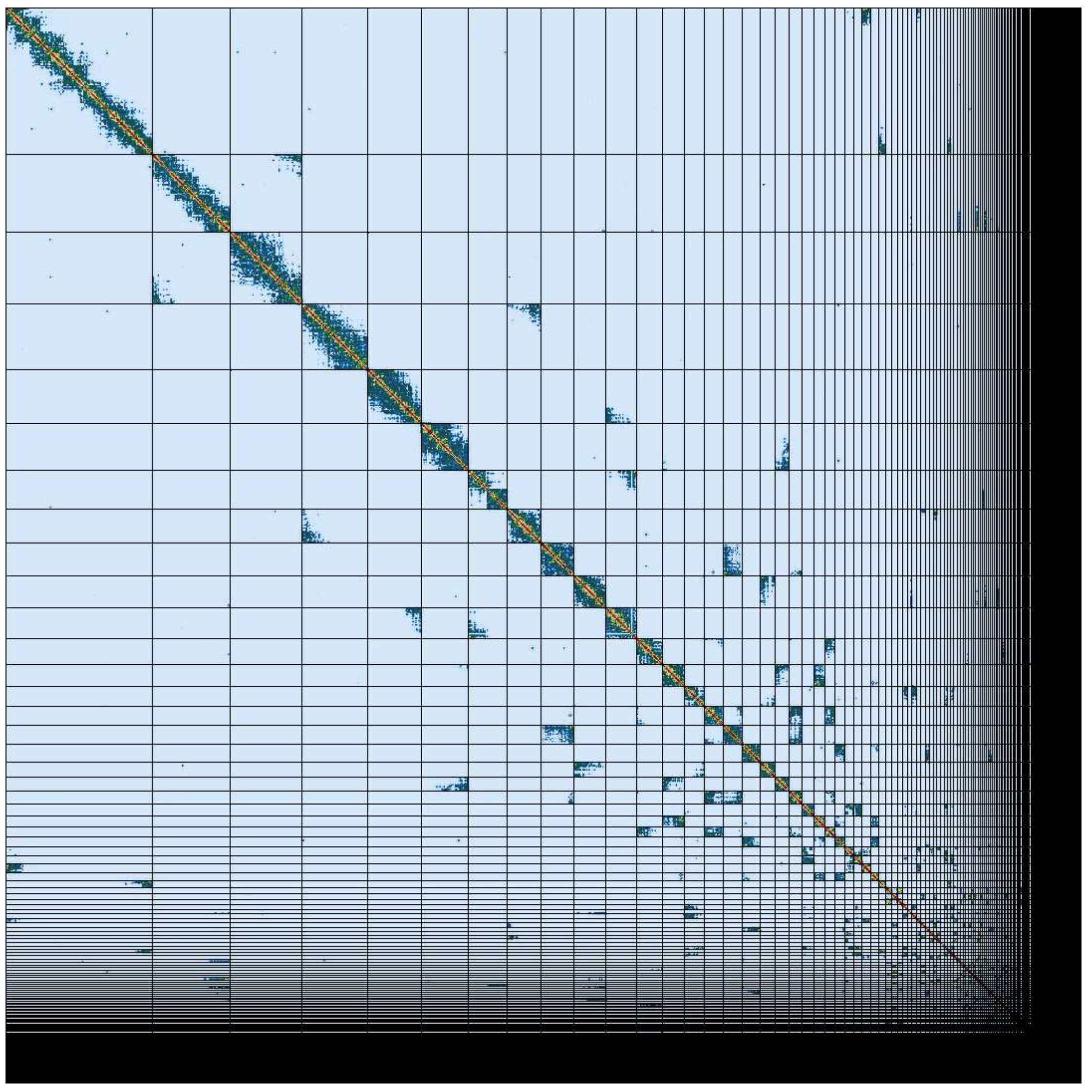

ASM_ID	Total_bp	GC_%	Gaps_per_Gb	Scaf	Cont	Longest_scaf	Scaf_N50	Scaf_L50	Scaf_N90	Longest_cont	Cont_N50	Cont_L50	Cont_N90	qv	Kmer_Compl	Comp_BUSCOs_%	Comp_Single_BUSCOs_%
Scapyr_cont.asm1	661901849	32.78	0	589	589	43965498	11589531	16	687939	43965498	11589531	16	687939	53.8914	98.55950	99.0	1.2
Scapyr_cont.asm2	34141814	32.26	0	1492	1492	491049	23693	522	15237	491049	23693	522	15237	47.8565	3.88778	3.7	2.3
Scapyr_purg.asm1	339508160	32.98	0	290	290	43965498	14568262	8	648476	43965498	14568262	8	648476	52.3474	73.11330	99.1	98.7
Scapyr_purg.asm2	321271535	32.63	0	303	303	43837227	9868577	9	688493	43837227	9868577	9	688493	57.8321	72.75160	98.8	98.2
Scapyr_scaf.asm1	339557660	32.98	291	195	294	71711308	47615032	3	3406514	43965498	14568262	8	644859	52.3474	73.11330	99.2	98.8
Scapyr_scaf.asm2	321271535	32.63	0	303	303	43837227	9868577	9	688493	43837227	9868577	9	688493	57.8321	72.75160	98.8	98.2
Scapyr_final.asm1	339555232	32.98	291	195	294	71711214	47615007	3	3406509	43965472	14568203	8	644858	55.8590	73.11680	99.2	98.8
Scapyr_final.asm2	321270571	32.63	0	303	303	43837241	9868581	9	688493	43837241	9868581	9	688493	58.9201	72.75360	98.8	98.2

Total_bp	GC_%	Gaps_per_Gb	Scaf	Cont	Longest_scaf	Scaf_N50	Scaf_L50	Scaf_N90	Longest_cont	Cont_N50	Cont_L50	Cont_N90	qv	Kmer_Compl	Comp_BUSCOs_%	Comp_Single_BUSCOs_%
Max	Max	Max	Max	Max	Max	Max	Max	Max	Max	Max	Max	Max	Max	Max	Max	Max
Min	Min	Min	Min	Min	Min	Min	Min	Min	Min	Min	Min	Min	Min	Min	Min	Min

ASM_ID	Gaps_per_Gb	Scaf_N50	Cont_N50	qv	Kmer_Compl	Comp_Single_BUSCOs_%
Scapyr_cont.asm1	0	11589531	11589531	53.8914	98.55950	1.2
Scapyr_cont.asm2	0	23693	23693	47.8565	3.88778	2.3
Scapyr_purg.asm1	0	14568262	14568262	52.3474	73.11330	98.7
Scapyr_purg.asm2	0	9868577	9868577	57.8321	72.75160	98.2
Scapyr_scaf.asm1	291	47615032	14568262	52.3474	73.11330	98.8
Scapyr_scaf.asm2	0	9868577	9868577	57.8321	72.75160	98.2
Scapyr_final.asm1	291	47615007	14568203	55.8590	73.11680	98.8
Scapyr_final.asm2	0	9868581	9868581	58.9201	72.75360	98.2

Gaps_per_Gb	Scaf_N50	Cont_N50	qv	Kmer_Compl	Comp_Single_BUSCOs_%
< 200	> 100Mbp	> 10Mbp	> 50	> 95%	> 95%
200 - 1000	10Mbp - 100Mbp	1Mbp - 10Mbp	40 - 50	90% - 95%	90% - 95%
1000 - 10000	0.1Mbp - 10Mbp	0.01Mbp - 1Mbp	35 - 40	80% - 90%	80% - 90%
> 10000	< 0.1Mbp	< 0.01Mbp	< 35	< 80%	< 80%

Case 2b: Outcomes of contigging parameters tweaking

Assembly ID: Scapyr_10

Database built with kmer size: 21 bp

Number of haploptypes/assemblies: 2

Expected genome size: 300901319

Heterozygosity: 2.322 %

QV Score

assembly_name	Assembly_Only	Total_Kmers	QV_Score
Scapyr_10.asm1	88677	667060224	51.9855
Scapyr_10.asm2	111735	157722256	44.7178
Both	200412	824782480	49.3658

Kmer Completeness

assembly_name	Kmers_Assembly	Kmers_Reads	%
Scapyr_10.asm1	359306787	364489292	98.5781
Scapyr_10.asm2	85814344	364489292	23.5437
both	359325722	364489292	98.5833

BUSCOv5 (database: insecta)

Metric	asm1 #	asm1 %	asm2 #	asm2 %
Complete	1355	99.2	378	99.2
Complete and single-copy	20	1.5	320	1.5
Complete and duplicated	1335	97.7	58	97.7
Fragmented	1	0.1	30	0.1
Missing	11	0.7	959	0.7
Total searched groups	1367	1367.0	1367	1367.0

Genomescope2 Profile (Linear)

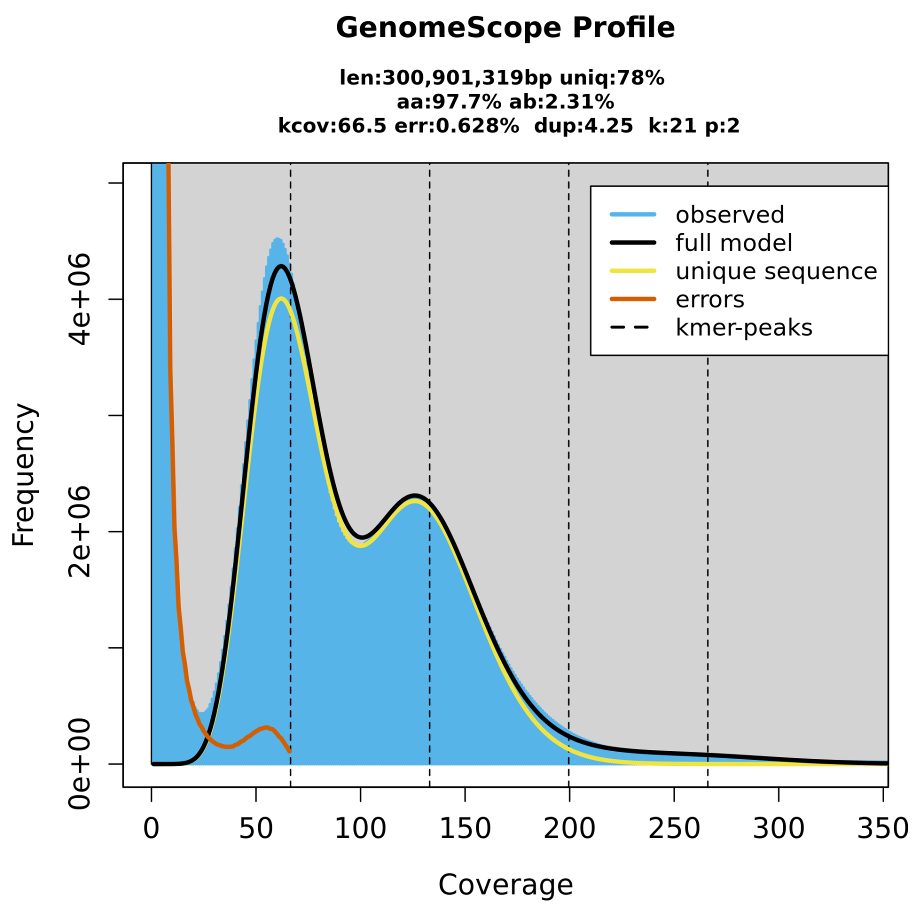

Smudgeplot Profile

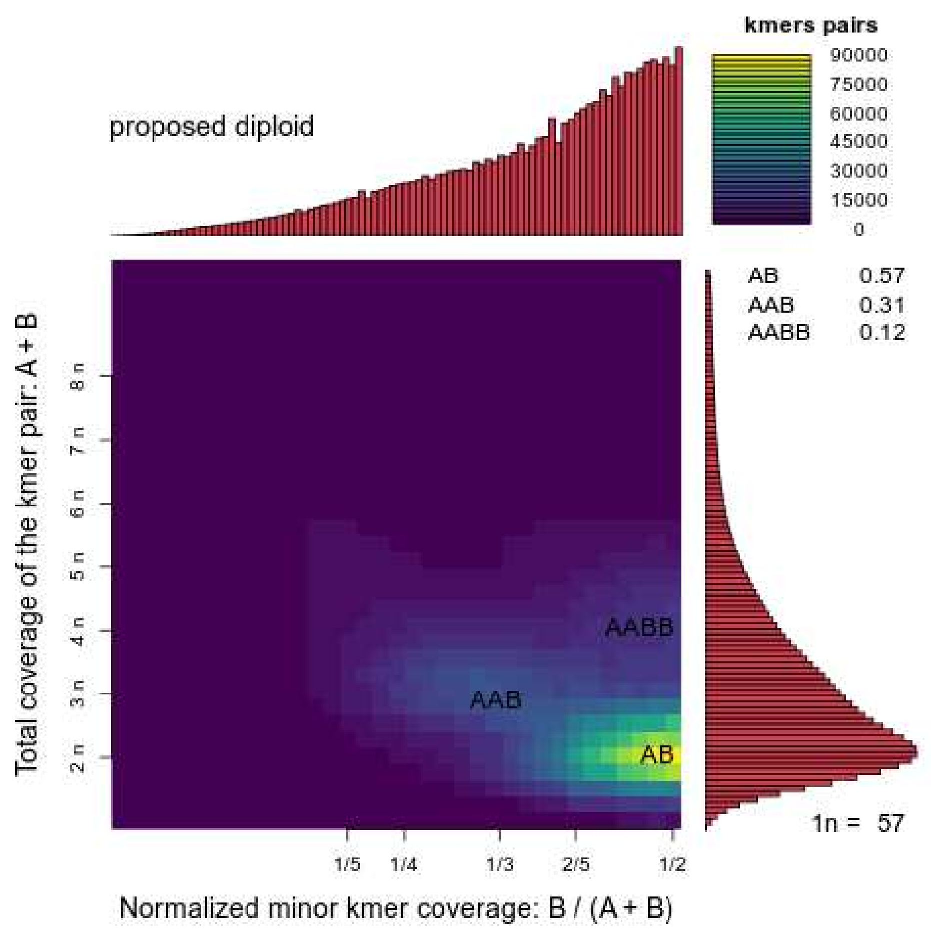

Genome Statistics with gfastats

Metric	asm1	asm2
Total scaffold length	667072864	157865476
GC content %	32.80	32.49
# gaps in scaffolds	0	0
Total gap length in scaffolds	0	0
# scaffolds	632	7161
Largest scaffold	45470904	270827
Scaffold N50	13479259	22430
Scaffold L50	16	2924
Scaffold L90	99	6076
# contigs	632	7161
Largest contig	45470904	270827
Contig N50	13479259	22430
Contig L50	16	2924
Contig L90	99	6076

K-mer Multiplicity asm1 Only (Flat)

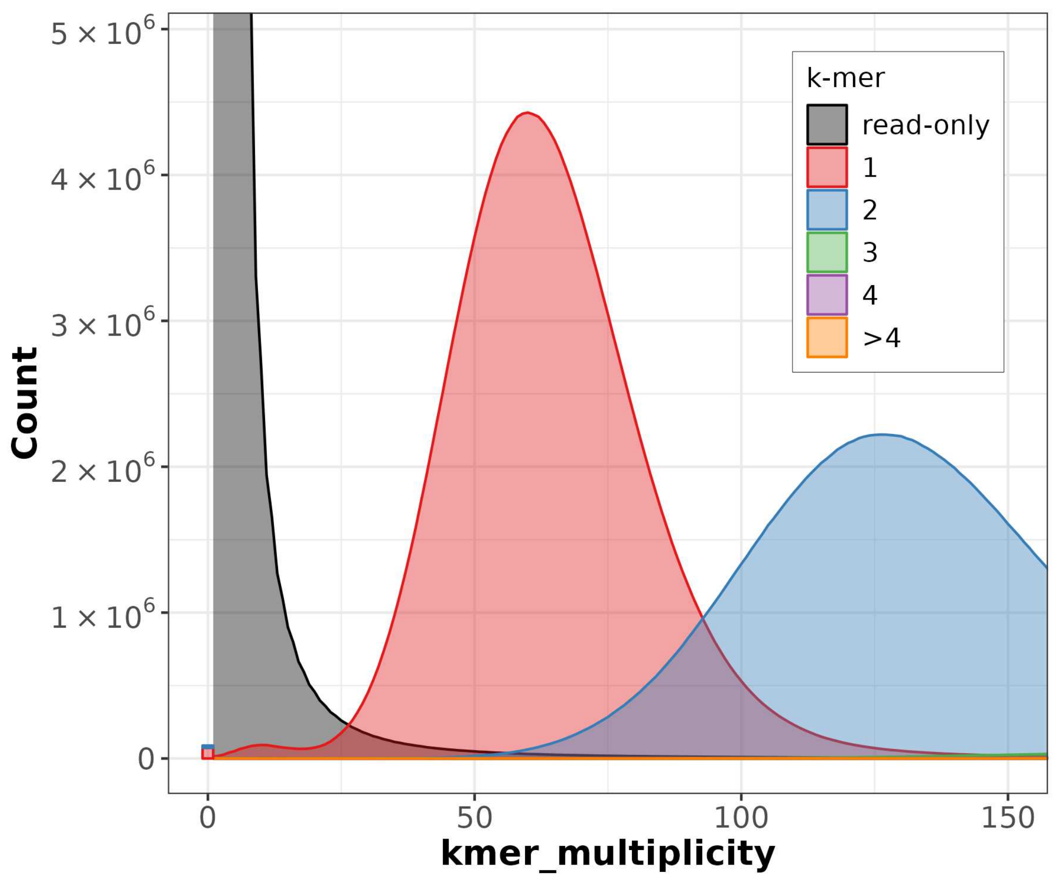

K-mer Multiplicity asm1 + asm2 (Flat)

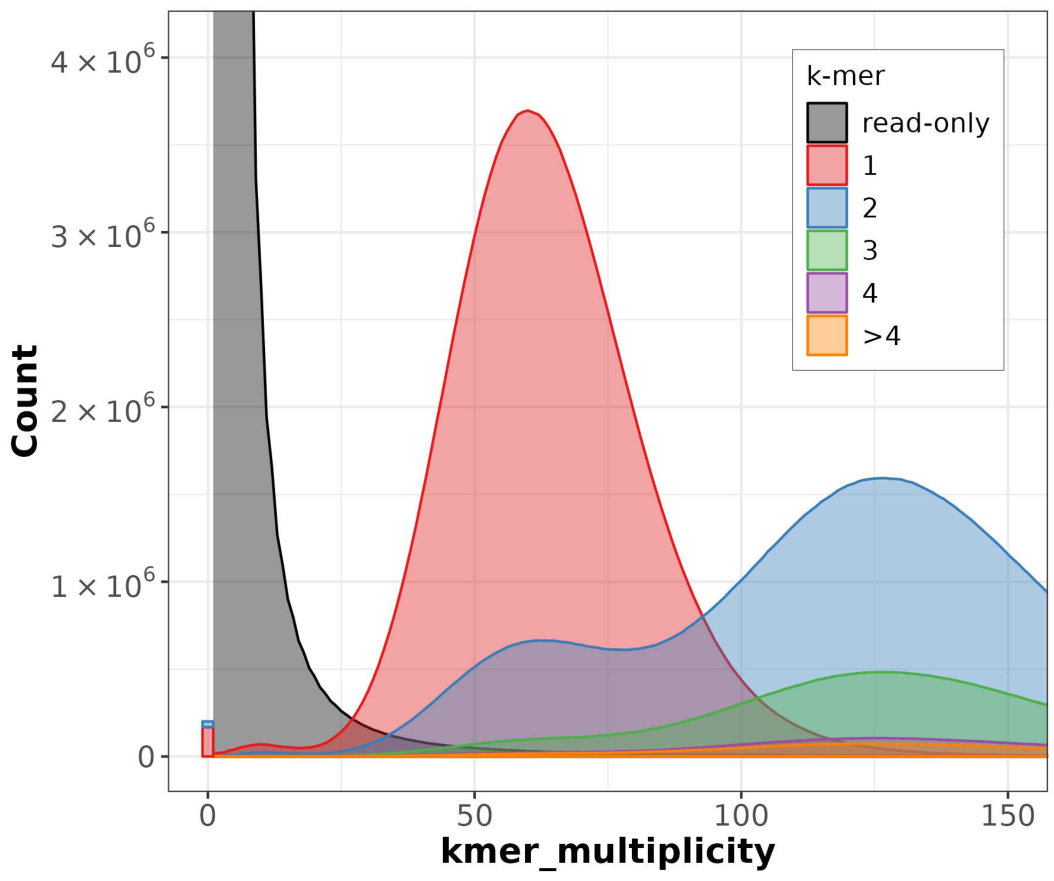

Assembly ID: Scapyr__10__hic

Database built with kmer size: 21 bp

Number of haploptypes/assemblies: 2

Expected genome size: 300901319

Heterozygosity: 2.322 %

QV Score

assembly_name	Assembly_Only	Total_Kmers	QV_Score
Scapyr__10__hic.asm1	57874	561994754	53.0945
Scapyr__10__hic.asm2	45725	552189606	54.0414
Both	103599	1114184360	53.538

Kmer Completeness

assembly_name	Kmers_Assembly	Kmers_Reads	%
Scapyr__10__hic.asm1	330736146	364489292	90.7396
Scapyr__10__hic.asm2	330615597	364489292	90.7065
both	359315706	364489292	98.5806

BUSCOv5 (database: insecta)

Metric	asm1 #	asm1 %	asm2 #	asm2 %
Complete	1355	99.1	1355	99.1
Complete and single-copy	260	19.0	282	19.0
Complete and duplicated	1095	80.1	1073	80.1
Fragmented	1	0.1	1	0.1
Missing	11	0.8	11	0.8
Total searched groups	1367	1367.0	1367	1367.0

Genomescope2 Profile (Linear)

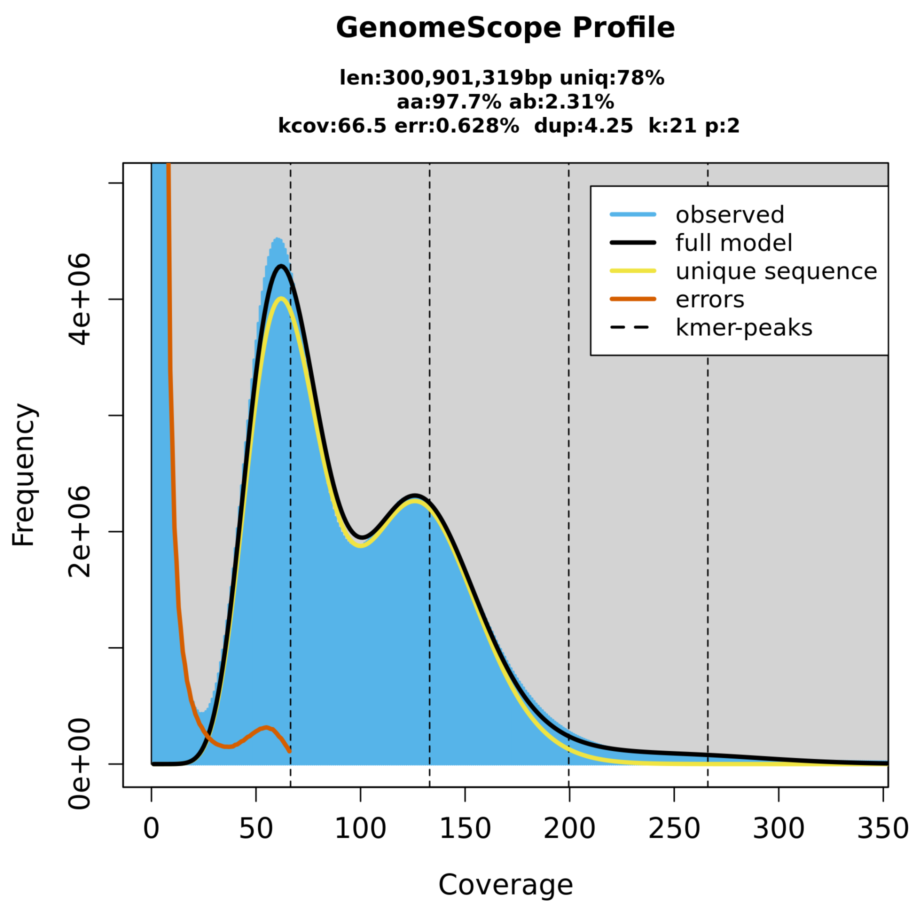

Smudgeplot Profile

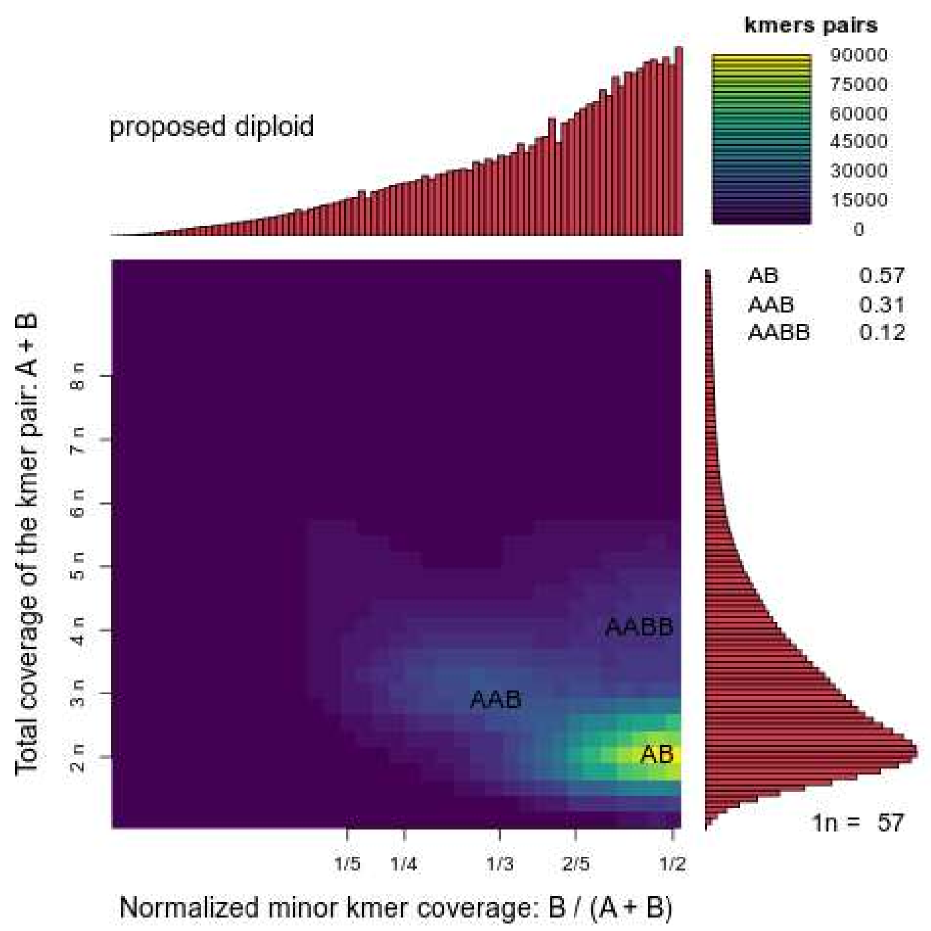

Genome Statistics with gfastats

Metric	asm1	asm2
Total scaffold length	562006054	552197406
GC content %	32.73	32.75
# gaps in scaffolds	0	0
Total gap length in scaffolds	0	0
# scaffolds	565	390
Largest scaffold	45426467	43841759
Scaffold N50	7728830	6998311
Scaffold L50	18	19
Scaffold L90	109	102
# contigs	565	390
Largest contig	45426467	43841759
Contig N50	7728830	6998311
Contig L50	18	19
Contig L90	109	102

K-mer Multiplicity asm1 Only (Flat)

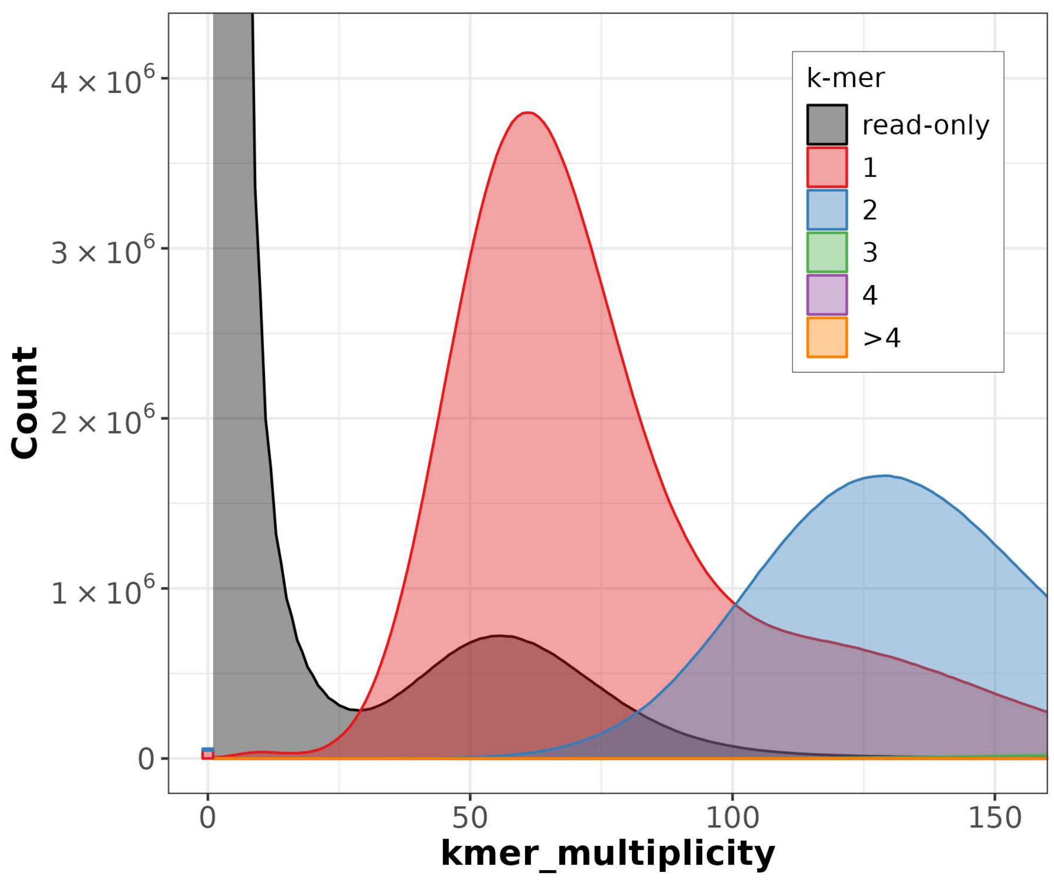

K-mer Multiplicity asm1 + asm2 (Flat)

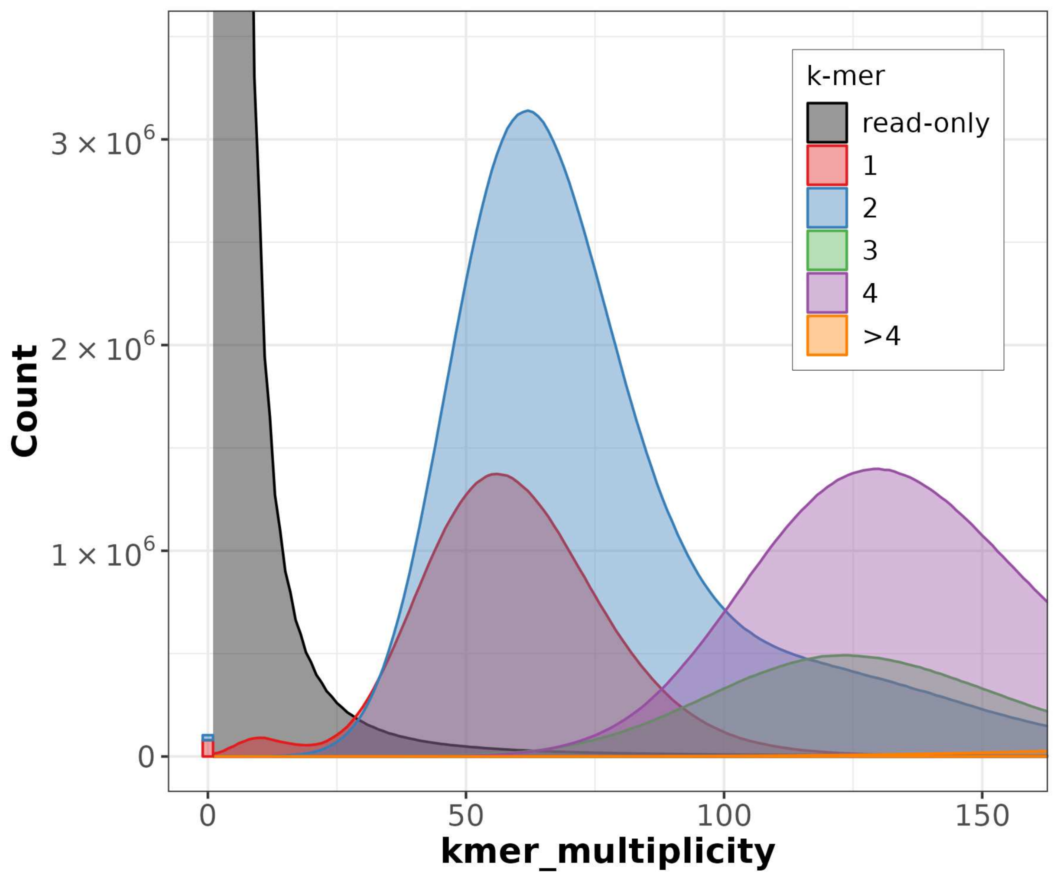

Assembly ID: Scapyr_11

Database built with kmer size: 21 bp

Number of haploptypes/assemblies: 2

Expected genome size: 300901319

Heterozygosity: 2.322 %

QV Score

assembly_name	Assembly_Only	Total_Kmers	QV_Score
Scapyr__11.asm1	68783	447093647	51.351
Scapyr__11.asm2	128835	394896535	48.086
Both	197618	841990182	49.5165

Kmer Completeness

assembly_name	Kmers_Assembly	Kmers_Reads	%
Scapyr__11.asm1	293876685	364489292	80.627
Scapyr__11.asm2	218460841	364489292	59.9361
both	359325502	364489292	98.5833

BUSCOv5 (database: insecta)

Metric	asm1 #	asm1 %	asm2 #	asm2 %
Complete	1354	99.0	998	99.0
Complete and single-copy	837	61.2	776	61.2
Complete and duplicated	517	37.8	222	37.8
Fragmented	1	0.1	18	0.1
Missing	12	0.9	351	0.9
Total searched groups	1367	1367.0	1367	1367.0

Genomescope2 Profile (Linear)

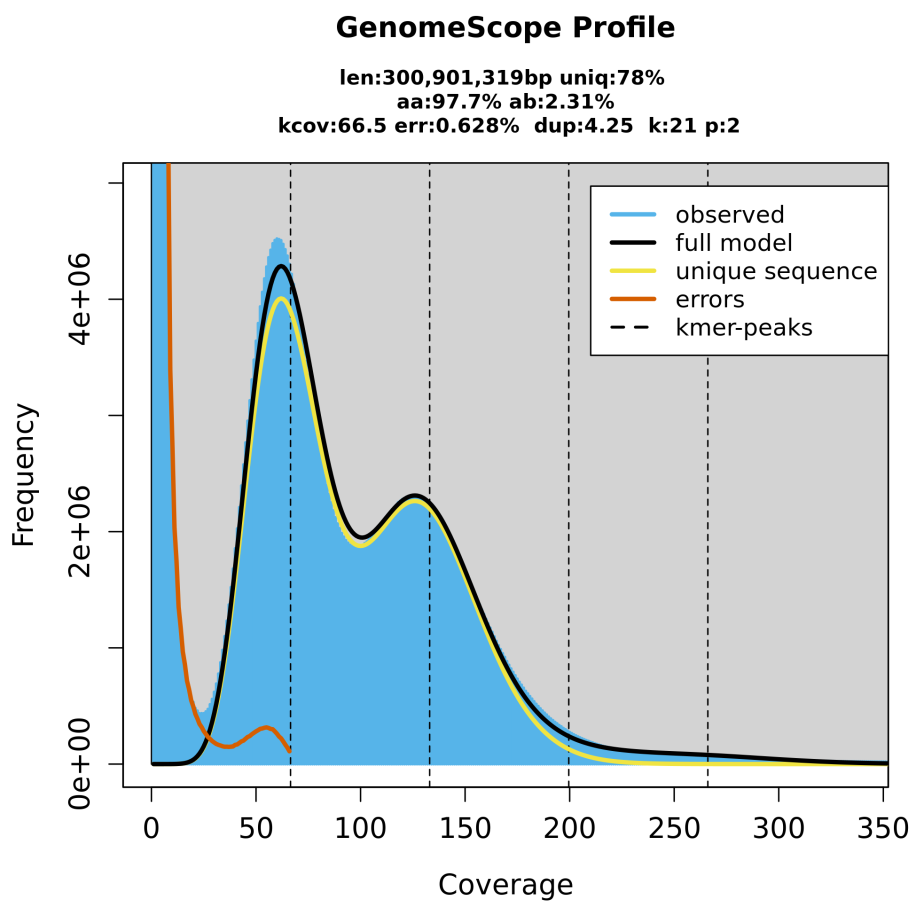

Smudgeplot Profile

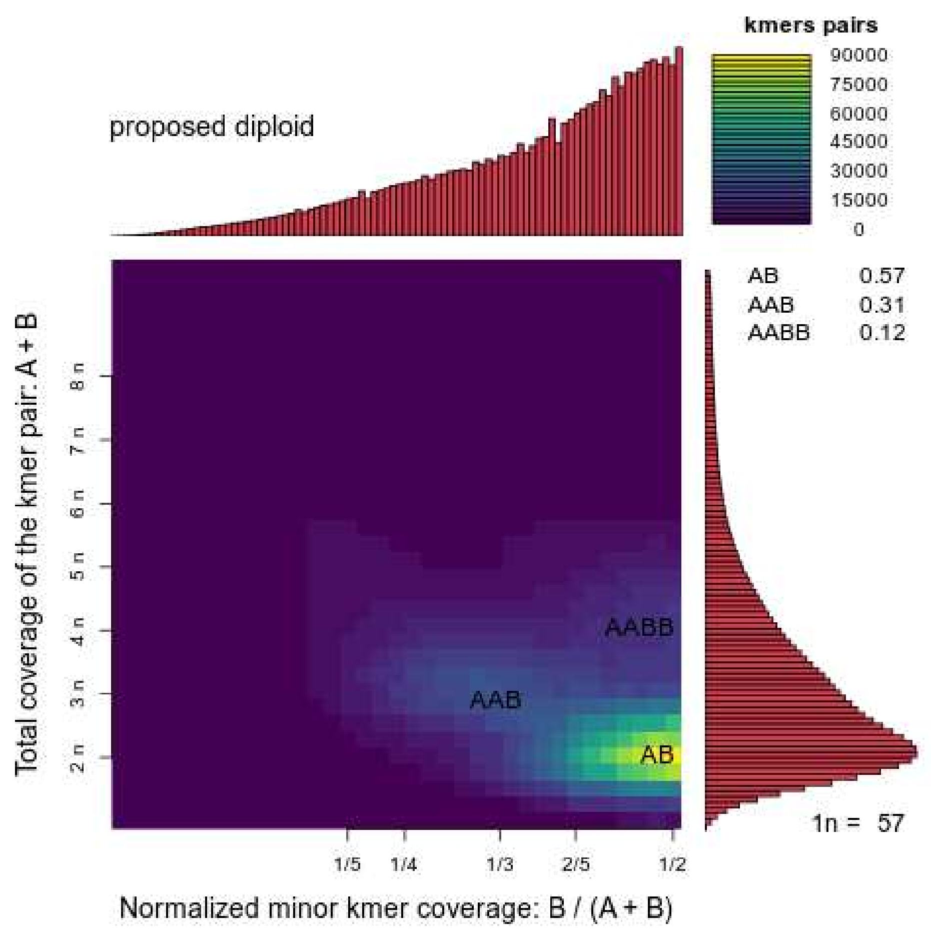

Genome Statistics with gfastats

Metric	asm1	asm2
Total scaffold length	447102587	395073435
GC content %	32.74	32.75
# gaps in scaffolds	0	0
Total gap length in scaffolds	0	0
# scaffolds	447	8845
Largest scaffold	45470852	1003927
Scaffold N50	11602984	90830
Scaffold L50	12	910
Scaffold L90	68	6527
# contigs	447	8845
Largest contig	45470852	1003927
Contig N50	11602984	90830
Contig L50	12	910
Contig L90	68	6527

K-mer Multiplicity asm1 Only (Flat)

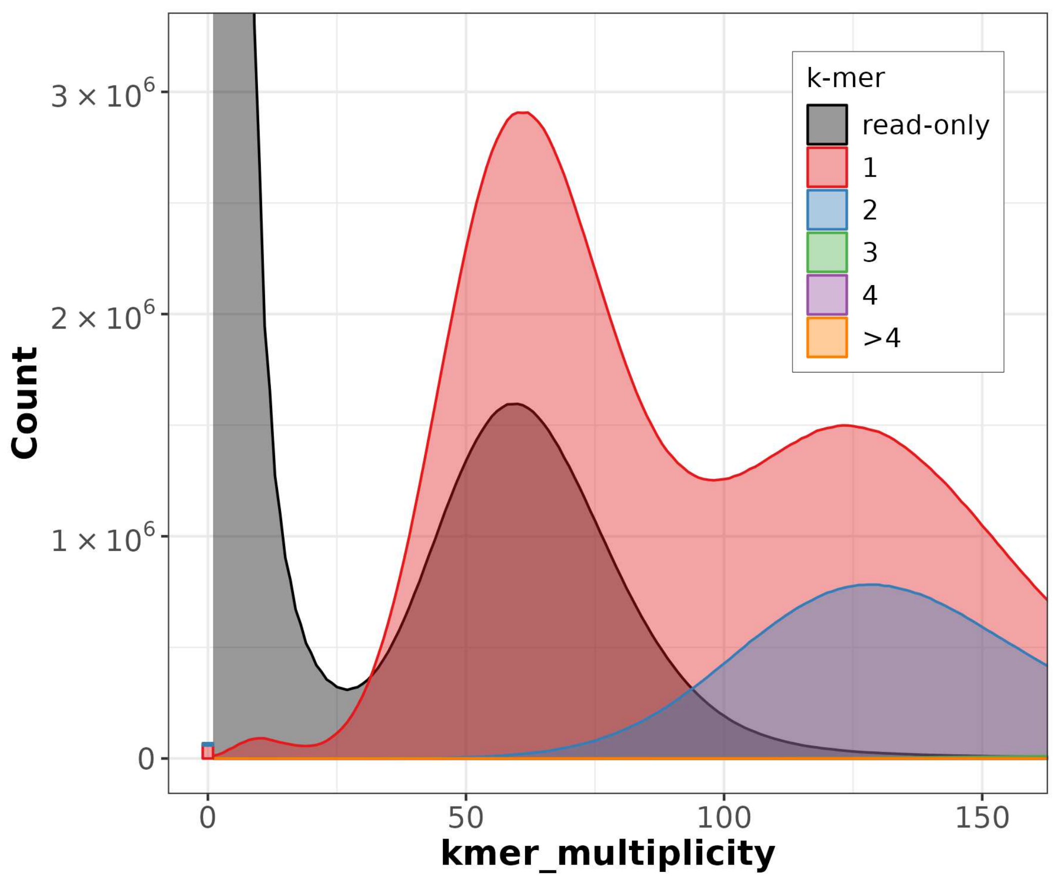

K-mer Multiplicity asm1 + asm2 (Flat)

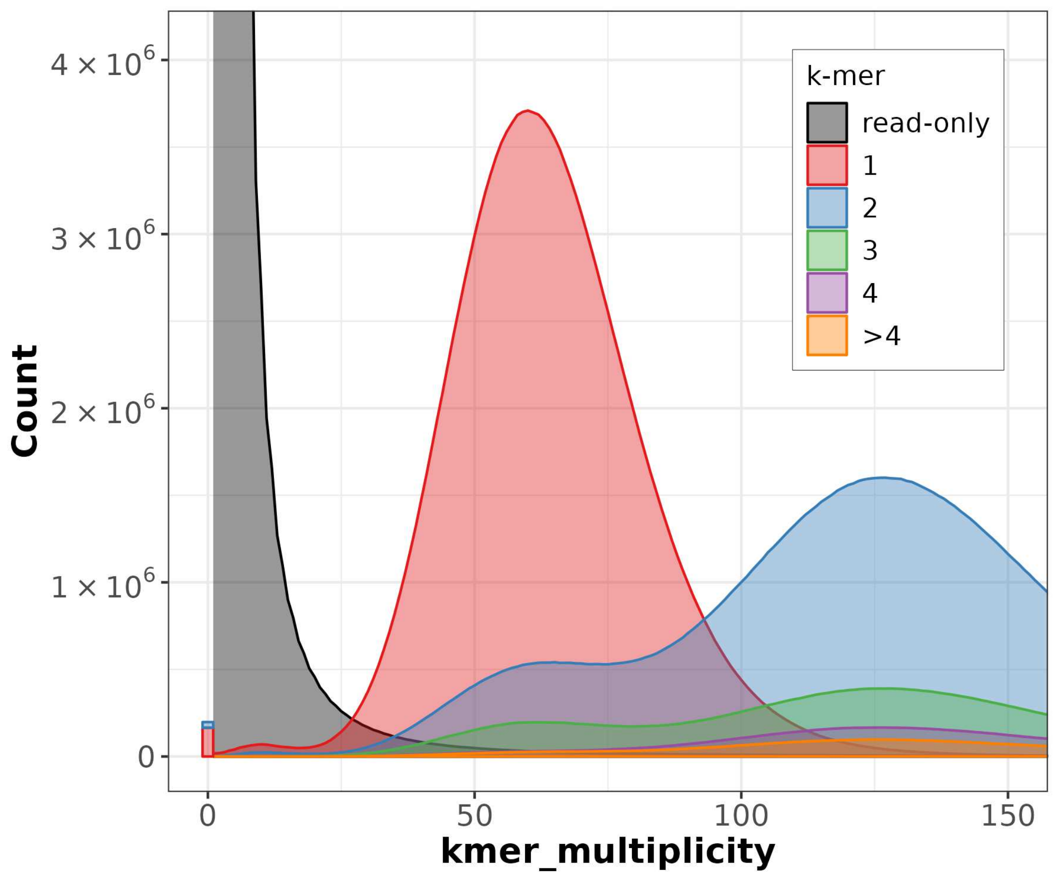

Assembly ID: Scapyr__11__hic

Database built with kmer size: 21 bp

Number of haploptypes/assemblies: 2

Expected genome size: 300901319

Heterozygosity: 2.322 %

QV Score

assembly_name	Assembly_Only	Total_Kmers	QV_Score
Scapyr__11__hic.asm1	62110	339517618	50.5988
Scapyr__11__hic.asm2	19974	331899405	55.4275
Both	82084	671417023	52.3493

Kmer Completeness

assembly_name	Kmers_Assembly	Kmers_Reads	%
Scapyr__11__hic.asm1	266584055	364489292	73.1391
Scapyr__11__hic.asm2	266669992	364489292	73.1626
both	359304168	364489292	98.5774

BUSCOv5 (database: insecta)

Metric	asm1 #	asm1 %	asm2 #	asm2 %
Complete	1353	99.0	1353	99.0
Complete and single-copy	1348	98.6	1345	98.6
Complete and duplicated	5	0.4	8	0.4
Fragmented	2	0.1	1	0.1
Missing	12	0.9	13	0.9
Total searched groups	1367	1367.0	1367	1367.0

Genomescope2 Profile (Linear)

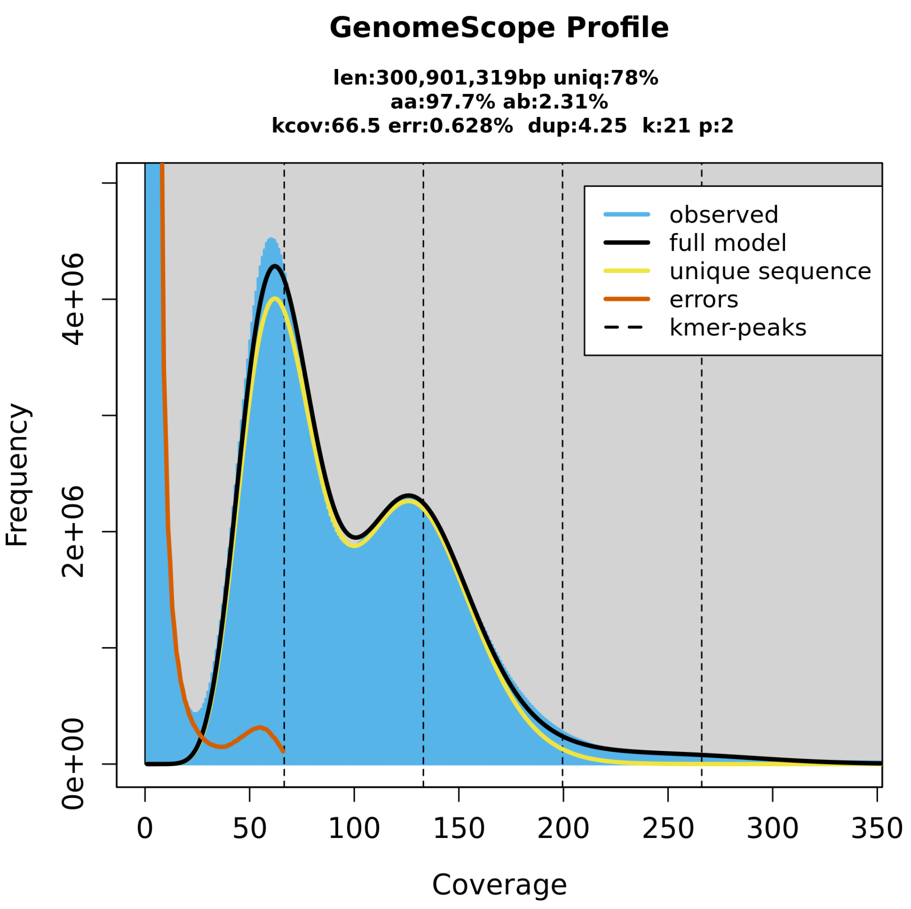

Smudgeplot Profile

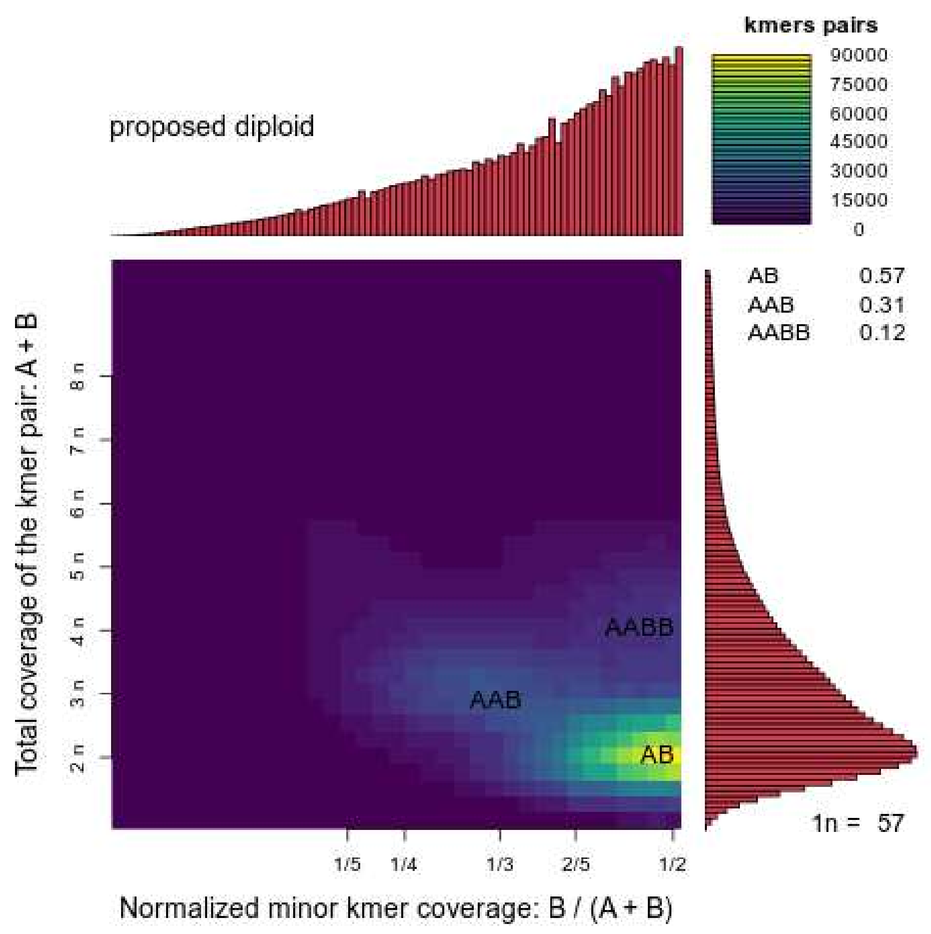

Genome Statistics with gfastats

Metric	asm1	asm2
Total scaffold length	339525858	331904165
GC content %	32.80	32.75
# gaps in scaffolds	0	0
Total gap length in scaffolds	0	0
# scaffolds	412	238
Largest scaffold	24357256	43973524
Scaffold N50	14822151	11603031
Scaffold L50	9	8
Scaffold L90	55	49
# contigs	412	238
Largest contig	24357256	43973524
Contig N50	14822151	11603031
Contig L50	9	8
Contig L90	55	49

K-mer Multiplicity asm1 Only (Flat)

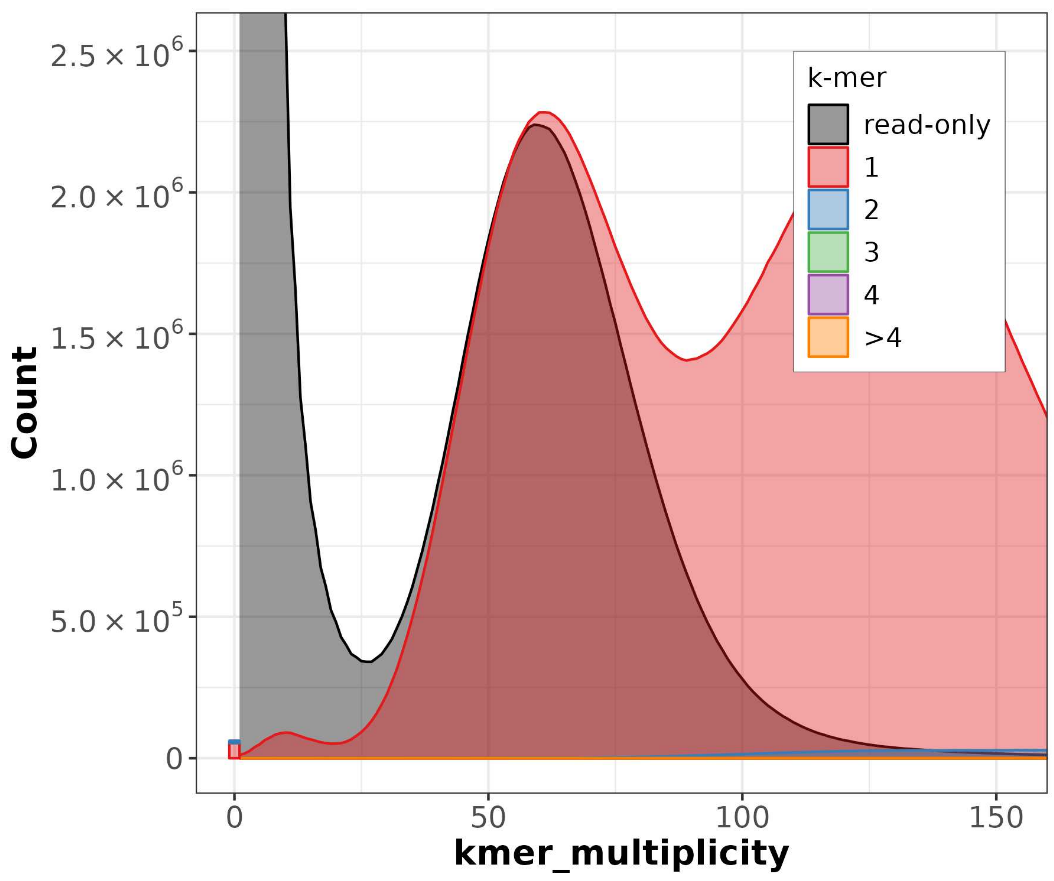

K-mer Multiplicity asm1 + asm2 (Flat)

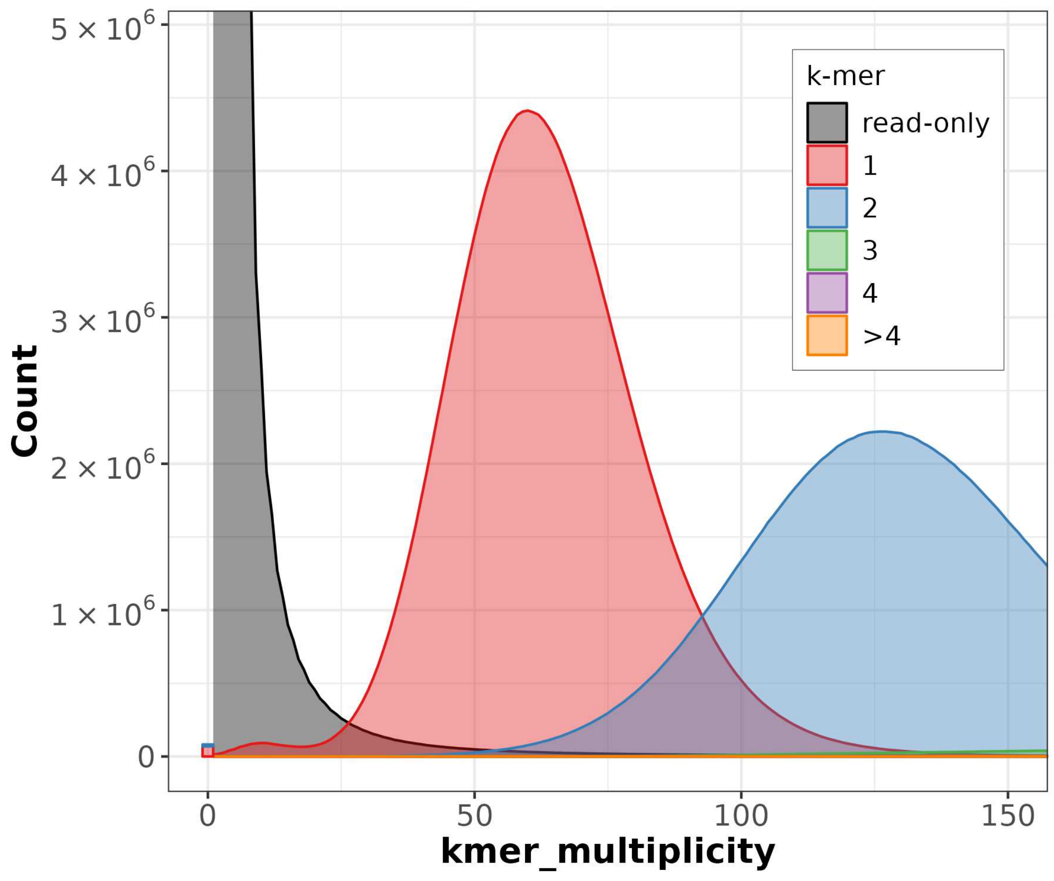

Assembly ID: Scapyr_12

Database built with kmer size: 21 bp

Number of haploptypes/assemblies: 2

Expected genome size: 300901319

Heterozygosity: 2.322 %

QV Score

assembly_name	Assembly_Only	Total_Kmers	QV_Score
Scapyr_12.asm1	59595	338448243	50.7647
Scapyr_12.asm2	139862	517855730	48.9067
Both	199457	856303973	49.5495

Kmer Completeness

assembly_name	Kmers_Assembly	Kmers_Reads	%
Scapyr_12.asm1	266487871	364489292	73.1127
Scapyr_12.asm2	281370230	364489292	77.1957
both	359325488	364489292	98.5833

BUSCOv5 (database: insecta)

Metric	asm1 #	asm1 %	asm2 #	asm2 %
Complete	1352	98.9	1350	98.9
Complete and single-copy	1347	98.5	1013	98.5
Complete and duplicated	5	0.4	337	0.4
Fragmented	1	0.1	4	0.1
Missing	14	1.0	13	1.0
Total searched groups	1367	1367.0	1367	1367.0

Genomescope2 Profile (Linear)

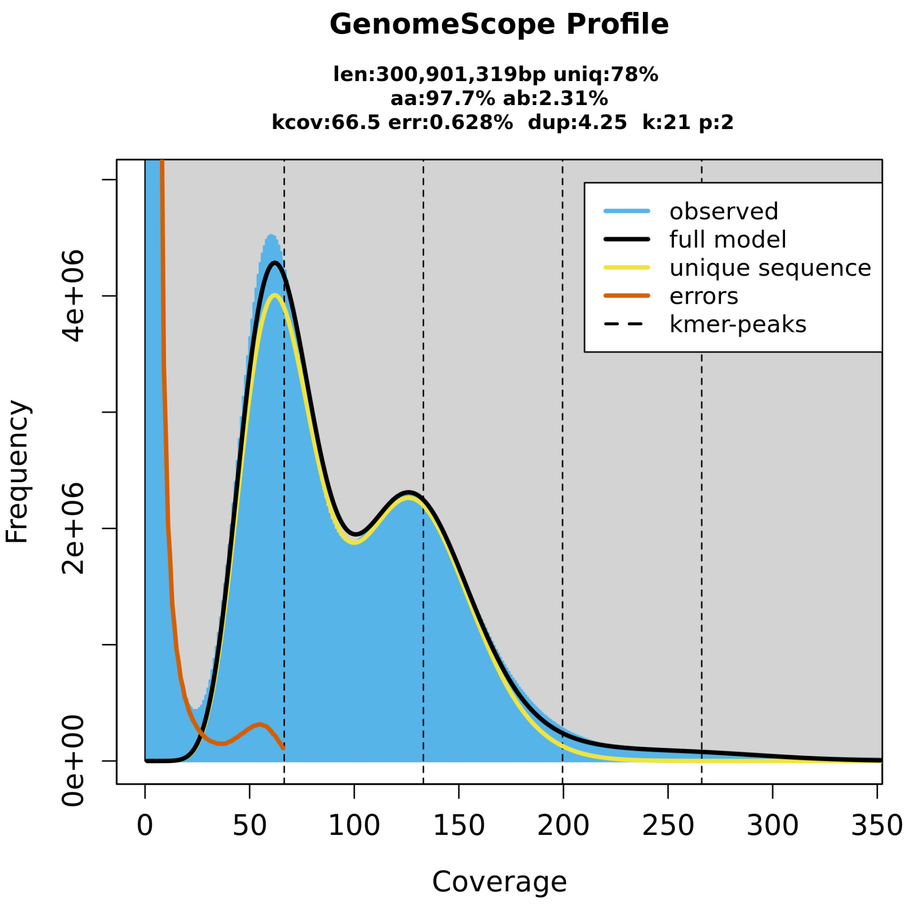

Smudgeplot Profile

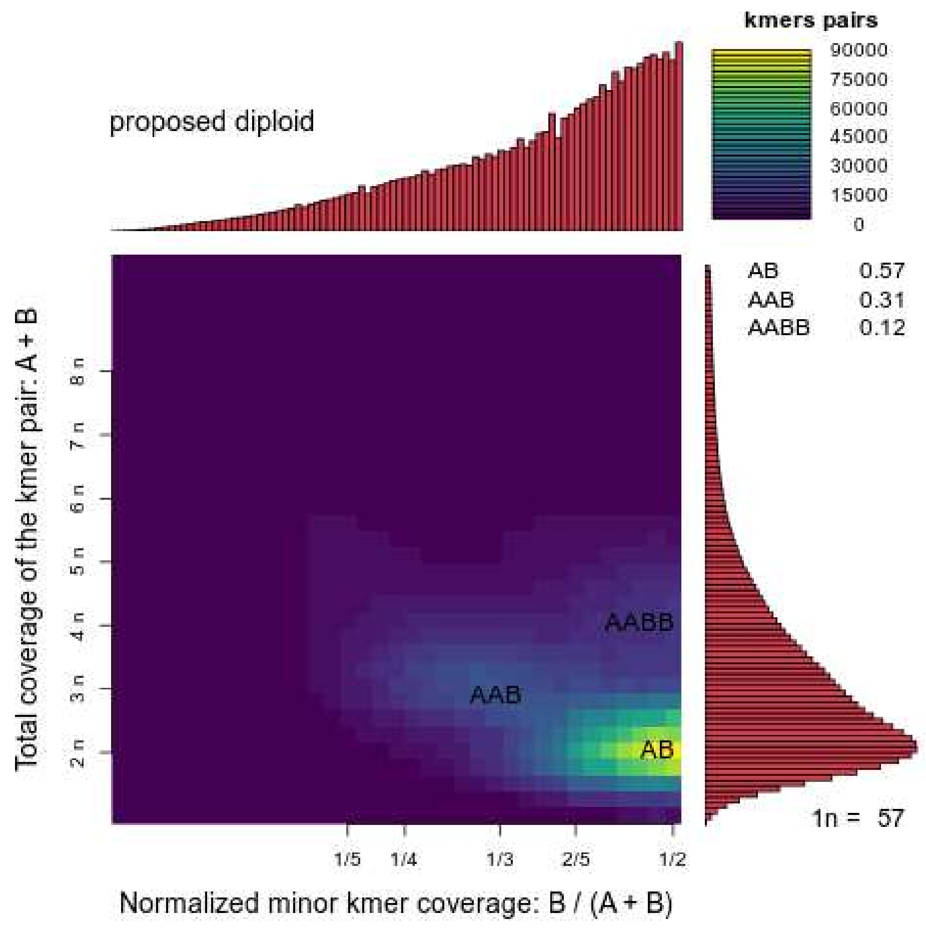

Genome Statistics with gfastats

Metric	asm1	asm2
Total scaffold length	338455103	518055150
GC content %	32.74	32.74
# gaps in scaffolds	0	0
Total gap length in scaffolds	0	0
# scaffolds	343	9971
Largest scaffold	46943257	1003927
Scaffold N50	14912490	124705
Scaffold L50	7	1023
Scaffold L90	38	7010
# contigs	343	9971
Largest contig	46943257	1003927
Contig N50	14912490	124705
Contig L50	7	1023
Contig L90	38	7010

K-mer Multiplicity asm1 Only (Flat)

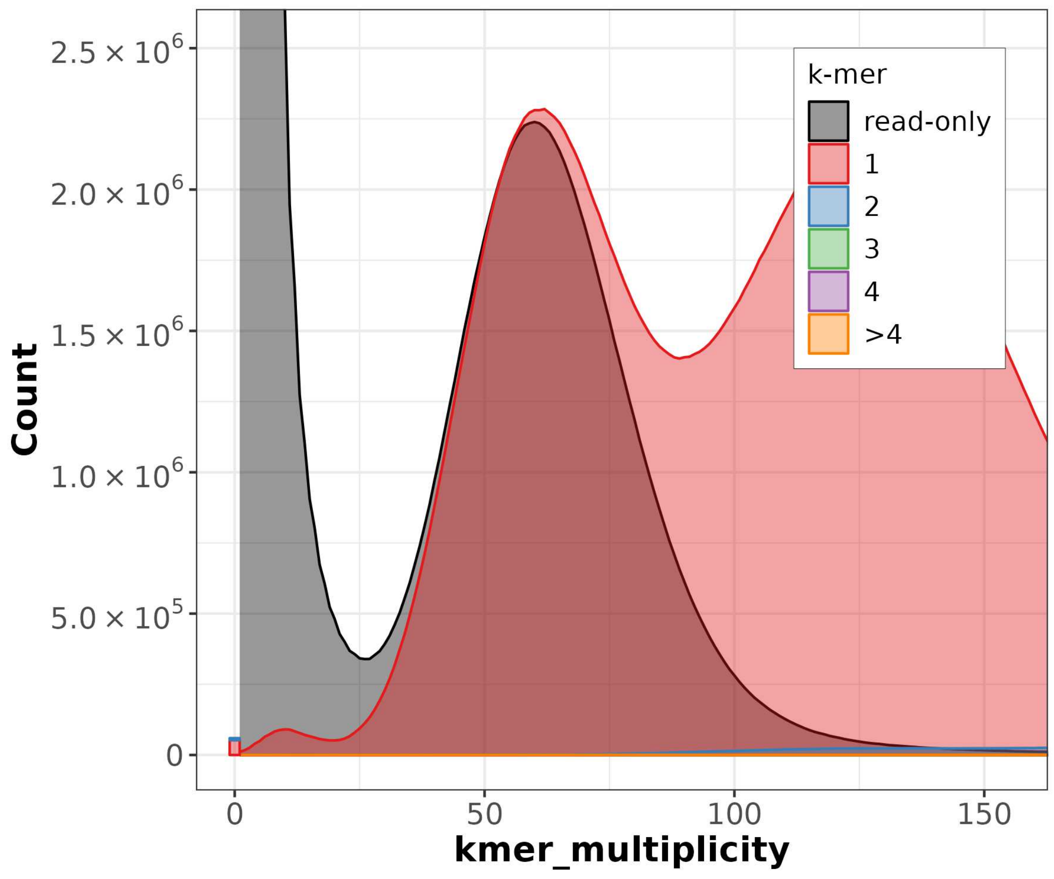

K-mer Multiplicity asm1 + asm2 (Flat)

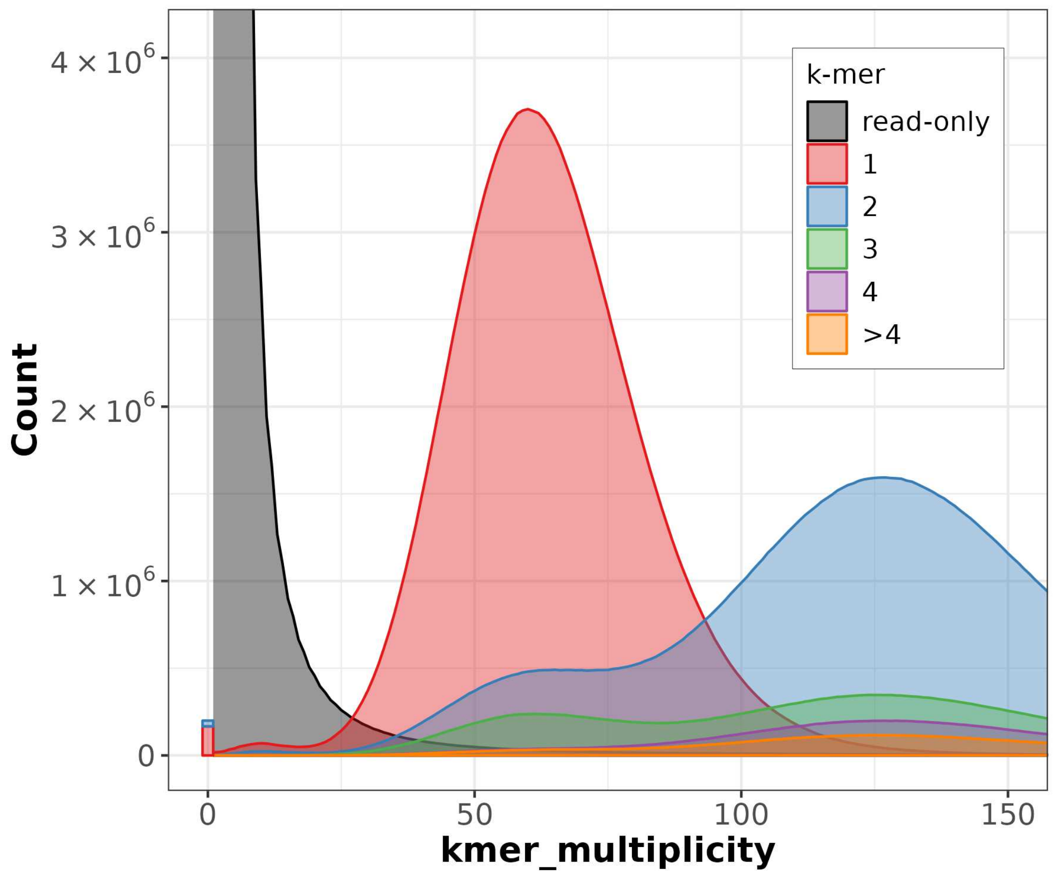

Assembly ID: Scapyr__12__hic

Database built with kmer size: 21 bp

Number of haploptypes/assemblies: 2

Expected genome size: 300901319

Heterozygosity: 2.322 %

QV Score

assembly_name	Assembly_Only	Total_Kmers	QV_Score
Scapyr__12__hic.asm1	69294	342411506	50.1603
Scapyr__12__hic.asm2	21140	333235291	55.1985
Both	90434	675646797	51.9558

Kmer Completeness

assembly_name	Kmers_Assembly	Kmers_Reads	%
Scapyr__12__hic.asm1	266644153	364489292	73.1556
Scapyr__12__hic.asm2	266717517	364489292	73.1757
both	359315219	364489292	98.5805

BUSCOv5 (database: insecta)

Metric	asm1 #	asm1 %	asm2 #	asm2 %
Complete	1353	99.0	1353	99.0
Complete and single-copy	1344	98.3	1344	98.3
Complete and duplicated	9	0.7	9	0.7
Fragmented	2	0.1	1	0.1
Missing	12	0.9	13	0.9
Total searched groups	1367	1367.0	1367	1367.0

Genomescope2 Profile (Linear)

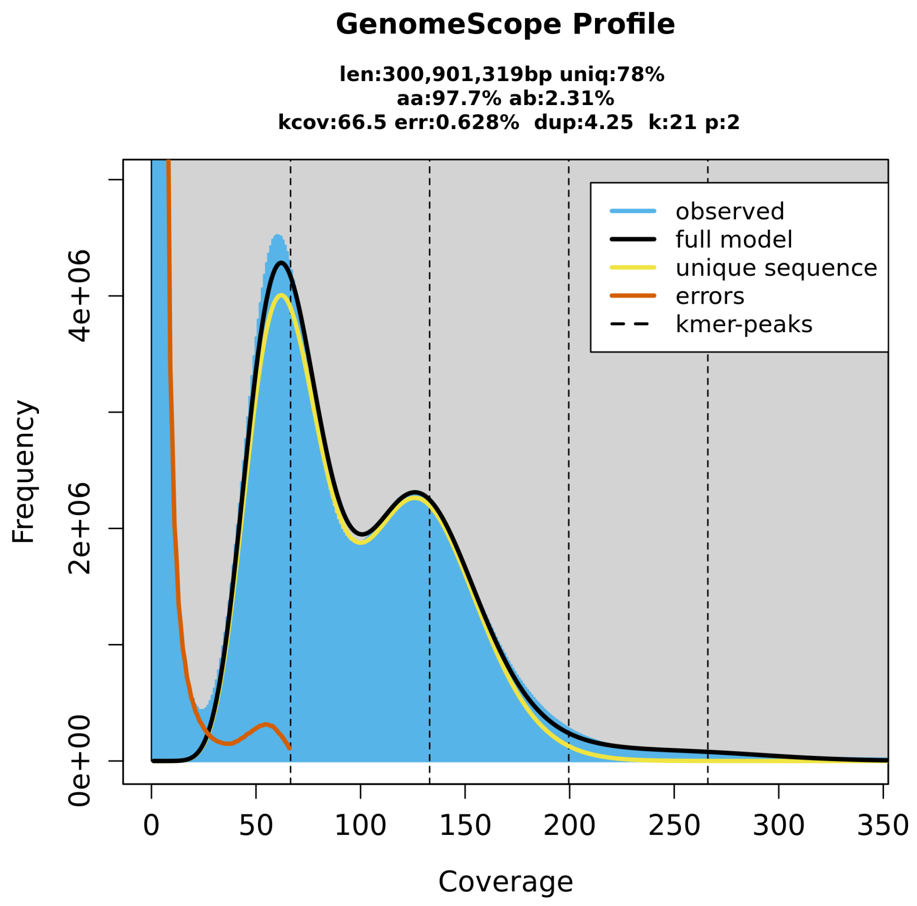

Smudgeplot Profile

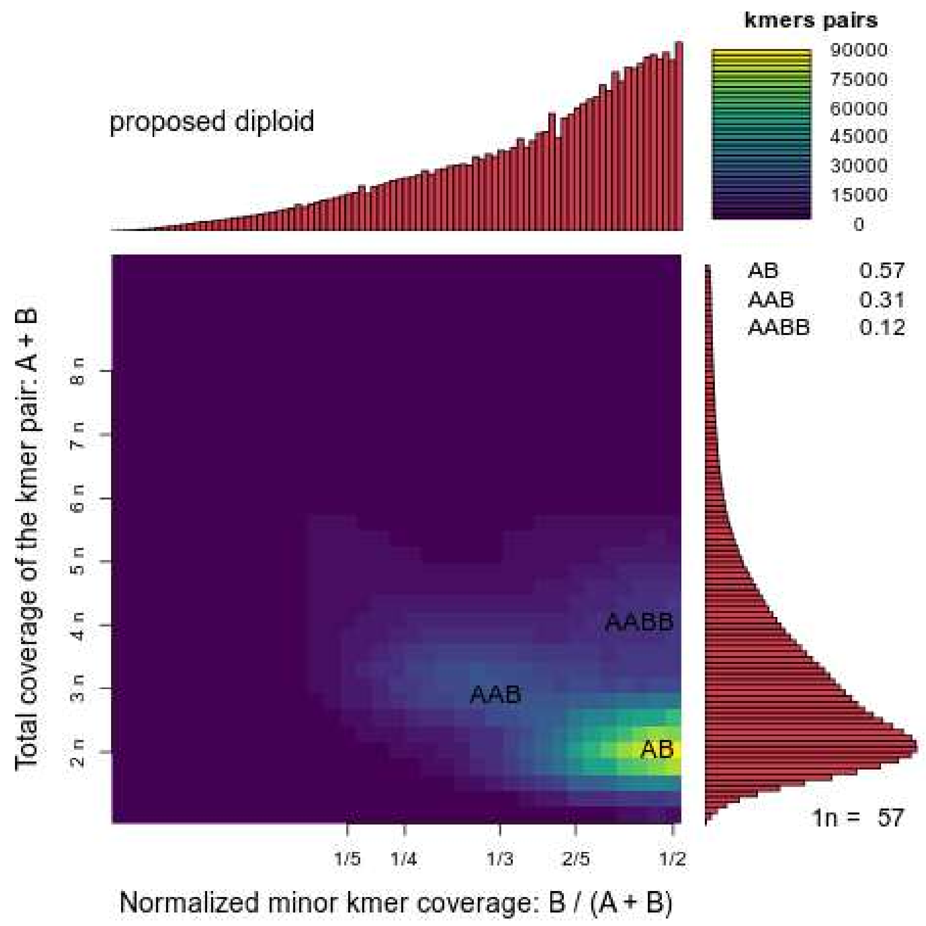

Genome Statistics with gfastats

Metric	asm1	asm2
Total scaffold length	342421346	333240511
GC content %	32.83	32.76
# gaps in scaffolds	0	0
Total gap length in scaffolds	0	0
# scaffolds	492	261
Largest scaffold	24357256	43973510
Scaffold N50	14822151	11603031
Scaffold L50	9	8
Scaffold L90	59	50
# contigs	492	261
Largest contig	24357256	43973510
Contig N50	14822151	11603031
Contig L50	9	8
Contig L90	59	50

K-mer Multiplicity asm1 Only (Flat)

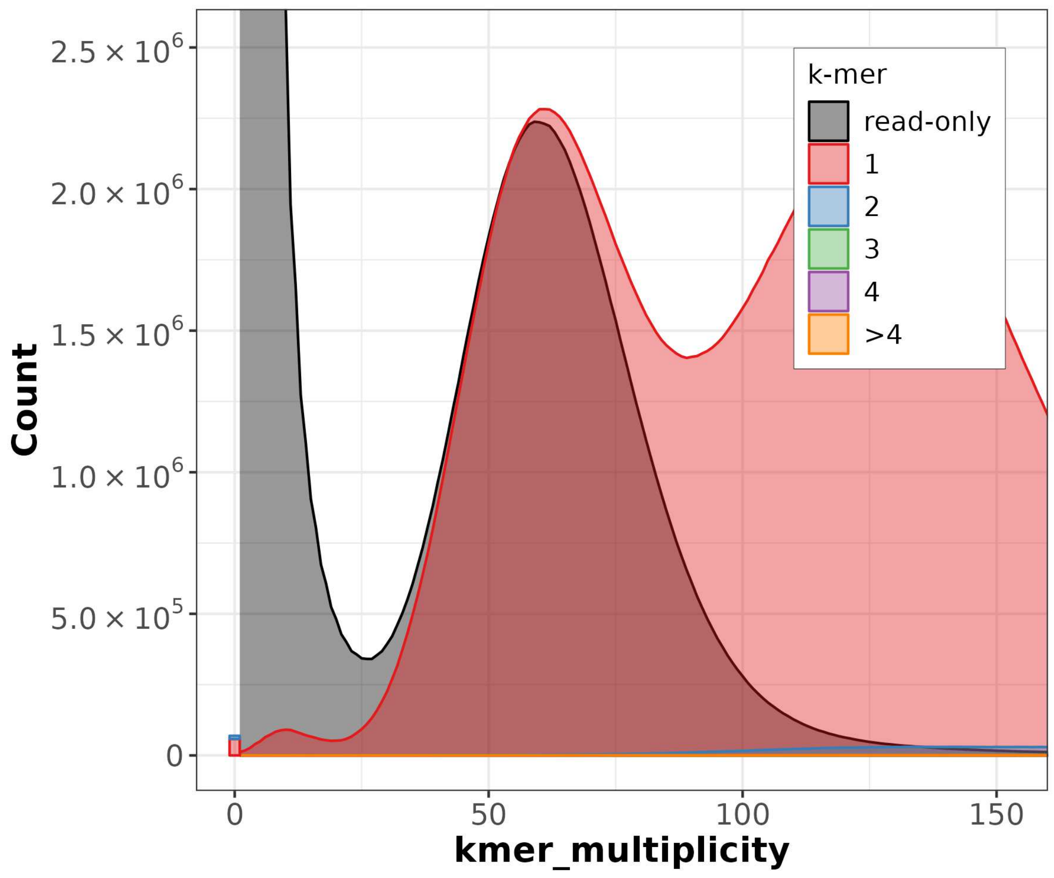

K-mer Multiplicity asm1 + asm2 (Flat)

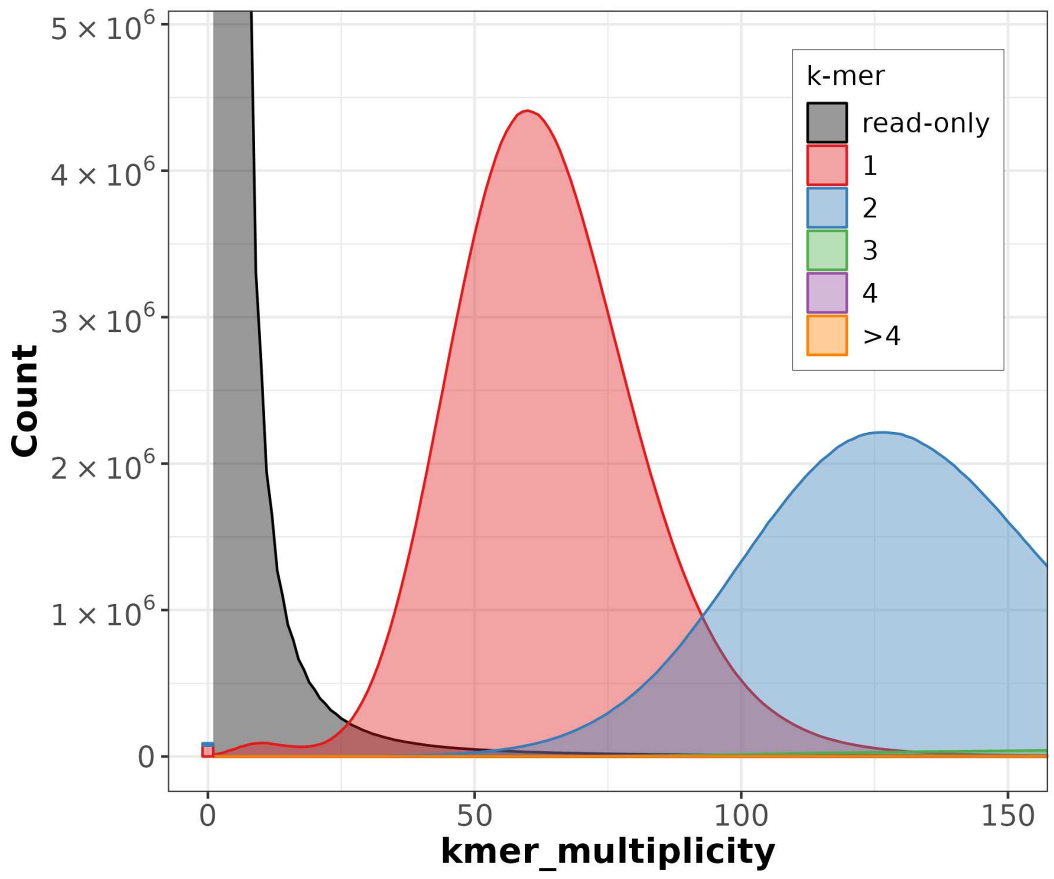

Assembly ID: Scapyr_13

Database built with kmer size: 21 bp

Number of haploptypes/assemblies: 2

Expected genome size: 300901319

Heterozygosity: 2.322 %

QV Score

assembly_name	Assembly_Only	Total_Kmers	QV_Score
Scapyr_13.asm1	57455	334531500	50.8729
Scapyr_13.asm2	142524	522421736	48.863
Both	199979	856953236	49.5414

Kmer Completeness

assembly_name	Kmers_Assembly	Kmers_Reads	%
Scapyr_13.asm1	265106720	364489292	72.7337
Scapyr_13.asm2	281889354	364489292	77.3382
both	359325387	364489292	98.5832

BUSCOv5 (database: insecta)

Metric	asm1 #	asm1 %	asm2 #	asm2 %
Complete	1343	98.3	1350	98.3
Complete and single-copy	1338	97.9	1006	97.9
Complete and duplicated	5	0.4	344	0.4
Fragmented	1	0.1	4	0.1
Missing	23	1.6	13	1.6
Total searched groups	1367	1367.0	1367	1367.0

Genomescope2 Profile (Linear)

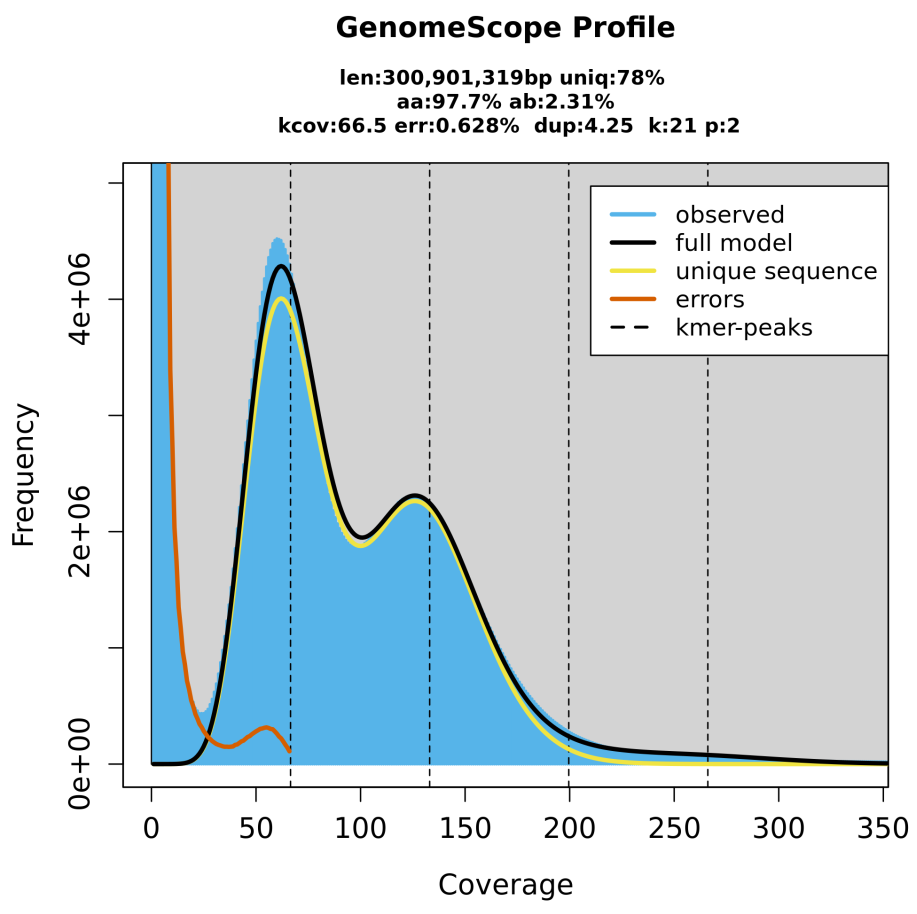

Smudgeplot Profile

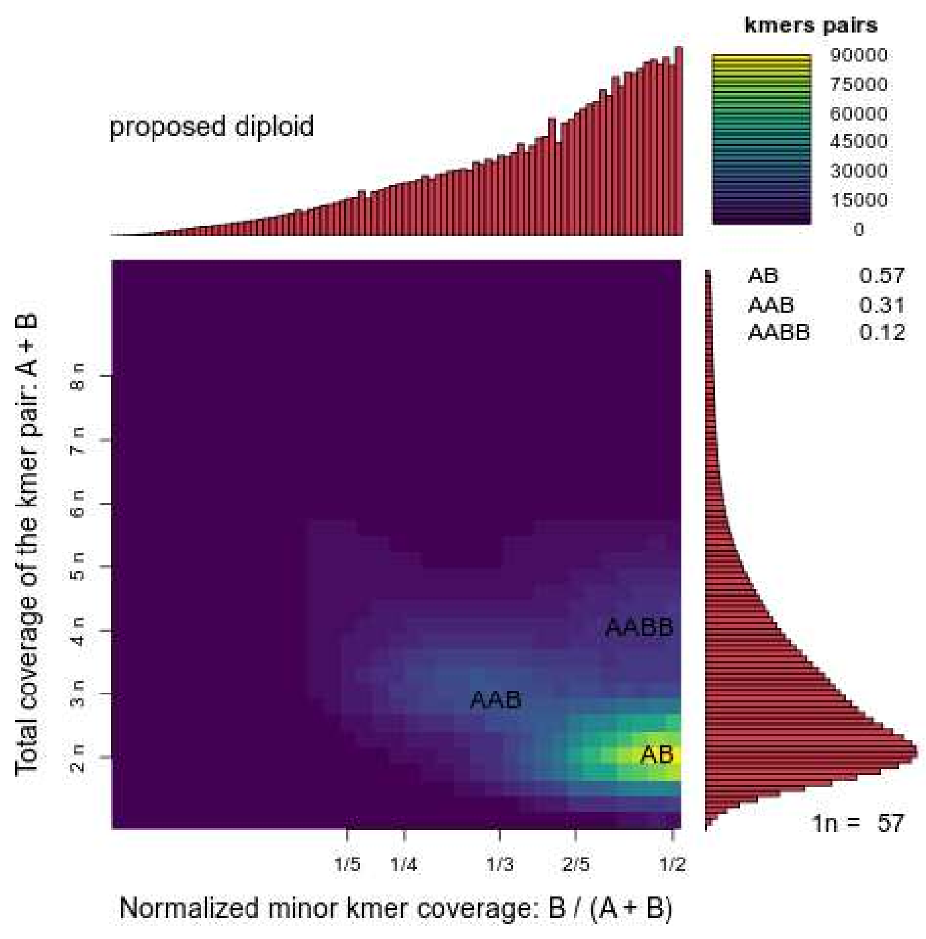

Genome Statistics with gfastats

Metric	asm1	asm2
Total scaffold length	334537900	522622696
GC content %	32.70	32.77
# gaps in scaffolds	0	0
Total gap length in scaffolds	0	0
# scaffolds	320	10048
Largest scaffold	46943257	1003927
Scaffold N50	15967463	124148
Scaffold L50	7	1033
Scaffold L90	37	7058
# contigs	320	10048
Largest contig	46943257	1003927
Contig N50	15967463	124148
Contig L50	7	1033
Contig L90	37	7058

K-mer Multiplicity asm1 Only (Flat)

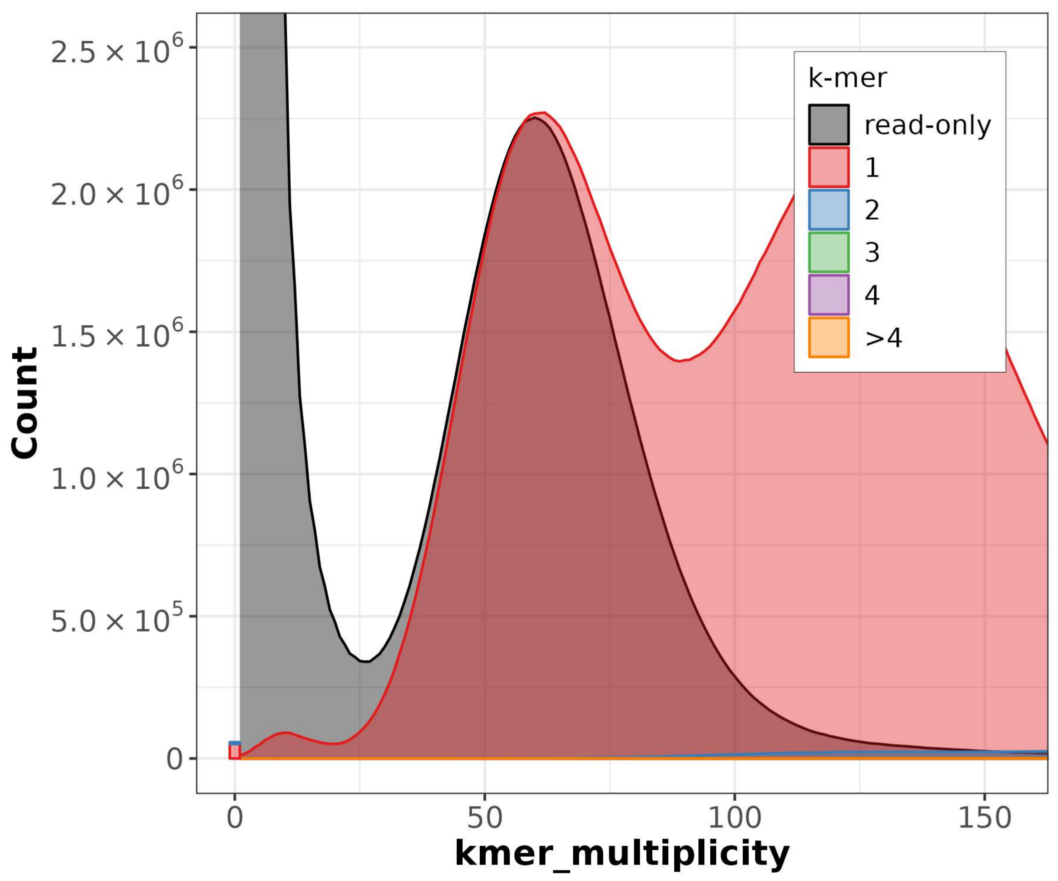

K-mer Multiplicity asm1 + asm2 (Flat)

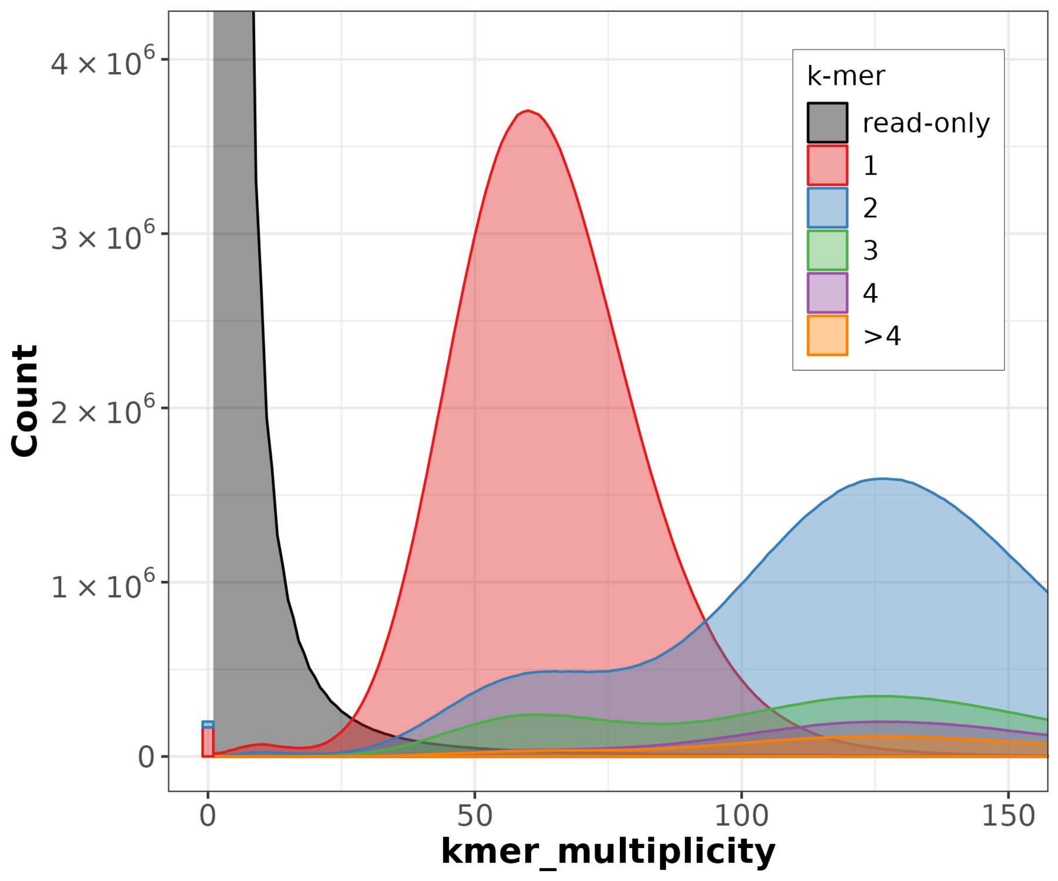

Assembly ID: Scapyr_13_hic

Database built with kmer size: 21 bp

Number of haploptypes/assemblies: 2

Expected genome size: 300901319

Heterozygosity: 2.322 %

QV Score

assembly_name	Assembly_Only	Total_Kmers	QV_Score
Scapyr_13_hic.asm1	47084	338324647	51.7865
Scapyr_13_hic.asm2	43535	336518307	52.1036
Both	90619	674842954	51.9417

Kmer Completeness

assembly_name	Kmers_Assembly	Kmers_Reads	%
Scapyr_13_hic.asm1	266932308	364489292	73.2346
Scapyr_13_hic.asm2	266421483	364489292	73.0945
both	359315262	364489292	98.5805

BUSCOv5 (database: insecta)

Metric	asm1 #	asm1 %	asm2 #	asm2 %
Complete	1353	99.0	1353	99.0
Complete and single-copy	1341	98.1	1348	98.1
Complete and duplicated	12	0.9	5	0.9
Fragmented	1	0.1	2	0.1
Missing	13	0.9	12	0.9
Total searched groups	1367	1367.0	1367	1367.0

Genomescope2 Profile (Linear)

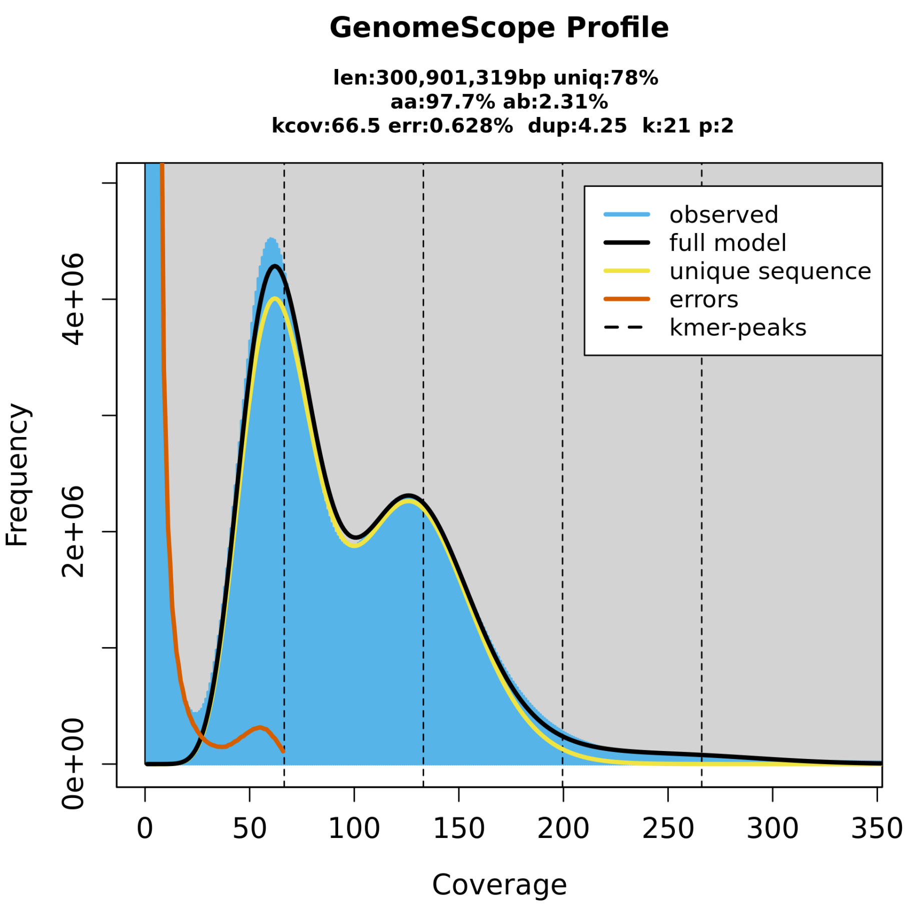

Smudgeplot Profile

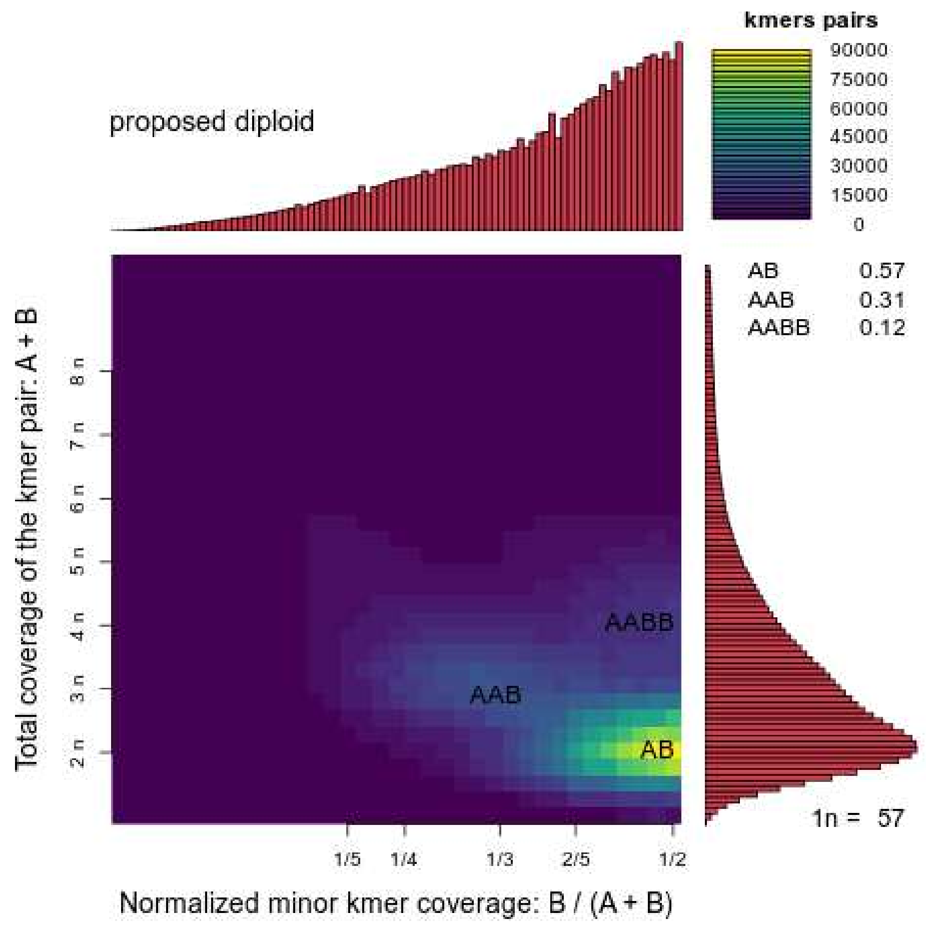

Genome Statistics with gfastats

Metric	asm1	asm2
Total scaffold length	338333647	336523867
GC content %	32.72	32.87
# gaps in scaffolds	0	0
Total gap length in scaffolds	0	0
# scaffolds	450	278
Largest scaffold	43973524	24357284
Scaffold N50	9964336	14935200
Scaffold L50	9	9
Scaffold L90	57	50
# contigs	450	278
Largest contig	43973524	24357284
Contig N50	9964336	14935200
Contig L50	9	9
Contig L90	57	50

K-mer Multiplicity asm1 Only (Flat)

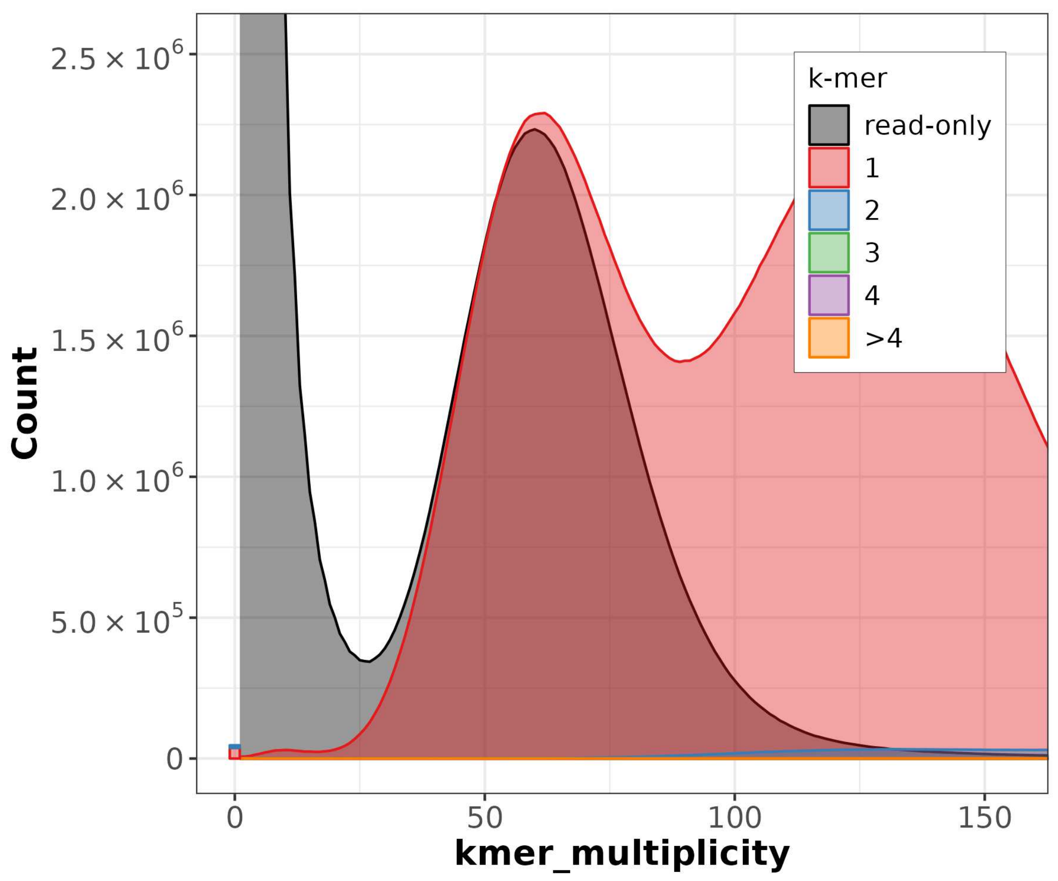

K-mer Multiplicity asm1 + asm2 (Flat)

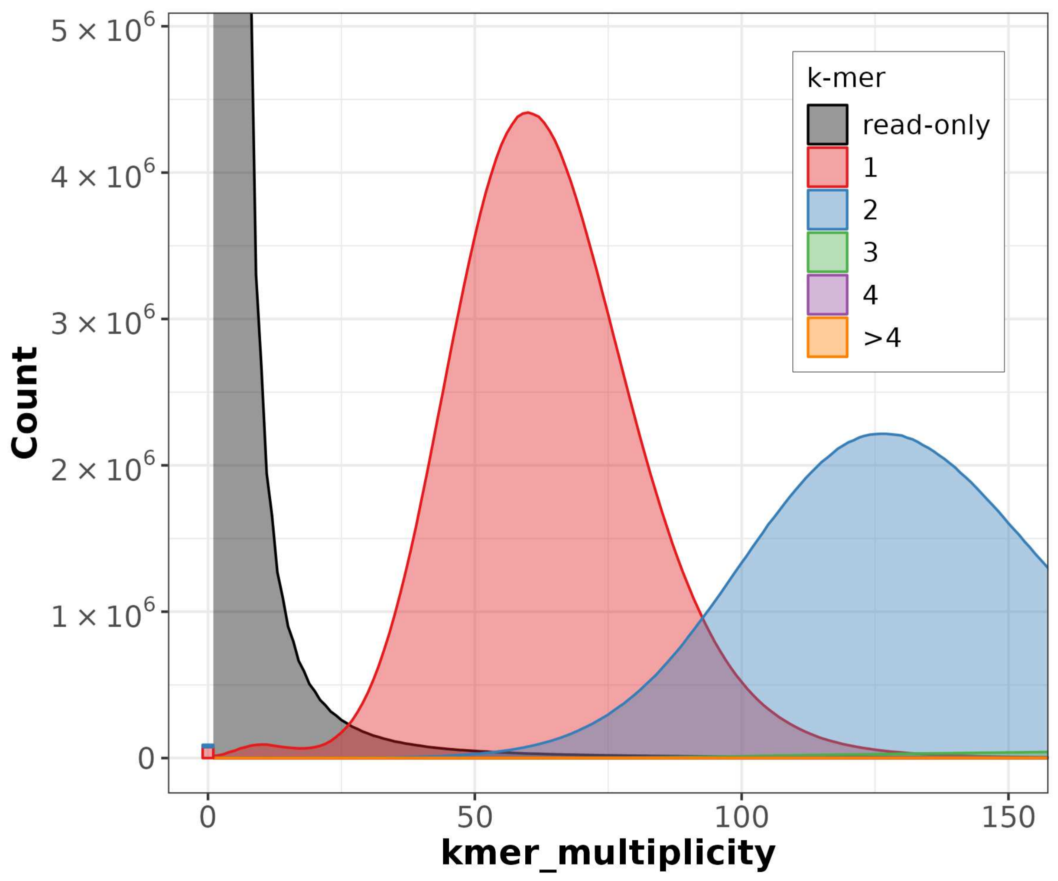

Scapyr__10 HiC Heatmap

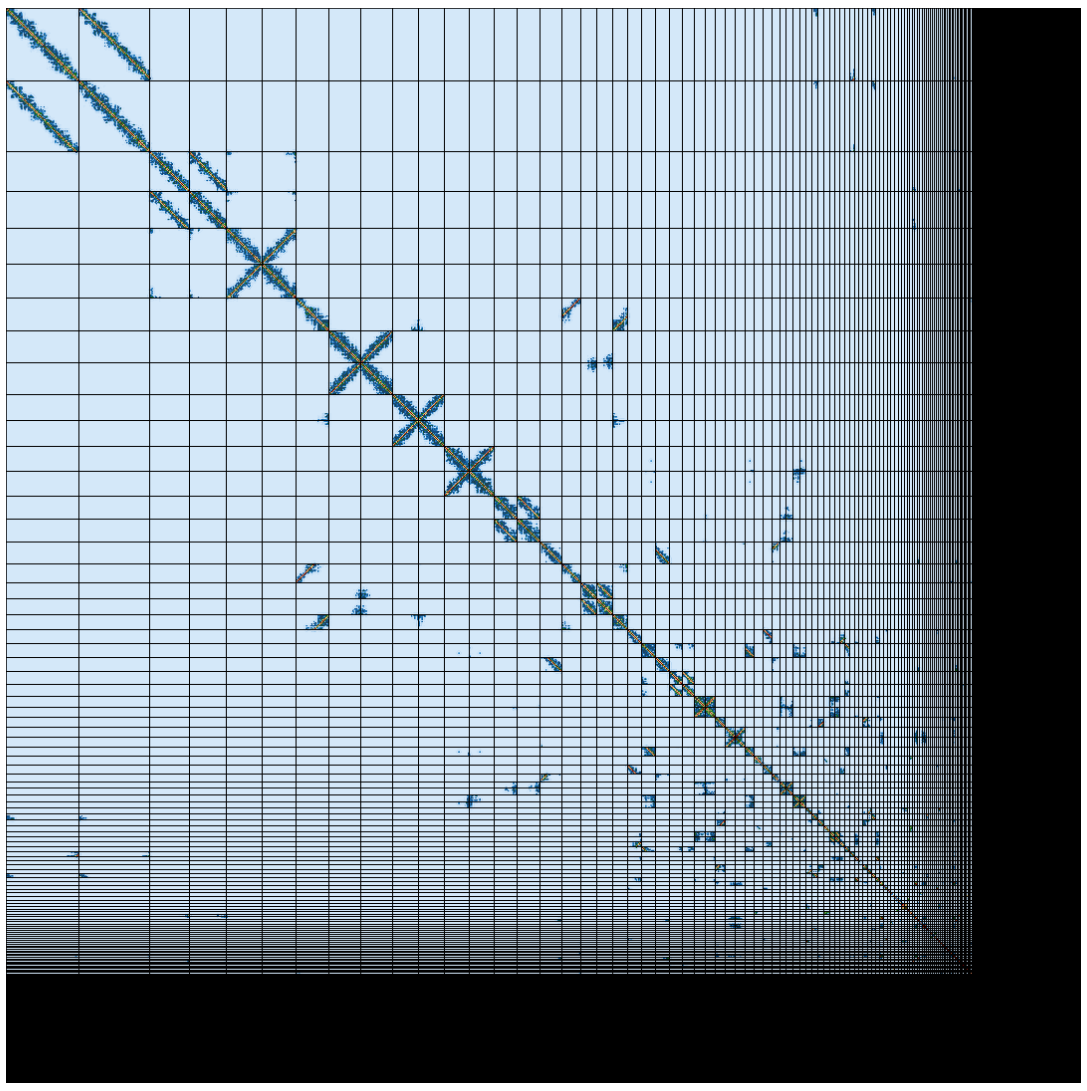

Scapyr__11__hic HiC Heatmap

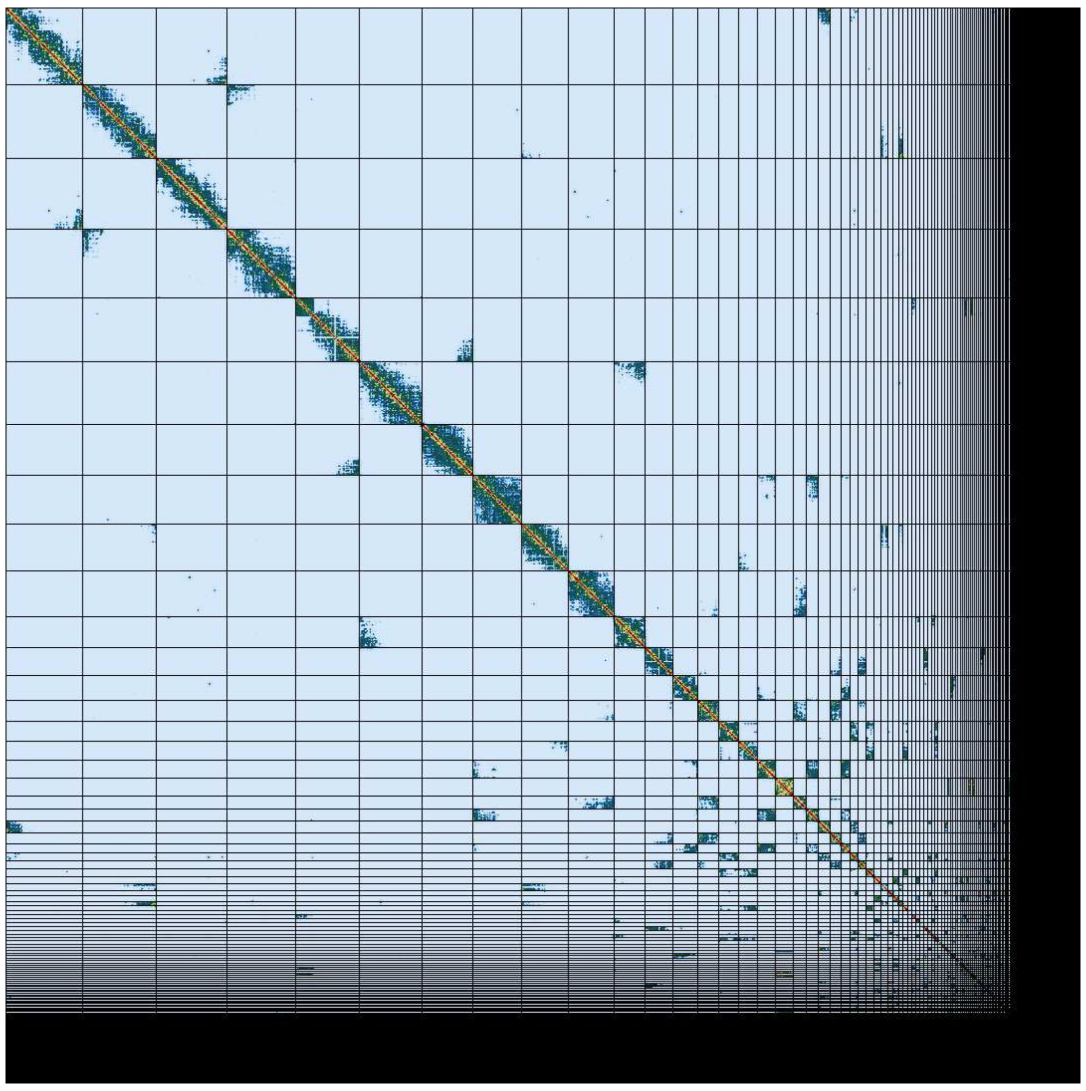

Scapyr__10__hic HiC Heatmap

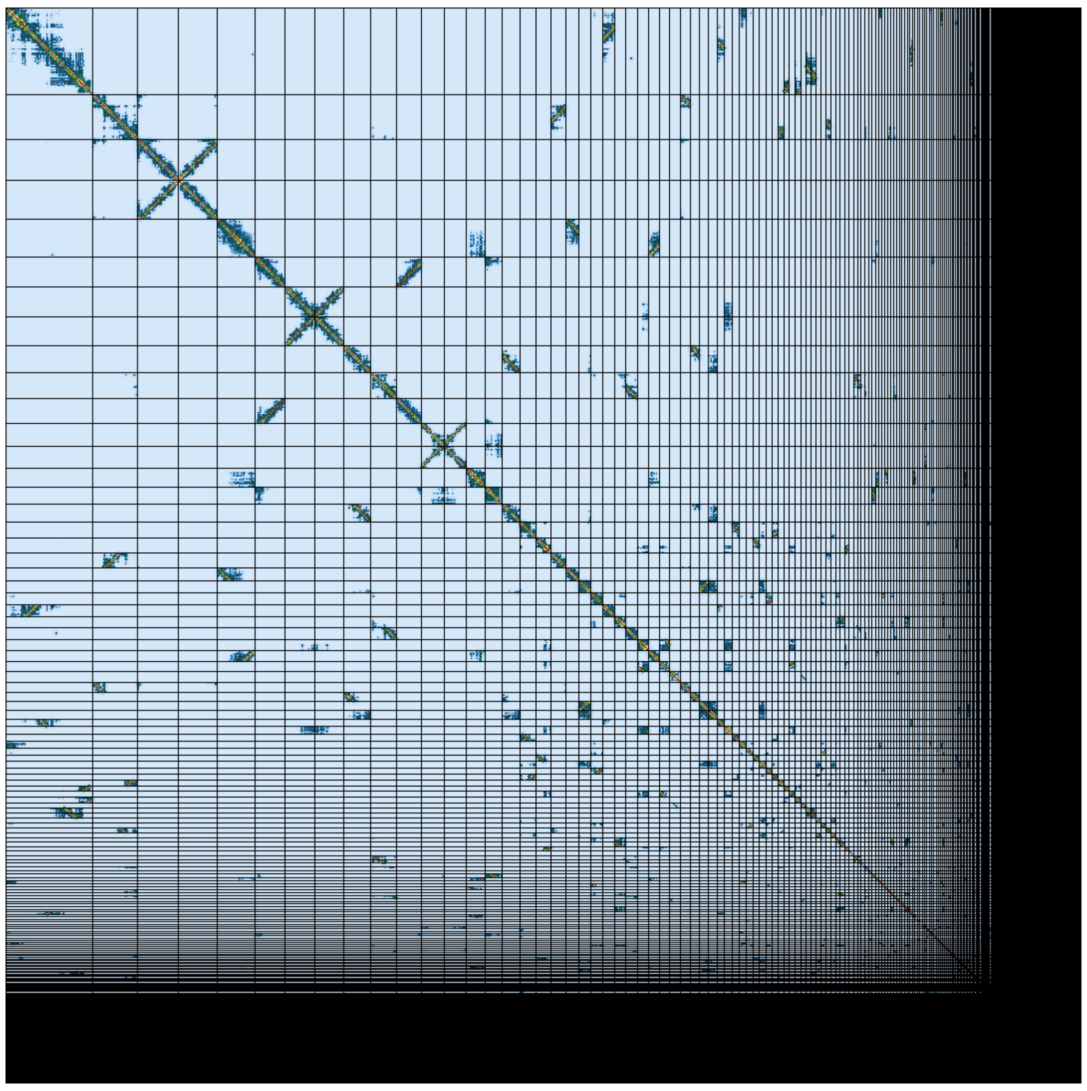

Scapyr__12 HiC Heatmap

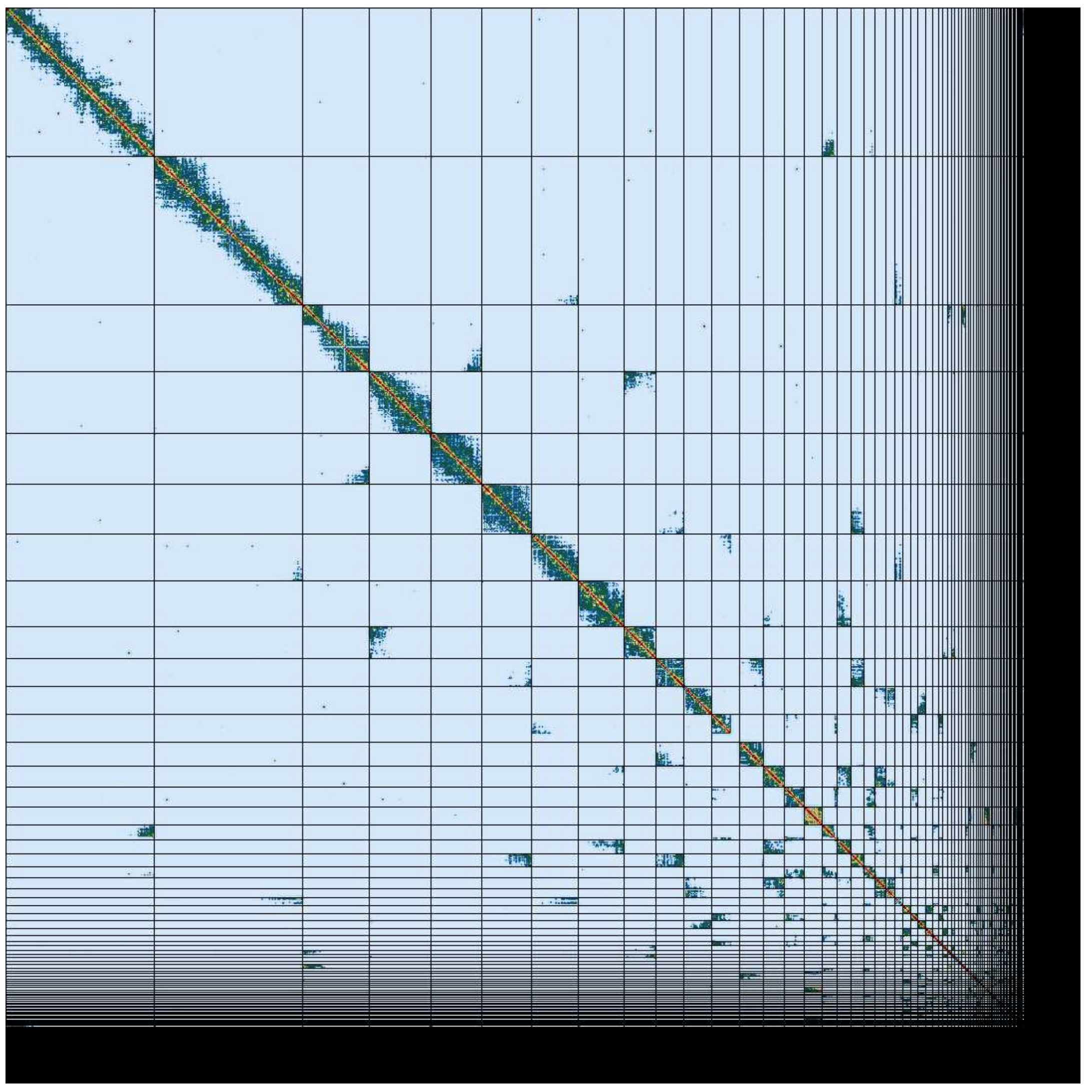

Scapyr__11 HiC Heatmap

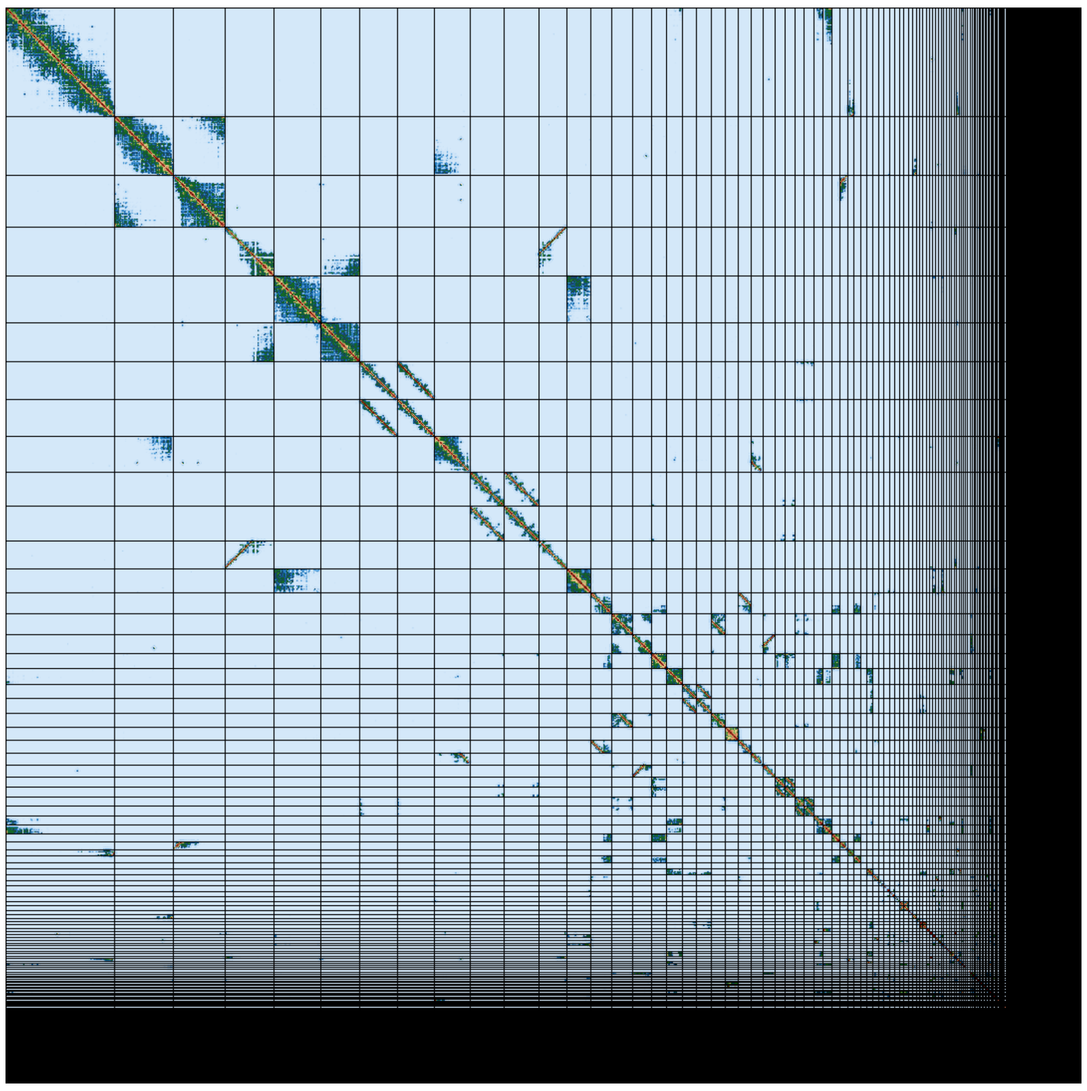

Scapyr__12__hic HiC Heatmap

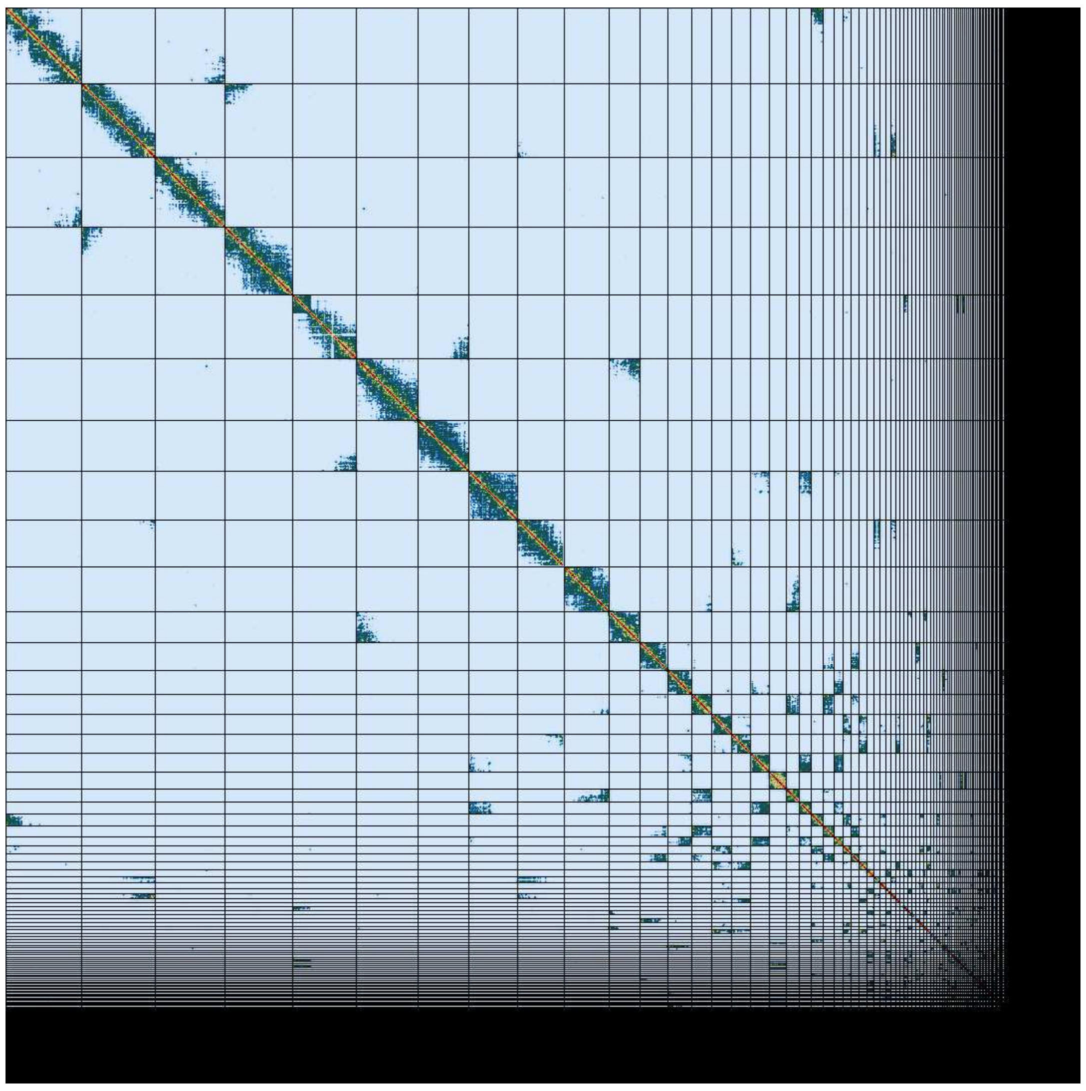

Scapyr__13 HiC Heatmap

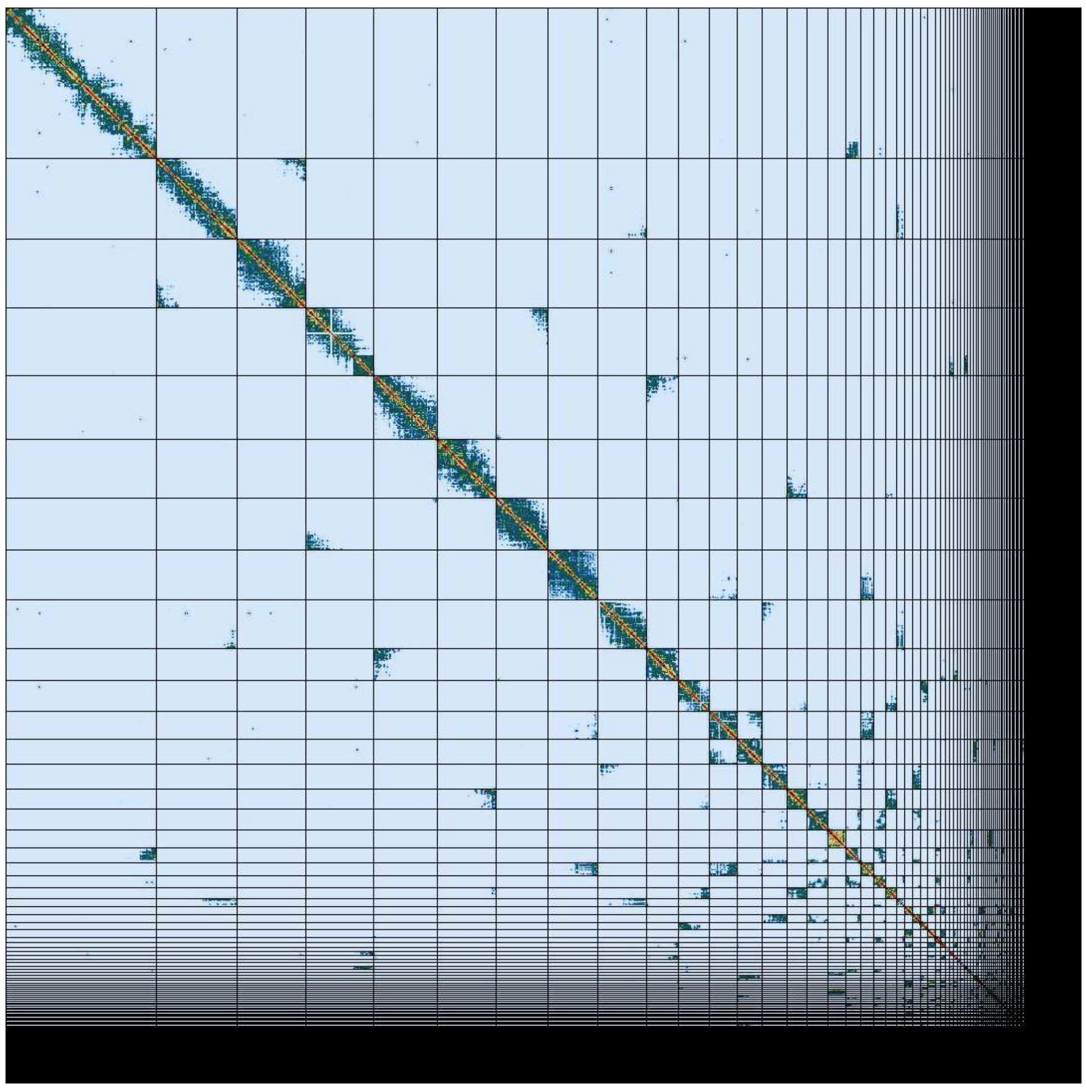

Scapyr__13__hic HiC Heatmap

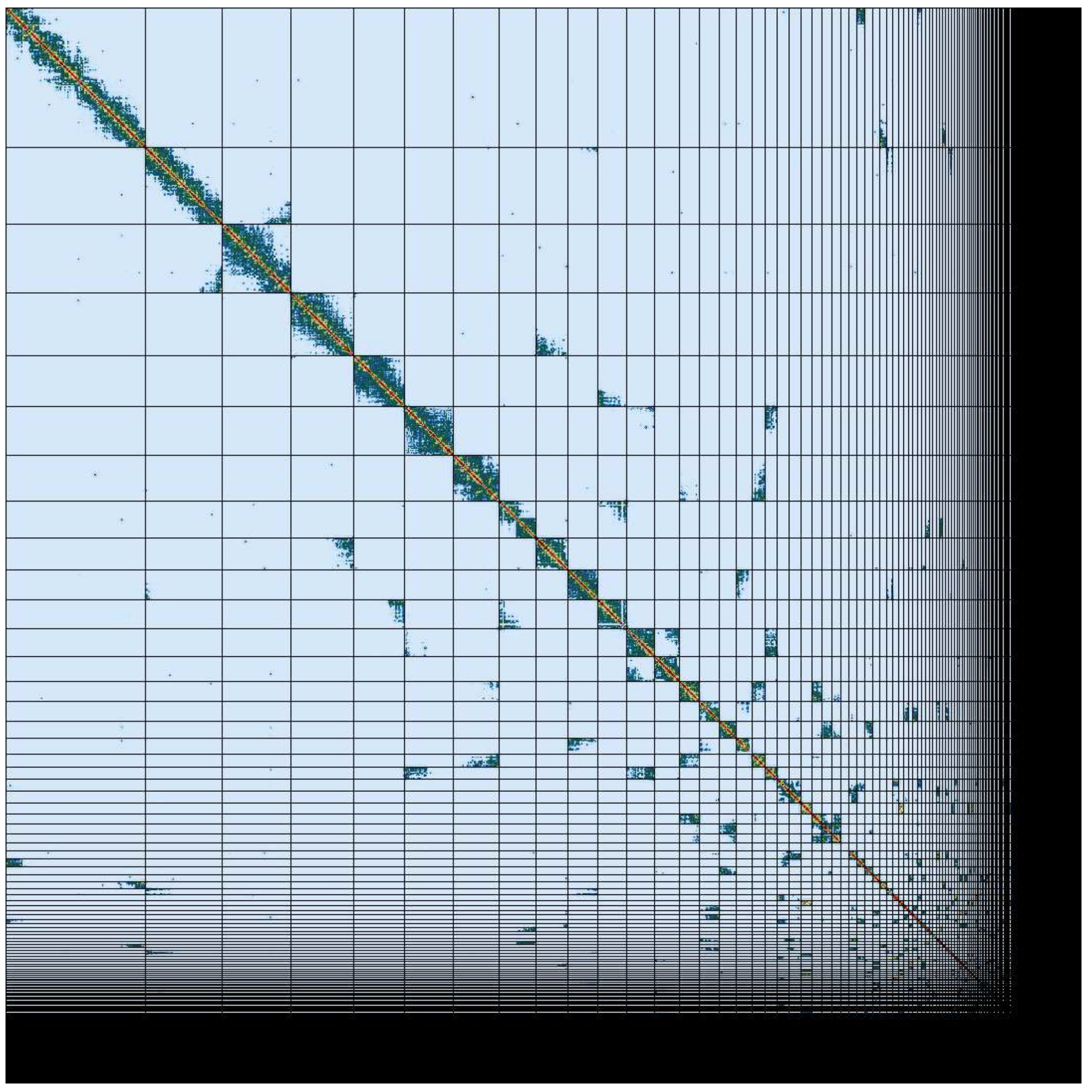

ASM_ID	Total_bp	GC_%	Gaps_per_Gb	Scaf	Cont	Longest_scaf	Scaf_N50	Scaf_L50	Scaf_N90	Longest_cont	Cont_N50	Cont_L50	Cont_N90	qv	Kmer_Compl	Comp_BUSCOs_%	Comp_Single_BUSCOs_%
Scapyr_10.asm1	667072864	32.80	0	632	632	45470904	13479259	16	716538	45470904	13479259	16	716538	51.9855	98.5781	99.2	1.5
Scapyr_10.asm2	157865476	32.49	0	7161	7161	270827	22430	2924	17239	270827	22430	2924	17239	44.7178	23.5437	27.6	23.4
Scapyr_10_hic.asm1	562006054	32.73	0	565	565	45426467	7728830	18	716538	45426467	7728830	18	716538	53.0945	90.7396	99.1	19.0
Scapyr_10_hic.asm2	552197406	32.75	0	390	390	43841759	6998311	19	847098	43841759	6998311	19	847098	54.0414	90.7065	99.1	20.6
Scapyr_11.asm1	447102587	32.74	0	447	447	45470852	11602984	12	802274	45470852	11602984	12	802274	51.3510	80.6270	99.0	61.2
Scapyr_11.asm2	395073435	32.75	0	8845	8845	1003927	90830	910	19783	1003927	90830	910	19783	48.0860	59.9361	73.0	56.8
Scapyr_11_hic.asm1	339525858	32.80	0	412	412	24357256	14822151	9	637722	24357256	14822151	9	637722	50.5988	73.1391	99.0	98.6
Scapyr_11_hic.asm2	331904165	32.75	0	238	238	43973524	11603031	8	847098	43973524	11603031	8	847098	55.4275	73.1626	99.0	98.4
Scapyr_12.asm1	338455103	32.74	0	343	343	46943257	14912490	7	851323	46943257	14912490	7	851323	50.7647	73.1127	98.9	98.5
Scapyr_12.asm2	518055150	32.74	0	9971	9971	1003927	124705	1023	20489	1003927	124705	1023	20489	48.9067	77.1957	98.8	74.1
Scapyr_12_hic.asm1	342421346	32.83	0	492	492	24357256	14822151	9	575215	24357256	14822151	9	575215	50.1603	73.1556	99.0	98.3
Scapyr_12_hic.asm2	333240511	32.76	0	261	261	43973510	11603031	8	807069	43973510	11603031	8	807069	55.1985	73.1757	99.0	98.3
Scapyr_13.asm1	334537900	32.70	0	320	320	46943257	15967463	7	851323	46943257	15967463	7	851323	50.8729	72.7337	98.3	97.9
Scapyr_13.asm2	522622696	32.77	0	10048	10048	1003927	124148	1033	20496	1003927	124148	1033	20496	48.8630	72.7337	98.8	73.6
Scapyr_13_hic.asm1	338333647	32.72	0	450	450	43973524	9964336	9	626247	43973524	9964336	9	626247	51.7865	73.2346	99.0	98.1
Scapyr_13_hic.asm2	336523867	32.87	0	278	278	24357284	14935200	9	666786	24357284	14935200	9	666786	52.1036	73.2346	99.0	98.6

Total_bp	GC_%	Gaps_per_Gb	Scaf	Cont	Longest_scaf	Scaf_N50	Scaf_L50	Scaf_N90	Longest_cont	Cont_N50	Cont_L50	Cont_N90	qv	Kmer_Compl	Comp_BUSCOs_%	Comp_Single_BUSCOs_%
Max	Max	Max	Max	Max	Max	Max	Max	Max	Max	Max	Max	Max	Max	Max	Max	Max
Min	Min	Min	Min	Min	Min	Min	Min	Min	Min	Min	Min	Min	Min	Min	Min	Min

ASM_ID	Gaps_per_Gb	Scaf_N50	Cont_N50	qv	Kmer_Compl	Comp_Single_BUSCOs_%
Scapyr_10.asm1	0	13479259	13479259	51.9855	98.5781	1.5
Scapyr_10.asm2	0	22430	22430	44.7178	23.5437	23.4
Scapyr_10_hic.asm1	0	7728830	7728830	53.0945	90.7396	19.0
Scapyr_10_hic.asm2	0	6998311	6998311	54.0414	90.7065	20.6
Scapyr_11.asm1	0	11602984	11602984	51.3510	80.6270	61.2
Scapyr_11.asm2	0	90830	90830	48.0860	59.9361	56.8
Scapyr_11_hic.asm1	0	14822151	14822151	50.5988	73.1391	98.6
Scapyr_11_hic.asm2	0	11603031	11603031	55.4275	73.1626	98.4
Scapyr_12.asm1	0	14912490	14912490	50.7647	73.1127	98.5
Scapyr_12.asm2	0	124705	124705	48.9067	77.1957	74.1
Scapyr_12_hic.asm1	0	14822151	14822151	50.1603	73.1556	98.3
Scapyr_12_hic.asm2	0	11603031	11603031	55.1985	73.1757	98.3
Scapyr_13.asm1	0	15967463	15967463	50.8729	72.7337	97.9
Scapyr_13.asm2	0	124148	124148	48.8630	72.7337	73.6
Scapyr_13_hic.asm1	0	9964336	9964336	51.7865	73.2346	98.1
Scapyr_13_hic.asm2	0	14935200	14935200	52.1036	73.2346	98.6

Gaps_per_Gb	Scaf_N50	Cont_N50	qv	Kmer_Compl	Comp_Single_BUSCOs_%
< 200	> 100Mbp	> 10Mbp	> 50	> 95%	> 95%
200 - 1000	10Mbp - 100Mbp	1Mbp - 10Mbp	40 - 50	90% - 95%	90% - 95%
1000 - 10000	0.1Mbp - 10Mbp	0.01Mbp - 1Mbp	35 - 40	80% - 90%	80% - 90%
> 10000	< 0.1Mbp	< 0.01Mbp	< 35	< 80%	< 80%
